# 1,10a-Dihydro-1-aza-10a-boraphenanthrene and 6a,7-Dihydro-7-aza-6a-boratetraphene: Two New Fluorescent BN-PAHs

**DOI:** 10.1021/acs.joc.1c01095

**Published:** 2021-11-22

**Authors:** Isabel Valencia, Patricia García-García, David Sucunza, Francisco Mendicuti, Juan J. Vaquero

**Affiliations:** † Departamento de Química Orgánica y Química Inorgánica, Instituto de Investigación Química “Andrés M. del Río” (IQAR), Universidad de Alcalá, IRYCIS, Campus Científico-Tecnológico, 28805 Alcalá de Henares, Spain; ‡ Departamento de Química Analítica, Química Física e Ingeniería Química, Instituto de Investigación Química “Andrés M. del Río” (IQAR), Universidad de Alcalá, Campus Científico-Tecnológico, 28805 Alcalá de Henares, Spain

## Abstract

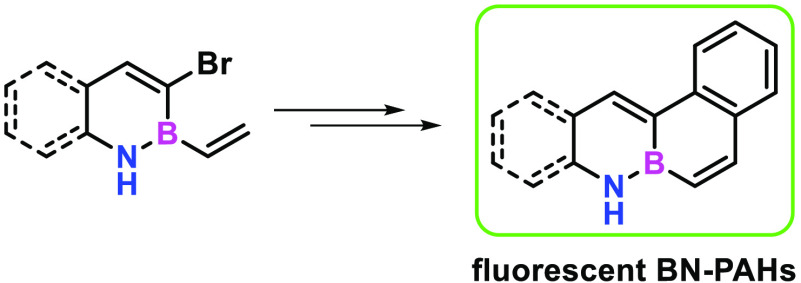

Previously unknown 1,10a-dihydro-1-aza-10a-boraphenanthrene and 6a,7-dihydro-7-aza-6a-boratetraphene have been efficiently synthesized. Bromination of these BN-PAHs proceeds with complete regioselectivity, resulting in the formation of different substituted derivatives via cross-coupling reactions. These compounds exhibit rather high fluorescence quantum yields (up to ϕ_F_ = 0.80).

## Introduction

BN/CC-isosterism in aromatic compounds leads to BN-polycyclic aromatic hydrocarbons (BN-PAHs),^1^ which retain their aromaticity but exhibit different properties as a result of a dipole in the molecule.^2^ This formal replacement of a C=C unit by an isoelectronic B–N bond has been exploited for the design of new materials. Thus, BN-arenes have been investigated as promising components for improved optoelectronic devices,^3^ as well as in the search for new pharmacophores in medicinal chemistry^4^ and in the development of novel ligands for transition metal-based catalysis.^5^

As a result of the significant progress seen in the field of BN-PAHs over the past few years, several BN-arenes have been prepared in sufficient quantities, thus facilitating further studies into the properties of these heterocycles.^6^ Nevertheless, as these examples cover only a small part of all the possible permutations of this BN/CC-isosterism, a basic understanding of the simplest of these systems is still highly desirable.

**Figure 1:**
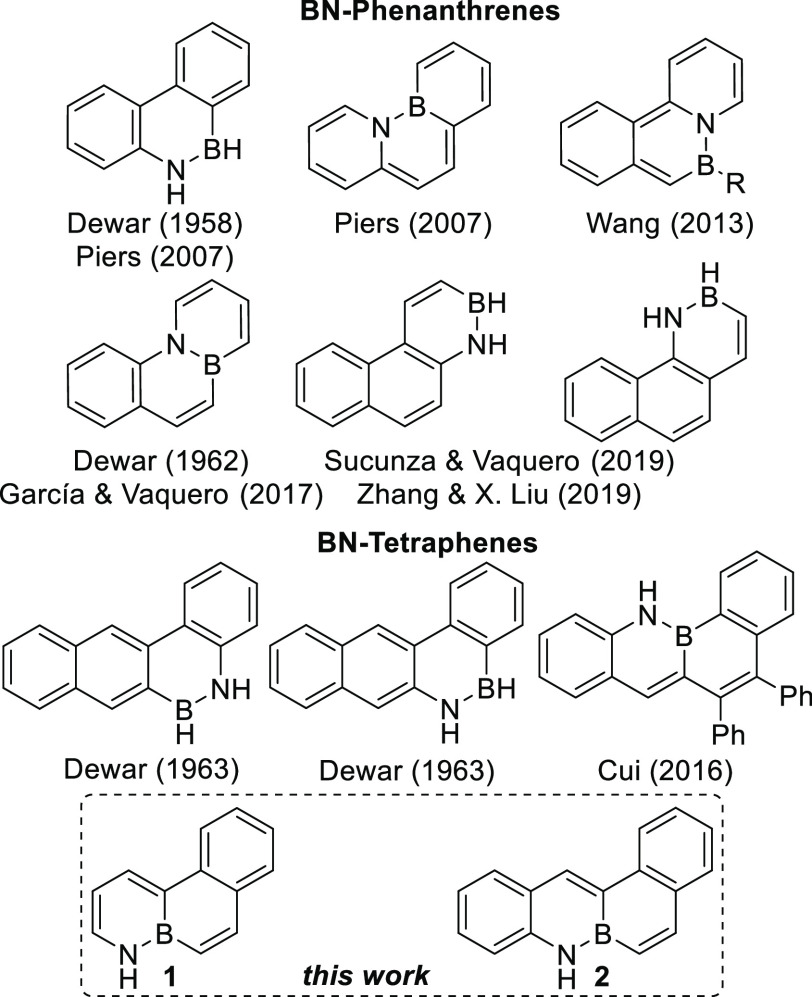
BN-phenanthrenes and BN-tetraphenes.

In this regard, several BN-isosteres of mono-, bi-, tri-, and tetracyclic aromatic compounds have been reported.^7^ In particular, with respect to tri- and tetracyclic BN-PAHs, different anthracene,^8^ phenanthrene,^9^ tetracene,^10^ tetraphene,^11^ chrysene,^12^ pyrene,^12b,13^ benzo[*c*]phenanthrene,^14^ and triphenylene^15^ analogues in which a C=C unit has been replaced by a B–N bond have been described, showing that the position of the B–N unit has a crucial effect on both their reactivity and photophysical properties.^7,9a−9f,16^ Herein, we report an efficient synthesis for two novel systems, namely, isosteres of phenanthrene and tetraphene (Figure 1), as well as their derivatization via a bromination-cross coupling reaction methodology and a study of their main optical properties.

## Results and Discussion

**Scheme 1:**
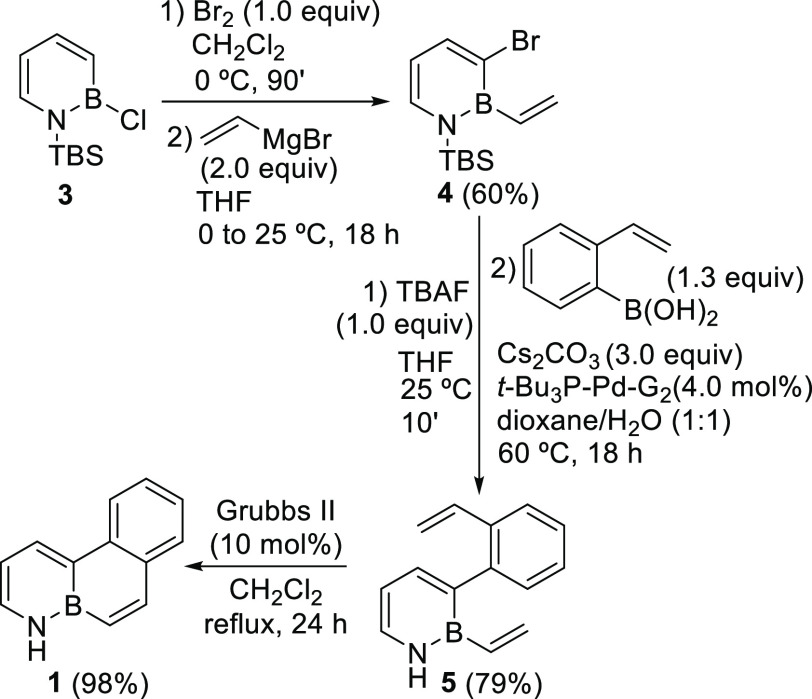
Synthesis of 1,10a-Dihydro-1-aza-10a-boraphenanthrene **1**

The synthesis of BN-phenanthrene **1** (Scheme 1) started with regioselective bromination of the commercially available monocyclic BN-arene **3**(17) and subsequent treatment with two equivalents of vinylmagnesium bromide to give **4**. Removal of the *tert*-butyldimethylsilyl ether (TBS)-protecting group from this substrate, followed by a Suzuki–Miyaura cross-coupling reaction, using chloro[(tri-*tert*-butylphosphine)-2-(2-aminobiphenyl)] palladium(II) (*t*Bu_3_P–Pd-G2) as a catalyst and Cs_2_CO_3_ as a base,^18^ afforded biphenyl derivative **5**. Finally, a ring-closing metathesis of this intermediate using the second-generation Grubbs catalyst gave the desired compound **1**. Altogether, this novel BN-phenanthrene was prepared in five steps, with only three purifications, in 46% overall yield.

**Scheme 2:**
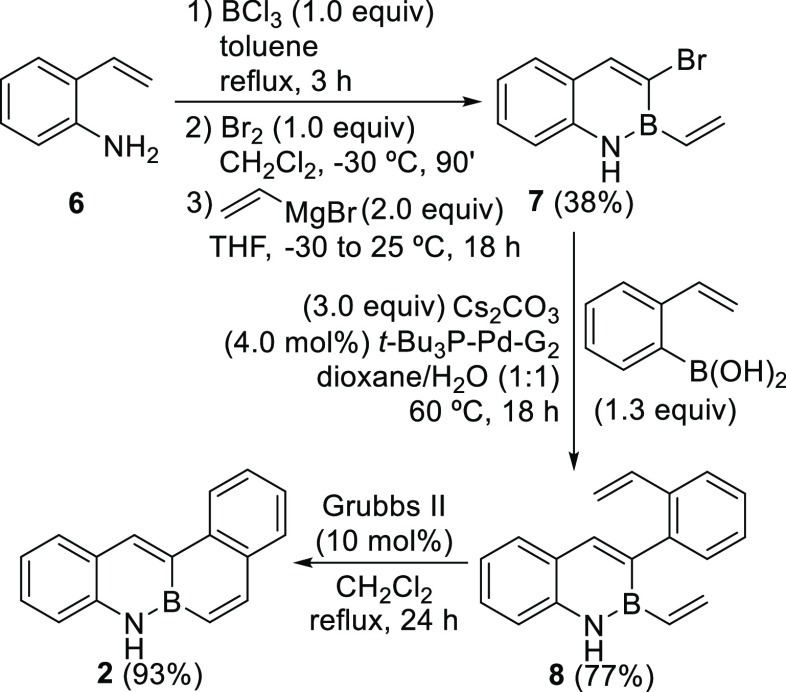
Synthesis of 6a,7-Dihydro-7-aza-6a-boratetraphene **2**

**Figure 2:**
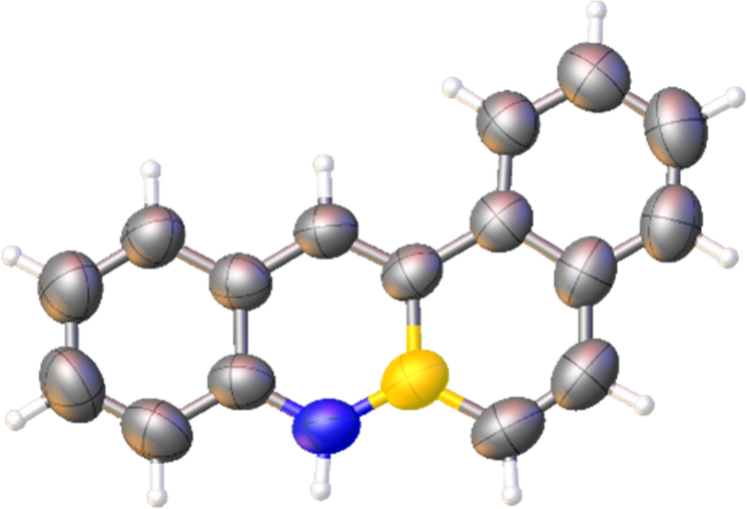
X-ray structure of BN-tetraphene **2** (ellipsoids at the 50% probability level).

BN-tetraphene **2** was prepared in five steps (three purification processes), using a slightly modified methodology, in 27% overall yield (Scheme 2). Thus, this synthesis started with the formation of the bicyclic BN-arene **7** via the treatment of 2-vinylaniline **6** with boron trichloride to force a borylative cyclization,^19^ regioselective bromination,^18^ and nucleophilic substitution at the boron position using vinylmagnesium bromide. A subsequent Suzuki–Miyaura cross-coupling reaction with intermediate **7**, using *t*Bu_3_P–Pd-G2 as a catalyst and Cs_2_CO_3_ as a base, and a final ring-closing metathesis using the second-generation Grubbs catalyst, afforded compound **2**. The structure of this compound, as confirmed by an X-ray diffraction study (see Supporting Information),^20^ showed a B–N bond length similar to those reported for other BN-aromatic compounds (Figure 2).^7^

**Scheme 3:**
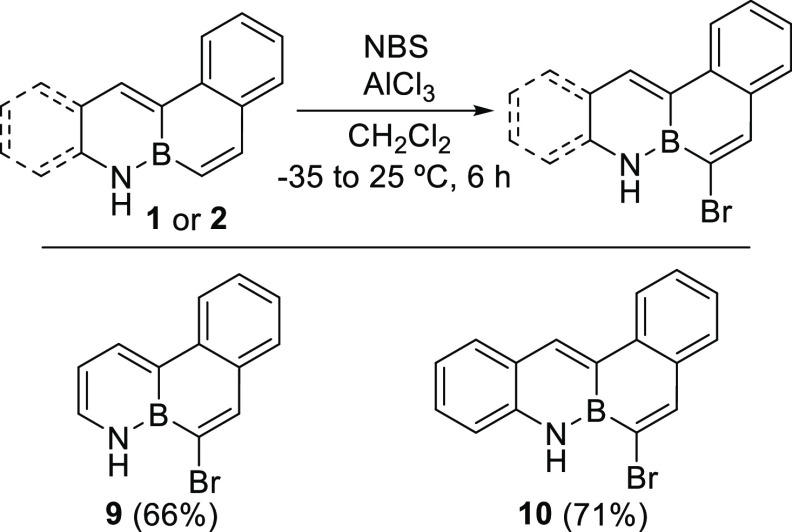
Regioselective Bromination of **1** and **2**

The reactivity of both BN-phenanthrene **1** and BN-tetraphene **2** was explored to obtain functionalized derivatives. Thus, we evaluated their behavior in the presence of brominating agents as electrophilic aromatic substitution is a well-established tool for the functionalization of BN-PAHs.^7^ In this regard, although the use of Br_2_ in CH_2_Cl_2_ was not successful, regioselective bromination was achieved at the carbon next to the boron, the most reactive position in related BN-aromatics according to the literature,^7^ when compounds **1** and **2** were treated with NBS/AlCl_3_ in CH_2_Cl_2_ (Scheme 3). Under these reaction conditions, no traces of other regioisomers or dibrominated compounds were observed.

**Scheme 4:**
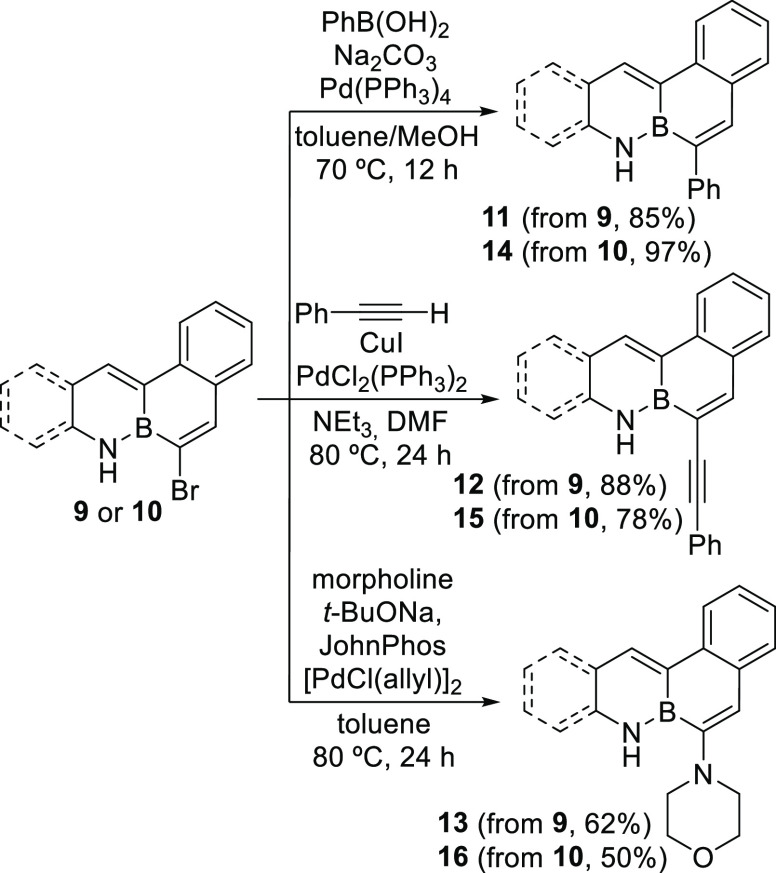
Cross-Coupling Reactions

Bromo-substituted BN-arenes **9** and **10** are suitable for further functionalization by palladium-catalyzed cross-coupling reactions. Thus, standard Suzuki, Sonogashira, and Buchwald–Hartwig amination coupling conditions were employed to obtain phenyl-, alkynyl-, and morpholinyl-substituted derivatives **11**–**16** in high yields (Scheme 4).

**Scheme 5:**
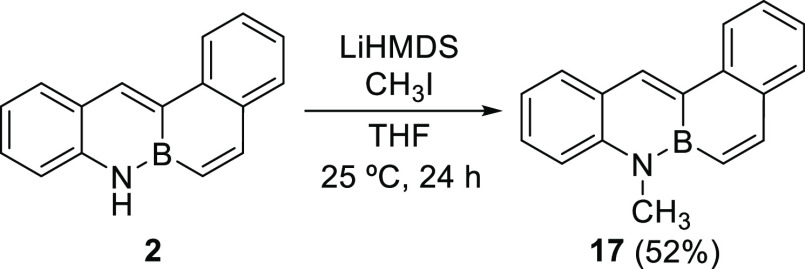
Alkylation of BN-Tetraphene **2**

Alkylation of BN-tetraphene **2** was also tested, with moderate success (Scheme 5). Thus, treatment of this BN-PAH with two equivalents of the base lithium bis(trimethylsilyl)amide (LiHMDS) and iodomethane led to the formation of methylated BN-tetraphene derivative **17** in 52% yield.

Once the efficient synthesis for BN-phenanthrene **1** and BN-tetraphene **2** had been developed and various functionalized derivatives were synthesized, we focused on the evaluation of their main photophysical properties. The absorption and emission data for parent compounds **1** and **2** and their derivatives **11**–**16** in cyclohexane are summarized in Table 1.

**Table 1:** UV/Vis and Fluorescence Parameters for BN-PAHs **1**, **2**, and **11**–**16**^a^

cmpnd	ε (10^–3^ × M^–1^ cm^–1^)^b^	λ_abs max_ (λ_exc_) (nm)^c^	λ_em_ (nm)	ϕ_F_^d^	τ (ns)^e^
**1**	9.6	338, 354, 372 (354)	395	0.33	1.9
**11**	12.0	342, 358, 377 (343)	405	0.47	6.9
**12**	19.1	350, 366, 386 (351)	432	0.63	6.1
**13**	4.9	350, 365(s) (350)	388 (521)	0.21	2.3 (13.3)
**18**	11.1	244(s), 251, 274, 281, 293, 315, 323, 330, 337, 346 (293)	365	0.01	15.5^f^
**2**	24.1	348, 365, 385 (365)	410	0.68	4.1
**14**	22.7	351, 368, 387 (368)	441	0.80	8.9
**15**	28.8	357, 375, 394 (375)	463	0.66	7.1
**16**	15.3	342, 359, 376 (323)	364 (545)	0.17	1.7 (12.3)
**19**	5.7	255, 267, 277, 287, 299(s) 313, 327, 340, 358 (358)	386	0.02	15.0

a Cyclohexane was used as a solvent.

b Molar absorptivities measured at λ_exc_.

c Peaks (maxima of the band to the red in black) and shoulders (s) for the bands that appear to the red.

d Standard for fluorescence quantum yield was 9,10-diphenylanthracene in cyclohexane (ϕ_F_ = 0.93).^21^

e Fluorescence lifetimes were obtained upon 335 nm (or 296 nm)

f Nanoled excitation by fixing the emission at λ_em_.

**Figure 3:**
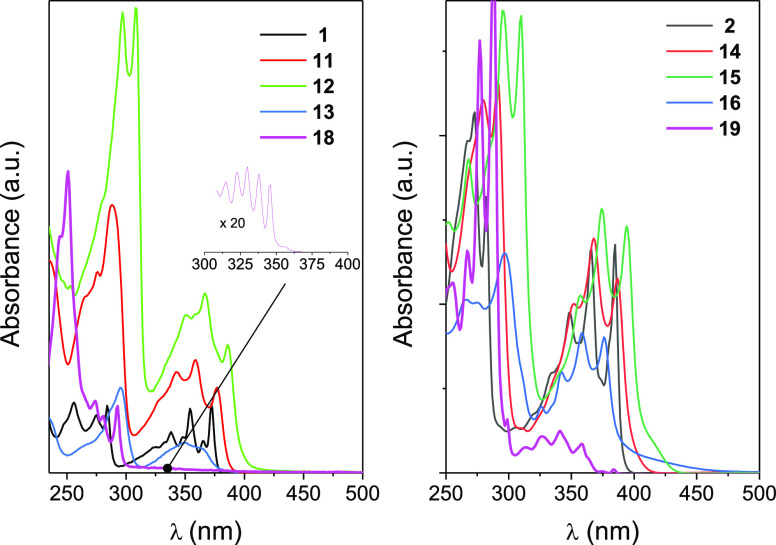
UV/vis absorption spectra for (left) **1**, **11**–**13** BN-phenanthrene and (right) **2**, **14**–**16** BN-tetraphene derivatives as well as phenanthrene (**18**) and tetraphene (**19**) in dilute solutions of cyclohexane at 25 °C. Superimposed is the enlargement of the low energy band for **18**.

UV–vis absorption spectra for BN-phenanthrenes **1**, **11**–**13** and BN-tetraphenes **2**, **14**–**16** as well as their PAH phenanthrene (**18**) and tetraphene (**19**) isostere analogues of **1** and **2** derivatives, respectively, were monitored in the 250–500 nm range. All spectra show two main structureless bands (Figure 3 and Figure S1); however, both bands for **18** and **19** are shifted slightly to the blue relative to those for BN-phenanthrene and BN-tetraphene derivatives. Besides, less energetic bands for **18** and **19** are much less intense. The presence of the fourth aromatic ring in **19** and the BN-tetraphenes favors ring conjugation, shifting all spectra by about 8–14 nm to the red with respect to those obtained for **18** and BN-phenanthrene derivatives. The effect of a larger contribution to the ring conjugation of some substituents over others (H < Ph < PhC≡C) is also the reason for the observed bathochromic displacements of the absorption peaks for the less energetic bands in **11** (**14**) and **12** (**15**) with respect to their parent **1** (**2**). As reported previously, the peaks for the morpholinyl-containing derivatives **13** (**16**) are shifted slightly to the blue with respect to **1** (**2**) (Table 1).^12a^ Larger molar absorptivities were observed for BN-tetraphene derivatives than for their BN-phenanthrene counterparts. **18** and **19** PAH models deviate from this trend.

**Figure 4:**
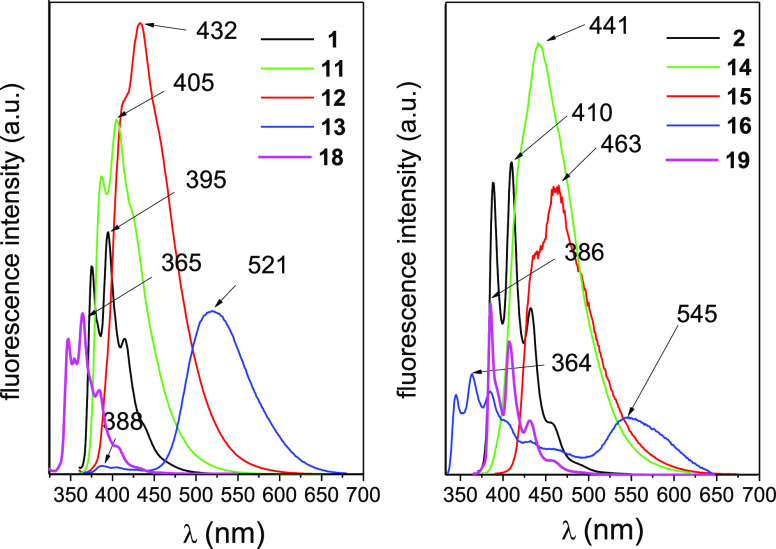
Emission spectra for (left) **1**, **11**–**13** BN-phenanthrene and (right) **2**, **14**–**16** BN-tetraphene derivatives as well as phenanthrene (**18**) and tetraphene (**19**) in cyclohexane at 25 °C. Absorbances at λ_exc_ were below 0.15 in all measurements.

The emission spectra show features similar to the absorption spectra (Figure 4). Thus, conjugation provokes displacement of the emission bands to the red for **18** and BN-tetraphene derivatives with respect to the **19** and BN-phenanthrene ones and the substituent conjugation also affects the emission location in a similar manner by shifting the peaks of **11** (**14**) and **12** (**15**) to the red and **13** (**16**) to the blue relative to **1** (**2**). However, both morpholinyl-containing derivatives **13** and **16** displayed additional broad fluorescence bands centered at 521 and 545 nm, respectively. Both of these bands, the intensity of which depends on the nature of the solvent and which were previously observed in a morpholinyl-functionalized 4a-aza-12a-borachrysene,^12a^ were attributed to the presence of rather stable π–π stacking aggregates in solution (see Supporting Information, pages S10–13, for confirmation).

With the exception of the two morpholinyl-functionalized derivatives (**13** and **16**), the rest of the BN-PAHs studied showed relatively high quantum yields (ϕ_F_ > 0.33), much higher than the PAHs **18** and **19**, whose fluorescence is very weak. In particular, **12**, **2**, **14**, and **15** exhibited rather high fluorescence, with quantum yields of 0.63, 0.68, 0.80, and 0.66, respectively. The effect of phenylalkynyl substituents on the fluorescence increase in BN-aromatic compounds has been reported previously.^12a,16a^ Fluorescence intensity profiles for **18** and **19** and BN-phenanthrene and tetraphene derivatives (Figure S2) were reasonably adjusted to monoexponential decays. Lifetimes (Table 1) are, in general, slightly larger for BN-tetraphenes (∼4–9 ns) than for their corresponding BN-phenanthrene counterparts (∼2–7 ns). PAHs **18** and **19** again deviate from this trend. They show rather high and similar lifetime values near 15 ns.

Additionally, we studied the ability of **1** and **2** to react with *n*-tetrabutylammonium fluoride (TBAF) as the p-orbital of the boron center in BN-PAHs can accept an electron pair from Lewis bases such as F^–^.^9e,12a,22^ To that end, fluorescence titration experiments were carried out on **1** and **2** with TBAF^23^ (Figure S3). The addition of aliquots of fluoride led to a monotonic quenching of the fluorescence intensity in both cases, which can presumably be attributed to the formation of **1** and **2** fluoroborate complexes. The titrations were verified by ^19^F, ^11^B, and ^10^B NMR measurements. Upon addition of 4 equiv of TBAF to BN-phenanthrene **1**, a new signal appeared at −144 ppm in ^19^F NMR and 0 ppm in ^11^B and ^10^B NMR spectra, which could indicate the formation of a fluoroborate complex (Figures S10–S13).^9e^ On the other hand, the comparative analysis of the results from the titrations by TBAF of **2** and the methylated BN-tetraphene derivative **17** (Figures S4 and S5) led us to discard that quenching was due to the F^–^ binding to the NH via hydrogen bonding. The Stern–Volmer plots of fluorescence intensities (Figure S4) and lifetimes (τ_0_/τ = 1 at any [TBAF]) also confirmed that the decrease in fluorescence intensity was due to the likely formation of ground-state fluoroborate complexes. However, these complexes seem to be significantly less stable (Figures S4 and S5) than those reported previously by us for 4a-aza-12a-borachrysene whose complexation constant was a magnitude order larger.^12a^

## Conclusions

Syntheses of the previously unknown compounds 1,10a-dihydro-1-aza-10a-boraphenanthrene and 6a,7-dihydro-7-aza-6a-boratetraphene have been described in five steps (three purification processes). The reactivity of these BN-PAHs with brominating agents was explored in order to obtain functionalized derivatives. Treatment with NBS/AlCl_3_ proceeded with complete regioselectivity, thus allowing subsequent derivatization based on palladium-catalyzed cross-coupling reactions under standard conditions. The fluorescence of these BN-PAHs was also tested, showing rather high fluorescence quantum yields (up to ϕ_F_ = 0.80).

## Experimental Section

### General Methods

Reagents were acquired from commercial sources and used without further purification. When required, solvents were dried using an MBRAUN MB-SPS-800 apparatus. In general, reactions were carried out under an argon atmosphere using oven-dried glassware with magnetic stirring and dry solvents. For reactions that required heating, the heat source was a sand bath. Reactions were monitored using analytical TLC plates (silica gel 60 F254, 0.25 mm), and compounds were visualized with UV radiation. Silica gel grade 60 (70–230 mesh) was used for column chromatography. All melting points were determined in open capillary tubes using a Stuart Scientific SMP3 melting point apparatus (uncorrected). IR spectra were obtained using a PerkinElmer FTIR spectrum 2000 spectrophotometer. ^1^H, ^13^C{^1^H}, and ^11^B{^1^H} NMR spectra were recorded using either a Varian Mercury VX-300, Varian Unity 300, or Varian Unity 500 MHz spectrometer at room temperature. Chemical shifts are given in ppm (δ) downfield from TMS, with calibration with respect to the residual protonated solvent used (δ_H_ = 7.24 ppm and δ_C_ = 77.0 ppm for CDCl_3_). ^11^B{^1^H} NMR spectra were referenced externally to BF_3_·OEt_2_ (δ_B_ = 0 ppm). Coupling constants (*J*) are in hertz (Hz), and signals are described as follows: s, singlet; d, doublet; t, triplet; q, quadruplet; m, multiplet; br, broad; ap, apparent. High-resolution analysis (HRMS) was performed using an Agilent 6210 time-of-flight LC/MS. Absorption spectra were recorded using a Uvikon 941 (Kontron Instruments) UV–vis spectrophotometer. Steady-state fluorescence measurements were carried out using a PTI Quanta Master spectrofluorimeter equipped with a Xenon flash lamp as a light source, single concave grating monochromators, and Glan-Thompson polarizers in the excitation and emission paths. Detection was allowed by a photomultiplier cooled by a Peltier system. Slit widths were selected at 6 nm for both excitation and emission paths, and polarizers were fixed at the “magic angle” condition. Right angle geometry and rectangular 10 mm path cells were used for the fluorescence measurements.

#### 3-Bromo-2-vinyl-1-(*tert*-butyldimethylsilyl)-1,2-dihydro-1,2-azaborine (**4**)

To the Schlenk containing the 1-(*tert*-butyldimethylsilyl)-2-chloro-1,2-dihydro-1,2-azaborine **3** (250 mg, 1.10 mmol, 1.0 equiv) was added anhydrous CH_2_Cl_2_ (5.5 mL, 0.2 M), and the resulting solution was cooled to 0 °C. A recently prepared bromine solution (56 μL, 1.10 mmol, 1.0 equiv) in anhydrous CH_2_Cl_2_ (5.5 mL, 0.2 M) was added under argon at a rate of 1.1 mmol/h. The reaction was stirred for 15 additional minutes at 0 °C and was allowed to warm to room temperature for an hour and a half. The mixture was concentrated under reduced pressure to afford the corresponding intermediate 3-bromo-1-(*tert*-butyldimethylsilyl)-2-chloro-1,2-dihydro-1,2-azaborine as an air- and moisture-sensitive oil, which could be used as is in the next step without further purification. To the Schlenk containing the 3-bromo-1-(*tert*-butyldimethylsilyl)-2-chloro-1,2-dihydro-1,2-azaborine was added anhydrous THF (5.5 mL, 0.2 M), and the resulting solution was cooled to −30 °C. The vinylmagnesium bromide solution (1.0 M in Et_2_O; 2.20 mL, 2.20 mmol, 2.0 equiv) was added dropwise using a syringe, and then the reaction mixture was allowed to warm to room temperature and stirred for 18 h. At the end of the reaction, the mixture was concentrated under reduced pressure, and the remaining residue was purified by flash column chromatography (hexane) to afford the corresponding 3-bromo-2-vinyl-1-(*tert*-butyldimethylsilyl)-1,2-dihydro-1,2-azaborine **4** (194 mg, 0.65 mmol, 60%) as a yellow oil. ^1^H NMR (500 MHz, CDCl_3_): δ (ppm) 7.90 (d, *J* = 7.0 Hz, 1H), 7.29 (d, *J* = 7.0 Hz, 1H), 6.46 (dd, *J* = 20.1, 15.0 Hz, 1H), 6.17 (ap t, *J* = 7.0 Hz, 1H), 5.91 (dd, *J* = 15.0, 3.0 Hz, 1H), 5.80 (dd, *J* = 20.1, 3.0 Hz, 1H), 0.92 (s, 9H), 0.45 (s, 6H). ^13^C{^1^H} NMR (125 MHz, CDCl_3_): δ (ppm) 145.6 (CH), 138.1 (CH), 131.0 (C**), 130.0 (CH), 111.3 (CH), 26.7 (3CH_3_), 1.1 (2CH_3_). **Carbon not observed in ^13^C{^1^H} NMR, assigned by gHMBC. ^11^B{^1^H} NMR (160 MHz, CDCl_3_): δ (ppm) 35.86. HRMS (APCI) calcd for C_12_H_21_BBrNSi [M + H]^+^: 297.0829. Found [M + H]^+^: 297.0828.

#### 3-(2-Vinylphenyl)-2-vinyl-1,2-dihydro-1,2-azaborine (**5**)

Compound **4** (103 mg, 0.347 mmol, 1.0 equiv) was dissolved in 1.7 mL of THF. A TBAF solution (1.0 M; 0.36 mL, 0.364 mmol, 1.0 equiv) was added, and the mixture was stirred for 10 min at room temperature. At the conclusion of the reaction, the solvent was removed under reduced pressure. The resulting crude material was filtered through a pad of silica gel (silica gel, eluent Et_2_O) to afford the corresponding 3-bromo-2-vinyl-1,2-dihydro-1,2-azaborine as a white oil, which could be used as is in the next step without further purification. In an oven-dried Biotage microwave vial equipped with a stir bar, the 3-bromo-2-vinyl-1,2-dihydro-1,2-azaborine (52 mg, 0.28 mmol, 1.0 equiv) and 2-vinylphenylboronic acid (55 mg, 0.37 mmol, 1.3 equiv) were dissolved in dioxane (1.5 mL). The resulting solution was treated with a suspension of cesium carbonate (277 mg, 0.85 mmol, 3.0 equiv) in distilled water (1.0 mL) before the addition of *t*Bu_3_P–Pd-G2 (6 mg, 0.011 mmol, 4.0 mol %). The vial was sealed with a cap lined with a disposable Teflon septum, and the reaction was stirred at 60 °C for 18 h. At the end of the reaction, the mixture was quenched with distilled water (2.5 mL), and the aqueous layer was extracted with EtOAc (3 × 2.5 mL). The combined organic layers were dried over anhydrous sodium sulfate, filtered, and evaporated under reduced pressure. The remaining residue was purified by flash column chromatography on silica gel (1% EtOAc/hexane) to afford the corresponding coupled product **5** (48 mg, 0.23 mmol, 81%) as a yellow oil. ^1^H NMR (500 MHz, CDCl_3_): δ (ppm) 8.16 (br s, NH), 7.65 (dd, *J* = 5.3, 3.9 Hz, 1H), 7.43 (dd, *J* = 6.6, 1.2 Hz, 1H), 7.35 (dd, *J* = 6.6, 1.2 Hz, 1H), 7.29–7.27 (m, 2H), 7.15–7.13 (m, 1H), 6.74 (dd, *J* = 17.6, 11.0 Hz, 1H), 6.42 (ap t, *J* = 6.6, Hz, 1H), 6.31 (dd, *J* = 19.8, 13.9 Hz, 1H), 5.71–5.63 (m, 3H), 5.11 (dd, *J* = 11.0, 1.4 Hz, 1H). ^13^C{^1^H} NMR (125 MHz, CDCl_3_): δ (ppm) 144.1 (C), 143.8 (CH), 143.7 (C**), 136.5 (CH), 135.2 (C), 133.1 (CH), 130.0 (CH), 128.3 (CH_2_), 127.9 (CH*), 127.5 (CH), 126.2 (CH), 125.0 (CH), 113.5 (CH_2_), 110.5 (CH). *Carbon not observed in ^13^C{^1^H} NMR, assigned by gHSQC. **Carbon not observed in ^13^C{^1^H} NMR, assigned by gHMBC. ^11^B{^1^H} NMR (160 MHz, CDCl_3_): δ (ppm) 31.60. HRMS (EI-TOF) calculated for C_14_H_14_BN [M]^+^: 207.1228. Found [M]^+^: 207.1219.

#### 1,10a-Dihydro-1-aza-10a-boraphenanthrene (**1**)

The ruthenium catalyst Grubbs Second Generation G-II (30 mg, 0.035 mmol, 10 mol %) in CH_2_Cl_2_ (0.70 mL, 0.05 M) was added to a solution of the diene **5** (72 mg, 0.35 mmol, 1.0 equiv) in CH_2_Cl_2_ (3.5 mL, 0.1 M) under argon. The reaction mixture was heated at reflux for 24 h. The crude product was cooled to room temperature, diluted with dichloromethane (8 mL), and filtered through a pad of silica gel. The filtrate was concentrated *in vacuo*, and the remaining residue was purified by flash column chromatography on silica gel (5% EtOAc/Hex) to give the corresponding 1,10a-dihydro-1-aza-10a-boraphenantrene **1** (61 mg, 0.34 mmol, 98%) as a brown solid. Mp: 80–82 °C. ^1^H NMR (500 MHz, CDCl_3_): δ (ppm) 8.98 (d, *J* = 7.2 Hz, 1H), 8.79 (br s, NH), 8.54 (dd, *J* = 8.1, 1.1 Hz, 1H), 8.12 (d, *J* = 11.7 Hz, 1H), 7.78 (dd, *J* = 7.6, 1.1 Hz, 1H), 7.73 (ddd, *J* = 7.1, 6.2, 1.1 Hz, 1H), 7.56 (ap dt, *J* = 7.6, 1.1 Hz, 1H), 7.47 (ap dt, *J* = 7.6, 1.1 Hz, 1H), 7.10 (d, *J* = 11.7 Hz, 1H), 6.87 (ddd, *J* = 7.2, 6.2, 1.7 Hz, 1H). ^13^C{^1^H} NMR (125 MHz, CDCl_3_): δ (ppm) 145.5 (CH), 137.9 (CH), 134.8 (CH), 134.5 (C), 134.2 (C**), 134.1 (C), 130.8 (CH), 126.8 (CH*), 126.5 (CH), 125.5 (CH), 121.6 (CH), 111.2 (CH). *Carbon not observed in ^13^C{^1^H} NMR, assigned by gHSQC. **Carbon not observed in ^13^C{^1^H} NMR, assigned by gHMBC. ^11^B{^1^H} NMR (160 MHz, CDCl_3_): δ (ppm) 27.89. HRMS (APCI) calcd for C_12_H_10_BN [M + H]^+^: 179.1015. Found [M + H]^+^: 179.1011.

#### 3-Bromo-2-vinyl-1,2-dihydro-1-aza-2-boranaphthalene (**7**)

2-Vinylaniline **6** (402 mg, 3.38 mmol, 1.0 equiv) was dissolved in anhydrous toluene (16.9 mL, 0.02 M) in a Schlenk flask. Boron trichloride solution (1.0 M in hexanes; 6.75 mL, 6.75 mmol, 2.0 equiv) was added dropwise via syringe to the vigorously stirring solution of amine in toluene. At the conclusion of the addition, the reaction mixture was heated at reflux for 3 h. At the end of the reaction, volatiles were removed under reduced pressure to afford the corresponding B–Cl intermediate 2-chloro-1-aza-2-boranaphthalene as an air- and moisture-sensitive oil, which could be used as is in the next step without further purification. To the Schlenk containing the 2-chloro-2,1-borazaronaphthalene was added anhydrous CH_2_Cl_2_ (16.9 mL, 0.2 M), and the resulting solution was cooled to −30 °C. A recently prepared bromine solution (173 μL, 3.38 mmol, 1.0 equiv) in anhydrous CH_2_Cl_2_ (16.9 mL, 0.2 M) was added under argon at a rate of 1.1 mmol/h. After the addition, the reaction mixture was slowly warmed to −10 °C for an hour and a half, and the mixture was concentrated under reduced pressure to afford the corresponding intermediate 3-bromo-2-chloro-1-aza-2-boranaphthalene as an air- and moisture-sensitive oil, which could be used as is in the next step without further purification. To the Schlenk containing the 3-bromo-2-chloro-1-aza-2-boranaphthalene was added anhydrous THF (16.9 mL, 0.2 M), and the resulting solution was cooled to −30 °C. The vinylmagnesium bromide solution (1.0 M in Et_2_O; 6.75 mL, 6.75 mmol 2.0 equiv) was added dropwise using a syringe, and then the reaction mixture was allowed to warm to room temperature and stirred for 18 h. At the end of the reaction, the mixture was concentrated under reduced pressure, and the remaining residue was purified by flash column chromatography (1% EtOAc/hexane) to afford the corresponding 3-bromo-2-vinyl-1,2-dihydro-1-aza-2-boranaphthalene **7** (301 mg, 1.29 mmol, 38%) as a brown oil. ^1^H NMR (500 MHz, CDCl_3_): δ (ppm) 8.30 (s, 1H), 7.91 (br s, NH), 7.55 (d, *J* = 9.1 Hz, 1H), 7.44 (ap t, *J* = 8.0 Hz, 1H), 7.27 (d, *J* = 8.0 Hz, 1H), 7.21–7.17 (m, 1H), 6.68 (dd, *J* = 20.0, 13.9 Hz, 1H), 6.17 (dd, *J* = 20.0, 3.0 Hz, 1H), 6.03 (dd, *J* = 13.9, 3.0 Hz, 1H). ^13^C{^1^H} NMR (125 MHz, CDCl_3_): δ (ppm) 145.9 (CH), 139.1 (C), 131.4 (CH*), 131.4 (CH_2_), 128.9 (CH), 128.8 (CH), 127.9 (C**), 125.3 (C), 121.9 (CH), 118.2 (CH). *Carbon not observed in ^13^C{^1^H} NMR, assigned by gHSQC. **Carbon not observed in ^13^C{^1^H} NMR, assigned by gHMBC. ^11^B{^1^H} NMR (160 MHz, CDCl_3_): δ (ppm) 31.52. HRMS (EI-TOF) calculated for C_10_H_9_BBrN [M]^+^: 233.0012. Found [M]^+^: 233.0011.

#### 3-(2-Vinylphenyl)-2-vinyl-1,2-dihydro-1-aza-2-boranaphthalene (**8**)

In an oven-dried Biotage microwave vial equipped with a stir bar the 3-bromo-2-vinyl-2,1-borazaronaphthalene **7** (134 mg, 0.58 mmol, 1.0 equiv) and 2-vinylphenylboronic acid (110 mg, 0.75 mmol, 1.3 equiv) were dissolved in dioxane (2.87 mL). The resulting solution was treated with a suspension of cesium carbonate (562 mg, 1.73 mmol, 3.0 equiv) in distilled water (2.87 mL), before addition of *t*Bu_3_P–Pd-G2 (11.8 mg, 0.023 mmol, 4.0 mol %). The vial was sealed with a cap lined with a disposable Teflon septum, and the reaction was stirred at 60 °C for 18 h. At the end of the reaction, the mixture was quenched with distilled water (5 mL), and the aqueous layer was extracted with EtOAc (3 × 5 mL). The combined organic layers were dried over anhydrous sodium sulfate, filtered, and evaporated under reduced pressure. The remaining residue was purified by flash column chromatography on silica gel (hexane) to afford the corresponding coupled product **8** (114 mg, 0.44 mmol, 77%) as a brown oil. ^1^H NMR (500 MHz, CDCl_3_): δ (ppm) 7.99 (br s, NH), 7.78 (s, 1H), 7.66–7.62 (m, 2H), 7.45 (ap t, *J* = 8.3 Hz, 1H), 7.34–7.28 (m, 3H), 7.21–7.17 (m, 2H), 6.72 (dd, *J* = 17.6, 11.0 Hz, 1H), 6.34 (m, 1H), 5.81–5.73 (m, 2H), 5.65 (dd, *J* = 17.6, 1.4 Hz, 1H), 5.09 (dd, *J* = 11.0, 1.4 Hz, 1H). ^13^C{^1^H} NMR (125 MHz, CDCl_3_): δ (ppm) 143.9 (CH), 143.4 (C), 139.5 (C), 137.5 (C**), 136.2 (CH), 135.4 (C), 130.4 (CH*), 130.3 (CH_2_), 129.8 (CH), 129.6 (CH), 128.5 (CH), 127.6 (CH), 126.6 (CH), 125.2 (C), 125.1 (CH), 121.4 (CH), 117.9 (CH), 114.0 (CH_2_). *Carbon not observed in ^13^C{^1^H} NMR, assigned by gHSQC. **Carbon not observed in ^13^C{^1^H} NMR, assigned by gHMBC. ^11^B{^1^H} NMR (160 MHz, CDCl_3_): δ (ppm) 31.98. HRMS (APCI) calcd for C_18_H_17_BN [M + H]^+^: 258.1452. Found [M + H]^+^: 258,1451.

#### 6a,7-Dihydro-7-aza-6a-boratetraphene (**2**)

The ruthenium catalyst Grubbs Second Generation G-II (37 mg, 0.044 mmol, 10 mol %) in CH_2_Cl_2_ (0.88 mL, 0.05 M) was added to a solution of the diene **8** (114 mg, 0.44 mmol, 1.0 equiv) in CH_2_Cl_2_ (4.4 mL, 0.1 M) under argon. The reaction mixture was heated at reflux for 24 h. The crude product was cooled to room temperature, diluted with dichloromethane (10 mL), and filtered through a pad of silica gel. The filtrate was concentrated *in vacuo*, and the remaining residue was purified by flash column chromatography on silica gel (2% EtOAc/Hex) to give the corresponding 6a,7-dihydro-7-aza-6a-boratetraphene **2** (94 mg, 0.41 mmol, 93%) as a white solid. Mp: 130–132 °C. ^1^H NMR (500 MHz, CDCl_3_): δ (ppm) 9.19 (s, 1H), 8.56 (d, *J* = 7.9 Hz, 1H), 8.46 (br s, NH), 8.03 (d, *J* = 11.9 Hz, 1H), 7.99 (dd, *J* = 8.0, 0.8 Hz, 1H), 7.68 (dd, *J* = 7.7, 1.5 Hz, 1H), 7.60–7.53 (m, 2H), 7.51–7.46 (m, 2H), 7.34 (ddd, *J* = 8.0, 6.9, 1.2 Hz, 1H), 7.01 (d, *J* = 11.9 Hz, 1H). ^13^C{^1^H} NMR (125 MHz, CDCl_3_): δ (ppm) 147.9 (CH), 139.8 (C), 138.3 (CH), 134.8 (C), 134.2 (C), 134.0 (C**), 130.9 (CH), 130.7 (CH), 129.0 (CH), 127.1 (CH), 127.1 (CH*), 126.7 (CH), 125.1 (C), 122.2 (CH), 121.2 (CH), 118.5 (CH). *Carbon not observed in ^13^C{^1^H} NMR, assigned by gHSQC. **Carbon not observed in ^13^C{^1^H} NMR, assigned by gHMBC. ^11^B{^1^H} NMR (160 MHz, CDCl_3_): δ (ppm) 28.74. HRMS (APCI) calcd for C_16_H_13_BN [M + H]^+^: 230.1138. Found [M + H]^+^: 230.1138.

#### 10-Bromo-1,10a-dihydro-1-aza-10a-boraphenanthrene (**9**)

A mixture of AlCl_3_ (69 mg, 0.51 mmol, 1.5 equiv) and *N*-bromosuccinimide (NBS) (90 mg, 0.51 mmol, 1.5 equiv) was loaded in a Schlenk flask under argon. Dichloromethane (12 mL) was added, and the mixture was stirred at 25 °C for 30 min and then cooled to −35 °C. The resulting solution was treated with a solution of **1** (60 mg, 0.33 mmol, 1.0 equiv) in 12 mL of dichloromethane, and the reaction mixture was allowed to warm to room temperature over 6 h. At the end of the reaction, a saturated sodium thiosulfate solution (20 mL) was added, and the aqueous layer was extracted with dichloromethane (3 × 20 mL). The combined organic layers were dried over Na_2_SO_4_, filtered, and concentrated to dryness. Purification of the resulting residue by flash column chromatography on silica gel (hexanes) afforded the product **9** as a yellow pale solid (56.0 mg, 0.22 mmol, 66%). Mp: 133–135 °C. ^1^H NMR (500 MHz, CDCl_3_): δ (ppm) 9.12 (br s, NH), 8.94 (dd, *J* = 7.4, 1.0 Hz, 1H), 8.44 (dd, *J* = 8.2, 1.2 Hz, 1H), 8.27 (s, 1H), 7.85 (ddd, *J* = 7.4, 6.2, 1.1 Hz, 1H), 7.66 (dd, *J* = 7.8, 1.4 Hz, 1H), 7.54 (ddd, *J* = 8.2, 7.1, 1.4 Hz, 1H), 7.43 (ddd, *J* = 7.8, 7.1, 1.2 Hz, 1H), 6.94 (ddd, *J* = 7.4, 6.2, 1.7 Hz, 1H). ^13^C{^1^H} NMR (125 MHz, CDCl_3_): δ (ppm) 144.9 (CH), 138.9 (CH), 135.5 (CH), 134.5 (C**), 134.1 (C), 133.9 (C), 130.2 (CH), 126.9 (CH), 126.1 (CH), 125.3 (C**), 121.9 (CH), 112.4 (CH). **Carbon not observed in ^13^C{^1^H} NMR, assigned by gHMBC. ^11^B{^1^H} NMR (160 MHz, CDCl_3_): δ (ppm) 26.62. HRMS (EI-TOF) calculated for C_12_H_9_BBrN [M]^+^: 257.0015. Found [M]^+^: 257.0011.

#### 6-Bromo-6a,7-dihydro-7-aza-6a-boratetraphene (**10**)

A mixture of AlCl_3_ (131.0 mg, 0.98 mmol, 1.5 equiv) and *N*-bromosuccinimide (NBS) (175.0 mg, 0.98 mmol, 1.5 equiv) was loaded in a Schlenk flask under argon. Dichloromethane (16 mL) was added, and the mixture was stirred at 25 °C for 30 min and then cooled to −35 °C. The resulting solution was treated with a solution of **2** (150.0 mg, 0.66 mmol, 1.0 equiv) in 16 mL of dichloromethane, and the reaction mixture was allowed to warm to room temperature over 6 h. At the end of the reaction, a saturated sodium thiosulfate solution (30 mL) was added, and the aqueous layer was extracted with dichloromethane (3 × 30 mL). The combined organic layers were dried over Na_2_SO_4_, filtered, and concentrated to dryness. Purification of the resulting residue by flash column chromatography on silica gel (hexanes) afforded the product **10** as a yellow pale solid (143.0 mg, 0.46 mmol, 71%). Mp: 159–161 °C. ^1^H NMR (500 MHz, CDCl_3_): δ (ppm) 9.11 (s, 1H), 8.64 (br s, NH), 8.45 (d, *J* = 7.9 Hz, 1H), 8.16 (s, 1H), 7.96 (d, *J* = 8.0 Hz, 1H), 7.62 (ddd, *J* = 8.0; 6.8; 1.3 Hz, 1H), 7.58 (d, *J* = 8.0 Hz, 1H), 7.56–7.53 (m, 2H), 7.45–7.42 (m, 1H), 7.37 (ddd, *J* = 8.0; 6.8; 1.3 Hz, 1H). ^13^C{^1^H} NMR (125 MHz, CDCl_3_): δ (ppm) 147.0 (CH), 139.6 (C), 139.4 (CH), 134.5 (C), 133.5 (C), 130.7 (CH), 130.4 (CH), 130.3 (C**), 129.6 (CH), 127.5 (CH), 127.0 (CH), 125.7 (C**), 125.5 (C), 122.5 (CH), 121.8 (CH), 118.8 (CH). **Carbon not observed in ^13^C{^1^H} NMR, assigned by gHMBC. ^11^B{^1^H} NMR (160 MHz, CDCl_3_): δ (ppm) 26.96. HRMS (EI-TOF) calculated for C_16_H_11_BBrN [M]^+^: 307.0158. Found [M]^+^: 307.0168.

#### 10-Phenyl-1,10a-dihydro-1-aza-10a-boraphenanthrene (**11**)

In a round-bottom flask equipped with a stir bar, the brominated BN-phenantrene **9** (20.0 mg, 0.08 mmol, 1.0 equiv) and phenylboronic acid (27.0 mg, 0.22 mmol, 2.8 equiv) were dissolved in 0.32 mL of toluene and 0.08 mL of methanol and treated with a suspension of Na_2_CO_3_ (190.0 mg) in 0.76 mL of water. Then Pd(PPh_3_)_4_ (4.5 mg, 0.004 mmol, 5 mol %) was added, and the mixture was heated to 70 °C and stirred overnight. After the addition of water (3.5 mL) and extraction with dichloromethane (3 × 3.5 mL), the combined organic layers were dried over Na_2_SO_4_, filtered, and concentrated under a vacuum. The crude organic product was purified by flash column chromatography on silica gel (5% AcOEt/hexane) to give **11** as a white solid (17.0 mg, 0.07 mmol, 85%). Mp: 152–154 °C. ^1^H NMR (500 MHz, CDCl_3_): δ (ppm) 9.02 (dd, *J* = 7.3, 1.1 Hz, 1H), 9.02 (br s, NH), 8.53 (ddd, *J* = 8.0, 1.3, 0.6 Hz, 1H), 7.98 (s, 1H), 7.83–7.78 (m, 2H), 7.58–7.51 (m, 5H), 7.48 (ddd, *J* = 7.7, 7.1, 1.3 Hz, 1H), 7.41–7.37 (m, 1H), 6.91 (ddd, *J* = 7.3, 6.2, 1.6 Hz, 1H). ^13^C{^1^H} NMR (125 MHz, CDCl_3_): δ (ppm) 144.0 (C), 142.2 (CH), 140.9 (C**), 138.2 (CH), 135.0 (CH), 134.9 (C**), 134.2 (C), 133.8 (C), 131.1 (CH), 129.2 (2CH), 128.3 (2CH), 126.6 (CH), 126.4 (CH), 125.8 (CH), 121.5 (CH), 111.4 (CH). **Carbon not observed in ^13^C{^1^H} NMR, assigned by gHMBC. ^11^B{^1^H} NMR (160 MHz, CDCl_3_): δ (ppm) 27.49. HRMS (APCI) calcd for C_18_H_14_BN [M + H]^+^: 256.1295. Found [M + H]^+^: 256.1289.

#### 10-(Phenylethynyl)-1,10a-dihydro-1-aza-10a-boraphenanthrene (**12**)

To an oven-dried Schlenk flask charged with **9** (20.0 mg, 0.08 mmol, 1.0 equiv), phenylacetylene (26 μL, 0.24 mmol, 3.0 equiv), Pd(PPh_3_)_2_Cl_2_ (2.8 mg, 0.004 mmol, 5 mol %), and CuI (0.6 mg, 0.004 mmol, 5 mol %) was added triethylamine (33 μL, 0.24 mmol, 3.0 equiv) and DMF (0.9 mL). The mixture was heated and stirred at 80 °C for 24 h. The resulting mixture was successively washed with water (5 mL) and extracted with dichloromethane (3 × 5 mL). The combined organic layers were dried over Na_2_SO_4_, filtered, and concentrated under a vacuum. The resulting product was purified by flash column chromatography on silica gel (hexanes/EtOAc 95:5) to give **12** as a brown oil (19.0 mg, 0.07 mmol, 88%). ^1^H NMR (500 MHz, CDCl_3_): δ (ppm) 9.25 (br s, NH), 8.97 (dd, *J* = 7.4, 1.0 Hz, 1H), 8.47 (dd, *J* = 8.2, 1.2 Hz, 1H), 8.28 (s, 1H), 7.90–7.86 (m, 1H), 7.75 (dd, *J* = 8.1, 1.4 Hz, 1H), 7.64–7.61 (m, 2H), 7.55 (ddd, *J* = 8.2, 7.1, 1.4 Hz, 1H), 7.45 (ddd, *J* = 8.4, 7.1, 1.2 Hz, 1H), 7.42–7.34 (m, 3H), 6.93 (ddd, *J* = 7.3, 6.1, 1.7 Hz, 1H). ^13^C{^1^H} NMR (125 MHz, CDCl_3_): δ (ppm) 147.8 (CH), 138.7 (CH), 135.3 (CH), 135.0 (C**), 134.6 (C), 133.4 (C), 131.7 (2CH), 131.1 (CH), 128.5 (2CH), 128.0 (CH), 127.4 (CH), 125.9 (CH), 124.4 (C), 121.6 (CH), 119.9 (C**), 112.0 (CH), 94.9 (C), 90.6 (C). **Carbon not observed in ^13^C{^1^H} NMR, assigned by gHMBC. ^11^B{^1^H} NMR (160 MHz, CDCl_3_): δ (ppm) 27.47. HRMS (APCI) calcd for C_20_H_14_BN [M + H]^+^: 279.1328. Found [M + H]^+^: 279.1316.

#### 10-(*N*-Morpholinyl)-1,10a-dihydro-1-aza-10a-boraphenanthrene (**13**)

To an oven-dried Biotage microwave vial equipped with a stir bar were added [PdCl(allyl)]_2_ (0.9 mg, 0.002 mmol, 2.5 mol %), JohnPhos (1.2 mg, 0.004 mmol, 5.0 mol %), and *t*-BuONa (10.6 mg, 0.11 mmol, 1.4 equiv). The vial was sealed with a cap lined with a disposable Teflon septum, evacuated under vacuum, and purged with argon three times. Toluene (0.25 mL) was added, followed by brominated BN-phenanthrene **9** (20.0 mg, 0.08 mmol, 1.0 equiv) and morpholine (9 μL, 0.10 mmol, 1.2 equiv). The resulting mixture was heated to 80 °C and stirred until full consumption of **9** was observed by TLC (24 h). The reaction mixture was cooled to room temperature, diluted with Et_2_O (5 mL), and filtered over Celite. The solvent was removed in vacuo, and the resulting product was purified by flash column chromatography on silica gel (hexanes/EtOAc 8:2). The product **13** was obtained as yellow solid (13 mg, 0.05 mmol, 62%). Mp: 197–199 °C. ^1^H NMR (500 MHz, CDCl_3_): δ (ppm) 8.96 (br s, NH), 8.87 (dd, *J* = 7.6, 1.1 Hz, 1H), 8.36–8.34 (m, 1H), 7.74 (ddd, *J* = 7.6, 6.2, 1.1 Hz, 1H), 7.60–7.58 (m, 1H), 7.40–7.21 (m, 2H), 7.21 (s, 1H), 6.85 (ddd, *J* = 7.6, 6.2, 1.1 Hz, 1H), 4.00 (ap t, *J* = 4.6 Hz, 4H), 3.20 (ap t, *J* = 4.6 Hz, 4H). ^13^C{^1^H} NMR (125 MHz, CDCl_3_): δ (ppm) 137.9 (CH), 135.8 (C**) 134,4 (CH), 134,1 (C**), 133.6 (CH), 131.9 (C), 129.5 (CH), 125.9 (CH), 125.1 (CH), 124.8 (CH), 121.3 (CH), 111.4 (CH), 67.4 (2CH_2_), 53.1 (2CH_2_).**Carbon not observed in ^13^C{^1^H} NMR, assigned by gHMBC. ^11^B{^1^H} NMR (160 MHz, CDCl_3_): δ (ppm) 26.93. HRMS (APCI) calcd for C_16_H_17_BN_2_O [M + H] ^+^: 264.1543. Found [M + H]^+^: 264.1549.

#### 6-Phenyl-7-aza-6a-boratetraphene (**14**)

In a round-bottom flask equipped with a stir bar, the brominated BN-tetraphene **10** (30.0 mg, 0.10 mmol, 1.0 equiv) and phenylboronic acid (33.0 mg, 0.27 mmol, 2.8 equiv) were dissolved in 0.40 mL of toluene and 0.10 mL of methanol and treated with a suspension of Na_2_CO_3_ (238.0 mg) in 1.0 mL of water. Then Pd(PPh_3_)_4_ (5.6 mg, 0.005 mmol, 5 mol %) was added, and the mixture was heated to 70 °C and stirred overnight. After the addition of water (4 mL) and extraction with dichloromethane (3 × 4 mL), the combined organic layers were dried over Na_2_SO_4_, filtered, and concentrated under a vacuum. The crude organic product was purified by flash column chromatography on silica gel (1% AcOEt/hexane) to give **14** as a white solid (29.0 mg, 0.10 mmol, 97%). Mp: 131–133 °C. ^1^H NMR (500 MHz, CDCl_3_): δ (ppm) 9.24 (s, 1H), 8.59 (br s, NH), 8.57 (d, *J* = 7.8 Hz, 1H), 8.01 (d, *J* = 7.7 Hz, 1H), 7.90 (s, 1H), 7.74 (d, *J* = 7.4 Hz, 1H), 7.62–7.55 (m, 6H), 7.50–7.47 (m, 2H), 7.43 (ap t, *J* = 7.8 Hz, 1H), 7.35 (ap t, *J* = 7.7 Hz, 1H). ^13^C{^1^H} NMR (125 MHz, CDCl_3_): δ (ppm) 144.4 (CH), 143.8 (C), 141.0 (C**), 139.7 (C), 138.6 (CH), 134.6 (C), 133.9 (C), 131.2 (CH), 130.6 (CH), 129.3 (2CH), 129.0 (CH), 128.1 (2CH), 127.1 (CH), 126.9 (CH), 126.7 (CH), 125.1 (C), 122.1 (CH), 121.4 (CH), 118.7 (CH). **Carbon not observed in ^13^C{^1^H} NMR, assigned by gHMBC. ^11^B{^1^H} NMR (160 MHz, CDCl_3_): δ (ppm) 28.28. HRMS (APCI) calcd for C_22_H_17_BN [M + H]^+^: 306.1453. Found [M + H]^+^: 306.1462.

#### 6-(Phenylethynyl)-7-aza-6a-boratetraphene (**15**)

To an oven-dried Schlenk flask charged with **10** (30.0 mg, 0.10 mmol, 1.0 equiv), phenylacetylene (32 μL, 0.29 mmol, 3.0 equiv), Pd(PPh_3_)_2_Cl_2_ (3.4 mg, 0.005 mmol, 5 mol %), and CuI (0.9 mg, 0.005 mmol, 5 mol %) was added triethylamine (41 μL, 0.29 mmol, 3.0 equiv) and DMF (1.0 mL). The mixture was heated and stirred at 80 °C for 24 h. The resulting mixture was successively washed with water (5 mL) and extracted with dichloromethane (3 × 5 mL). The combined organic layers were dried over Na_2_SO_4_, filtered, and concentrated under a vacuum. The resulting product was purified by flash column chromatography on silica gel (hexanes) to give **15** as a pale yellow solid (25.0 mg, 0.08 mmol, 78%). Mp: 156–158 °C. ^1^H NMR (500 MHz, CDCl_3_): δ (ppm) 9.19 (s, 1H), 8.78 (br s, NH), 8.52 (d, *J* = 7.9 Hz, 1H), 8.21 (s, 1H), 8.01 (d, *J* = 8.1 Hz, 1H), 7.69–7.66 (m, 3H), 7.63–7.62 (m, 2H), 7.57–7.54 (m, 1H), 7.48–7.36 (m, 5H). ^13^C{^1^H} NMR (125 MHz, CDCl_3_): δ (ppm) 149.9 (CH), 139.6 (C), 139.2 (CH), 134.1 (C), 134.1 (C), 132.8 (C**), 131.8 (2CH), 131.2 (CH), 130.7 (CH), 129.4 (CH), 128.6 (2CH), 128.2 (CH), 127.9 (CH), 126.9 (CH), 125.4 (C), 124.3 (C), 122.3 (CH), 121.5 (CH), 120,0 (C**), 118.8 (CH), 95.2 (C), 90.4 (C). **Carbon not observed in ^13^C{^1^H} NMR, assigned by gHMBC. ^11^B{^1^H} NMR (160 MHz, CDCl_3_): δ (ppm) 28.06. HRMS (APCI) calcd for C_24_H_17_BN [M + H]^+^: 330.1453. Found [M + H]^+^: 330.1461.

#### 6-(*N*-Morpholinyl)-7-aza-6a-boratetraphene (**16**)

To an oven-dried Biotage microwave vial equipped with a stir bar were added [PdCl(allyl)]_2_ (0.9 mg, 0.002 mmol, 2.5 mol %), JohnPhos (1.4 mg, 0.005 mmol, 5.0 mol %), and *t*-BuONa (13 mg, 0.14 mmol, 1.4 equiv). The vial was sealed with a cap lined with a disposable Teflon septum, evacuated under vacuum, and purged with argon three times. Toluene (0.30 mL) was added, followed by brominated BN-tetraphene **1** (30.0 mg, 0.10 mmol, 1.0 equiv) and morpholine (10 μL, 0.12 mmol, 1.2 equiv). The resulting mixture was heated to 80 °C and stirred until full consumption of **1** was observed by TLC (24 h). The reaction mixture was cooled to room temperature, diluted with Et_2_O (5 mL), and filtered over Celite. The solvent was removed in vacuo, and the resulting product was purified by flash column chromatography on silica gel (hexanes/EtOAc 9:1). The product **16** was obtained as a yellow solid (16 mg, 0.05 mmol, 50%). Mp: 138–140 °C. ^1^H NMR (500 MHz, CDCl_3_): δ (ppm) 9.10 (s, 1H), 8.44 (br s, NH), 8.42–8.40 (m, 1H), 7.96 (dd, *J* = 7.9; 1.3 Hz, 1H), 7.59–7.56 (m, 1H), 7.54–7.52 (m, 2H), 7.40–7.38 (m, 2H), 7.36–7.33 (m, 1H), 7.12 (s, 1H), 4.03–4.01 (m, 4H), 3.21–3.19 (m, 4H). ^13^C{^1^H} NMR (125 MHz, CDCl_3_): δ (ppm) 152.4 (C**), 138.9 (C), 138.3 (CH), 135.4 (C), 134.1 (C**), 131.7 (C), 130.5 (CH), 129.6 (CH), 129.0 (CH), 127.1 (CH), 126.3 (CH), 125.3 (CH), 125.1 (C), 122.0 (CH), 121.5 (CH), 118.6 (CH), 67.4 (2CH_2_), 52.7 (2CH_2_). **Carbon not observed in ^13^C{^1^H} NMR, assigned by gHMBC. ^11^B{^1^H} NMR (160 MHz, CDCl_3_): δ (ppm) 27.51. HRMS (APCI) calcd for C_20_H_20_BN_2_O [M + H] ^+^: 315.1667. Found [M + H]^+^: 315.1675.

#### 7-(Methyl)aza-6a-boratetraphene (**17**)

To a 4 mL reaction vial equipped with a stir bar was added 7-aza-6a-boratetraphene **2** (20 mg, 0.09 mmol, 1 equiv) followed by THF (0.18 mL). The vial was sealed and placed under an Ar atmosphere. LiHMDS (25 μL, 0.13 mmol, 1.5 equiv) was added dropwise via a syringe. The solution was stirred for 4 h at room temperature. After this time, the reaction mixture was cooled to 0 °C, and iodomethane (25 μL, 0.13 mmol, 1.5 equiv) was added. The reaction mixture was allowed to stir at 0 °C for 10 min, then warmed to room temperature. The solution was stirred at this temperature overnight. A second addition of LiHMDS (8 μL, 0.04 mmol, 0.5 equiv) and iodomethane (8 μL, 0.04 mmol, 0.5 equiv) were added at this time, and the resulting solution was stirred for 6 h at room temperature. The reaction was quenched with deionized H_2_O, and the aqueous layer was extracted with Et_2_O. The organic layer was dried (Na_2_SO_4_), filtered, and concentrated under a vacuum. The resulting product was purified by flash column chromatography on silica gel (hexanes) to give the desired product **17** as a white solid (11.0 mg, 0.05 mmol, 52%). Mp: 114–116 °C. ^1^H NMR (500 MHz, CDCl_3_): δ (ppm) 9.19 (s, 1H), 8.57 (d, *J* = 7.9 Hz, 1H), 8.07–80.2 (m, 2H), 7.80 (d, *J* = 8.6 Hz, 1H), 7.71–7.67 (m, 2H), 7.56–53 (m, 1H), 7.47–7.41 (m, 1H), 7.39 (ddd, *J* = 7.9, 7.0, 1.0 Hz, 1H), 7.24 (d, *J* = 12.2 Hz, 1H), 4.06 (s, 3H). ^13^C{^1^H} NMR (125 MHz, CDCl_3_): δ (ppm) 147.7 (CH), 141.8 (C), 138.5 (CH), 134.4 (C), 134.2 (C), 133.3 (C**), 131.5 (CH), 130.7 (CH), 129.4 (CH), 127.1 (CH), 127.1 (CH*), 126.6 (CH), 125.8 (C), 122.2 (CH), 120.8 (CH), 115.0 (CH), 35.2 (CH_3_). *Carbon not observed in ^13^C{^1^H} NMR, assigned by gHSQC. **Carbon not observed in ^13^C{^1^H} NMR, assigned by gHMBC. ^11^B{^1^H} NMR (160 MHz, CDCl_3_): δ (ppm) 29.51. HRMS (APCI) calcd for C_17_H_15_BN [M + H]^+^: 244.1295. Found [M + H]^+^: 244.1296.

## Supporting Information Available

Photophysical data, X-ray crystallographic data for **2**, and ^1^H, ^13^C{^1^H}, and ^11^B{^1^H} NMR spectra for new compounds (PDF)

## Accession Codes

CCDC 2073370 contains the supplementary crystallographic data for this paper. These data can be obtained free of charge via www.ccdc.cam.ac.uk/data_request/cif, or by emailing data_request@ccdc.cam.ac.uk, or by contacting The Cambridge Crystallographic Data Centre, 12 Union Road, Cambridge CB2 1EZ, UK; fax: +44 1223 336033.
