# Supplementary material for: 1,10a-Dihydro-1-aza-10a-boraphenanthrene and 6a,7-Dihydro-7-aza-6a-boratetraphene: Two New Fluorescent BN-PAHs
Source: J Org Chem. 2021 Nov 22;86(23):16259–67. doi: 10.1021/acs.joc.1c01095 (PMC8650019; doi:10.1021/acs.joc.1c01095)

1,10a-Dihydro-1-aza-10a-boraphenanthrene and 6a,7-dihydro-7-aza-6a-boratetraphene: two new fluorescent BN-PAHs

Isabel Valencia,[†] Patricia García-García,[†] David Sucunza,[*,†] Francisco Mendicuti[‡] and Juan J. Vaquero[*,†]

[†] Universidad de Alcalá, IRYCIS, Departamento de Química Orgánica y Química Inorgánica, Instituto de Investigación Química "Andrés M. del Río" (IQAR), Campus Científico-Tecnológico, 28805-Alcalá de Henares, Spain. Corresponding authors: david.sucunza@uah.es; juanjose.vaquero@uah.es.

[‡] Universidad de Alcalá, Departamento de Química Analítica, Química Física e Ingeniería Química, Instituto de Investigación Química "Andrés M. del Río" (IQAR), Campus Científico-Tecnológico, 28805-Alcalá de Henares, Spain.

Corresponding authors: david.sucunza@uah.es; juanjose.vaquero@uah.es.

Electronic Supplementary Information

Table of Contents

Photophysical data -- S2

Experimental evidence which confirms the presence of π-π *stacking* ground state aggregates of **13** BN-phenanthrene and **16** BN-tetraphene derivatives in solution--------------------------------S10

X-ray crystallographic data for **2** ---S14

Verification of fluoride titrations of BN-phenanthrene **1** by ^{19}F, ^{11}B and ^{10}B NMR measurements --S23

Copies of ^{1}H, ^{13}C and ^{11}B-NMR spectra for novel compounds and selected gCOSY, TOCSY, NOESY, gHSQC and gHMBC spectra---S25

PHOTOPHYSICAL DATA

Spectroscopic Measurements

Absorption spectra were recorded in a UV-Vis UVIKON 941 Spectrophotometer in the 250-500 nm range. Steady-state fluorescence measurements were performed by using a PTI spectrofluorimeter equipped with single monochromators in the excitation and emission paths. Polarizers were fixed at the magic angle conditions. Fluorescence decay measurements were carried out on a PTI time-correlated single-photon-counting (TCSPC) spectrometer upgraded to use Horiba Nanoleds. A Nanoled emitting at 335 nm was employed as the excitation source. Photons were detected by a sensitive cooled photomultiplier. The data acquisition was carried out by a multichannel analyzer (1024 channels), with a time window width of 125-200 ns. A total of 10,000 counts, in the maximum peak channel, was taken for each measurement. Instrumental response functions were regularly obtained by measuring the scattering of a Ludox solution. Intensity fluorescence profiles were fitted to the usual multi-exponential decay functions,

$$I(t) = \sum_{i=1}^{n} A_i e^{-t/\tau_i}$$

by using the iterative deconvolution method, under the assumption that each component behaves independently.[1] The average lifetime of a multiple-exponential decay function can be defined as,

$$\langle \tau \rangle = \frac{\sum_{i=1}^{n} A_i \tau_i^2}{\sum_{i=1}^{n} A_i \tau_i}$$

where A_i is the pre-exponential factor of the component with a lifetime τ_i of the multi-exponential function intensity decay.[2] Right angle geometry and rectangular 1.0 cm path cells were used for all the measurements.

Fluorescence Quenching[3]

Collisional or dynamic quenching of the fluorescence of a single chromophore is described by the Stern-Volmer equation:

$$\frac{F_0}{F} = \frac{\tau_0}{\tau} = 1 + K_D[Q] = 1 + k_q \tau_0 [Q]$$

where F_0 and F *(τ_o and τ)* are the chromophore fluorescence intensities (fluorescence lifetimes) in the absence and presence of a quencher; k_q is the bimolecular quenching constant; τ_0 is the

---

[1] O'Connor DV, Ware WR, Andre JC. Deconvolution of fluorescence decay curves. A critical comparison of techniques. J Phys Chem 1979;83(10):1333-43.

[2] Joseph R. Lakowicz, *Principles of Fluorescence Spectroscopy, 3rd edition* Springer-Verlag , Boston MA, pp 97-155, 2006.

[3] Joseph R. Lakowicz, *Principles of Fluorescence Spectroscopy, 3rd edition* Springer-Verlag , Boston MA, pp 277-330, 2006.

lifetime of the chromophore in the absence of Q. The Stern-Volmer quenching constant is given by $K_D = k_q \tau_0$.

Fluorescence quenching can occur as a result of the formation of a non-fluorescent ground-state complex between the chromophore and quencher (static quenching). In this case, the intensity (F) decreasing can also be fitted to a Stern-Volmer equation,

$$\frac{F_0}{F} = 1 + K_S[Q]$$

which is identical to that observed for dynamic quenching, although K_s is now the binding constant for the complex formation. However, static quenching does not open any new excited stated deactivation ways, therefore $\tau_0/\tau = 1$.

It is necessary to point out, that the binding constants obtained from Stern-Volmer plots are only valid when: (i) the complexed chromophores are non-fluorescent and when (ii) during the quenching experiment the initial quencher concentration [Q]>>[chromophore] and the complexed Q concentration ([Q-F]) can be neglected.

These two points are not fulfilled in our experiment as the fluoroborate complexes of **1** or **2** and F^- are also fluorescent and [TBAF] is of the same order as [**1**] or [**2**] during part of the titration.

It is a better option to get binding constants as described in the next section.

Thermodynamics of the 1 (or 2) to ligand binding [4]

For the 1:1 stoichiometry **1**:*L* complex formation, according to the following equilibrium:

$$\mathbf{1} + L \xrightleftharpoons{K} \mathbf{1}:L$$

the association constant of the 1:*L* complex (C) is defined as,

$$K = \frac{[C]}{[\mathbf{1}][L]} \qquad \text{(S1)}$$

Taking into account the corresponding mass balances:

$$[\mathbf{1}]_0 = [\mathbf{1}] + [C] \qquad \text{(S2)}$$

$$[L]_0 = [L] + [C] \qquad \text{(S3)}$$

where [*C*], [**1**] and [*L*] are the complex, free fluorescent compound and ligand (fluoride) concentrations respectively at the equilibrium and $[\mathbf{1}]_0$ and $[L]_0$ are the initial **1** derivative and ligand concentrations.
For higher order stoichiometries 1:*n* for the complex the total L concentration in solution is:

$$[L]_0 = [L] + n[C] \qquad \text{(S4)}$$

and the fraction complexed species can be related to the equilibrium constant by:

$$\frac{n[C]}{[\mathbf{1}]_0} = \frac{nK[L]}{1+K[L]} \qquad \text{(S5)}$$

By the substitution of [*L*] from previous equations, the [*C*] concentration is:

$$[C] = \frac{\left\{1+K[L]_0+nK[\mathbf{1}]_0\right\} \pm \sqrt{\left\{1+K[L]_0+nK[\mathbf{1}]_0\right\}^2 - 4nK^2[L]_0[\mathbf{1}]_0}}{2nK} \qquad \text{(S6)}$$

The free [**1**] can be determined as:

$$[\mathbf{1}] = \frac{\{nK[\mathbf{1}]_0 - K[L]_0 - 1\} \pm \sqrt{\{1 + K[L]_0 + nK[\mathbf{1}]_0\}^2 - 4nK^2[L]_0[\mathbf{1}]_0}}{2nK} \quad \text{(S7)}$$

The total fluorescence intensity (*I*) due to the fluorescents **1** and complex is

$$I = x_1 I_1 + x_C I_C = \frac{[\mathbf{1}]}{[\mathbf{1}]_0} I_1 + \frac{[C]}{[\mathbf{1}]_0} I_C \quad \text{or} \quad I = \frac{[\mathbf{1}]}{[\mathbf{1}]_0} I_0 + \frac{[\mathbf{1}]}{[\mathbf{1}]_0} I_\infty \quad \text{(S8)}$$

Substracting I_0 gives,

$$\Delta I = I - I_0 = I_\infty - I_0\left(1 - \frac{[\mathbf{1}]}{[\mathbf{1}]_0}\right) \quad \text{(S9)}$$

or,

$$\Delta I = (I_\infty - I_0)\frac{[C]}{[\mathbf{1}]_0} \quad \text{(S10)}$$

where I_0 is the fluorescence intensity for the uncomplexed **1**, I_∞ is the fluorescence intensity of the *C* complex, $[\mathbf{1}]_0$ the total concentration of fluorescent **1** and *K* the association constant of the host-guest system.

The substitution of [*C*] from S6 into S10 results in,

$$\Delta I = (I_\infty - I_0)\frac{\{1/[\mathbf{1}]_0 + K[L]_0/[\mathbf{1}]_0 + nK\} \pm \sqrt{\{1 + K[L]_0/[\mathbf{1}]_0 + nK\}^2 - 4nK^2[L]_0/[\mathbf{1}]_0}}{2nK} \quad \text{(S11)}$$

(S11) can be modified as:

$$\frac{\Delta \mathrm{I}}{\mathrm{I}_0} = \left(\frac{\mathrm{I}_\infty - \mathrm{I}_0}{\mathrm{I}_0}\right)\frac{\{1/[\mathbf{1}]_0 + KR + nK\} \pm \sqrt{\{1 + KR + nK\}^2 - 4nK^2R}}{2nK} \quad \text{(S12)}$$

where *R* is the $[\mathbf{L}]_0/[\mathbf{1}]_0$ molar ratio or equivalents of L added during titration.

Equations S11 and S12 are valid for titrations of fluorescent derivatives and both, fluorescent complexes or non-fluorescent ones, *i.e.*, the fluorescence decreases or increases upon titration. In addition, these equations are valid for any **L** (or quencher) concentration.

---

[4] Bosch P, García V, Bilen BS, Sucunza D, Domingo A, Mendicuti F, Vaquero JJ. Imidazopyridinium cations: A new family of azonia aromatic heterocycles with applications as DNA intercalators, Dyes & Pigments, 2017; 138: 135-146.

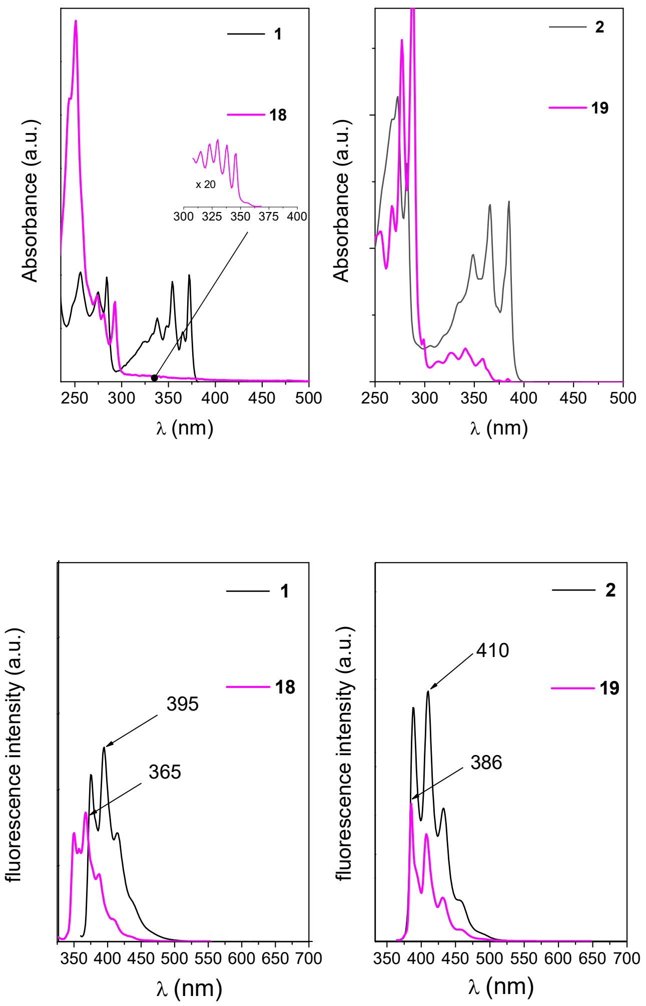

Figure S1. UV/Vis absorption and emission spectra for **1** BN-phenanthrene and **2** BN-tetraphene, as well as their phenanthrene (**18**) and tetraphene (**19**) PAH isostere derivatives in dilute solutions of cyclohexane at 25ºC.

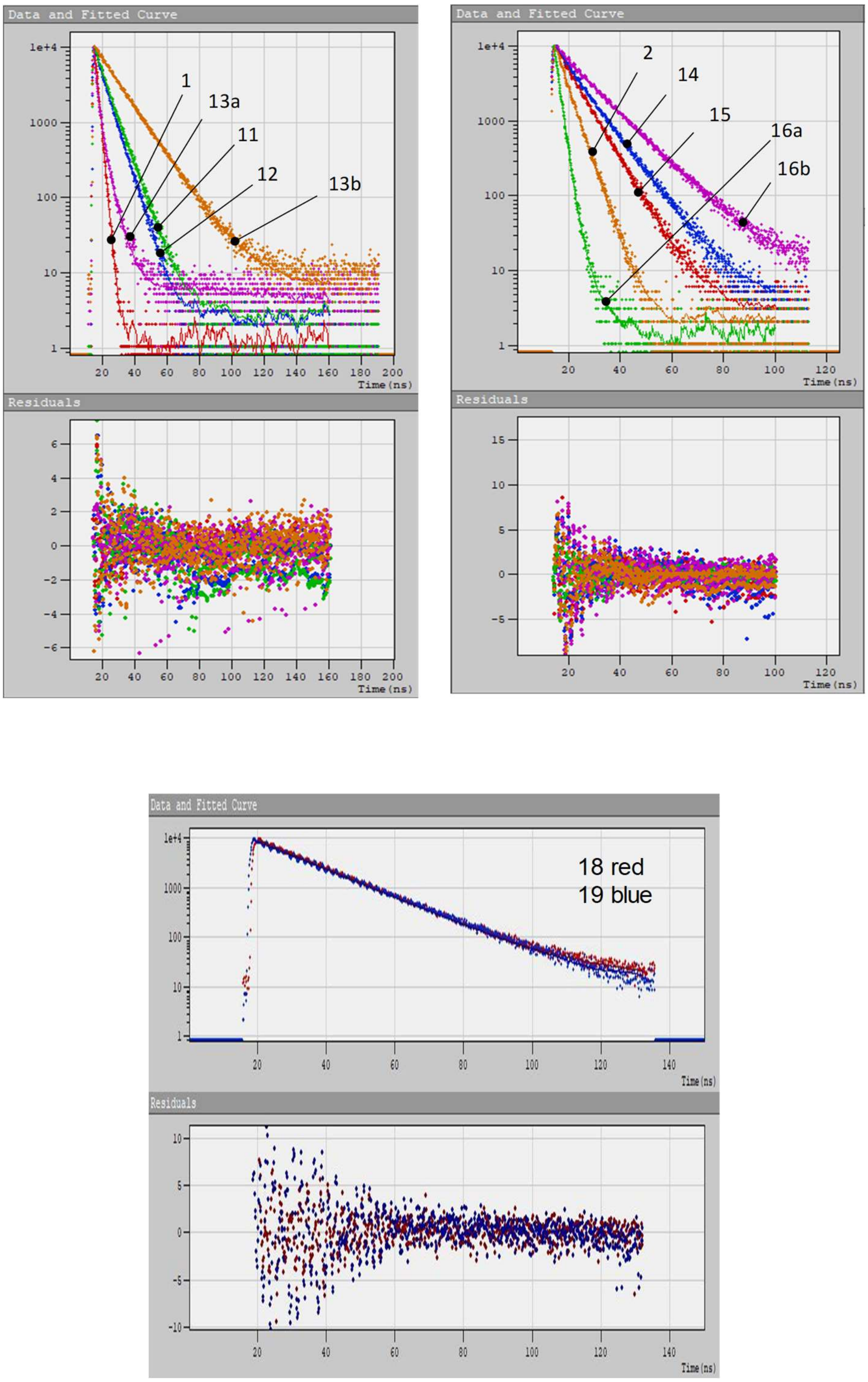

Figure S2. Fluorescence intensity decay profiles (left) **1**, **11-13** BN-phenanthrene and (right) **2**, **14-16** BN-tetraphene derivatives, as well as phenanthrene (**18**) and tetraphene (**19**) in cyclohexane at 25ºC. λ_{ex} = 335 nm (λ_{ex} = 296 nm for **18**) was fixed. For **13** and **16**, two λ_{em} were selected (Table 1).

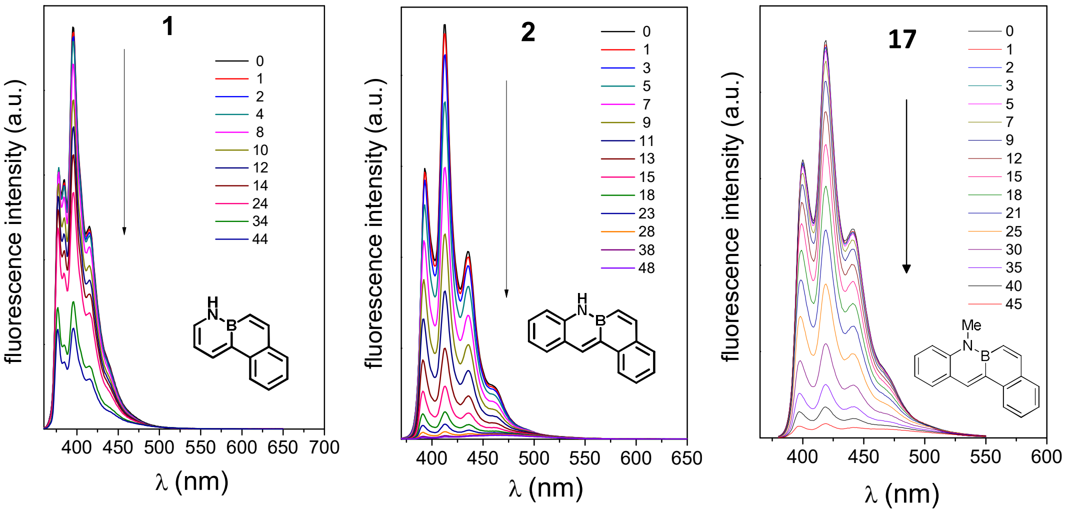

Figure S3. Titrations with TBAF in THF at 25ºC of the **1** BN-phenanthrene, **2** BN-tetraphene derivatives and the methylated BN-tetraphene one (**17**). ([**1**]=6.72x10^{-5} M, [**2**]=4.56x10^{-5} M and [**17**]=8.22x10^{-5} M; λ_{ex} =354 nm, 365 and 373 nm respectively).

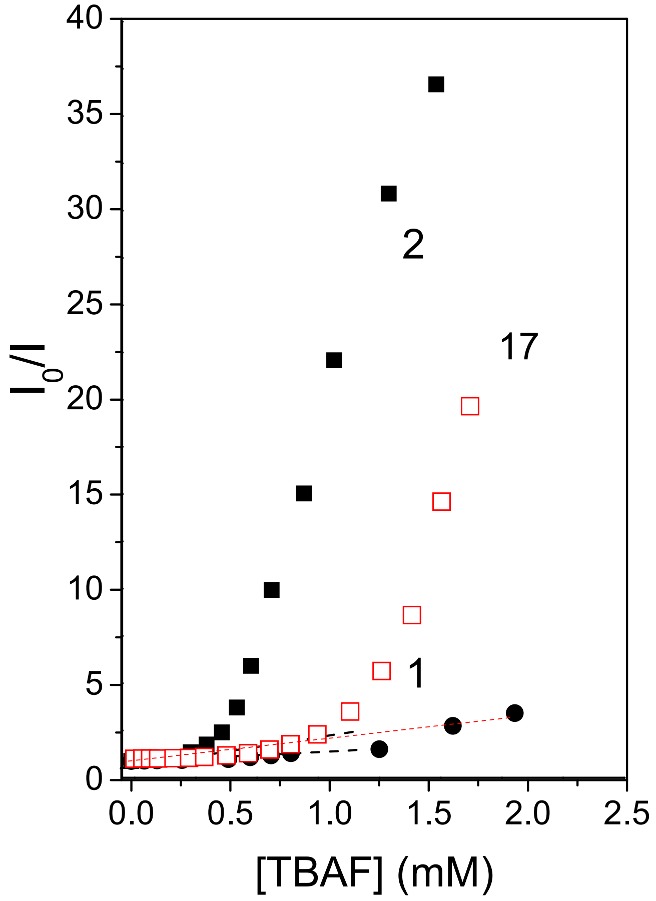

Figure S4. Stern-Vomer plots of fluorescence intensity measured as the area under emission spectra for **1**, **2** and **17** quenching upon TBAF addition at 25ºC ([**1**]=6.72x10^{-5} M, [**2**]=4.56x10^{-5} M and [**17**]=8.22x10^{-5} M; λ_{ex} =354 nm, 365 and 373 nm respectively). From the slope of the first portion of the curve binding constants (K_S) of **180±20**, **2100±400** and **2170±380 M^{-1}** for **1**, **2** and **17** fluoroborate complexes were obtained. Lifetimes ratios (τ_0/τ) were near 1 for any of the TBAF concentration measured. The fact that K_S values are rather similar for **2** and **17** confirms that the quenching of fluorescence in **2** (and probably for **1**) is due to the formation of fluoroborate complexes, discarding that quenching was due to the F^- binding to the NH via hydrogen bonding.

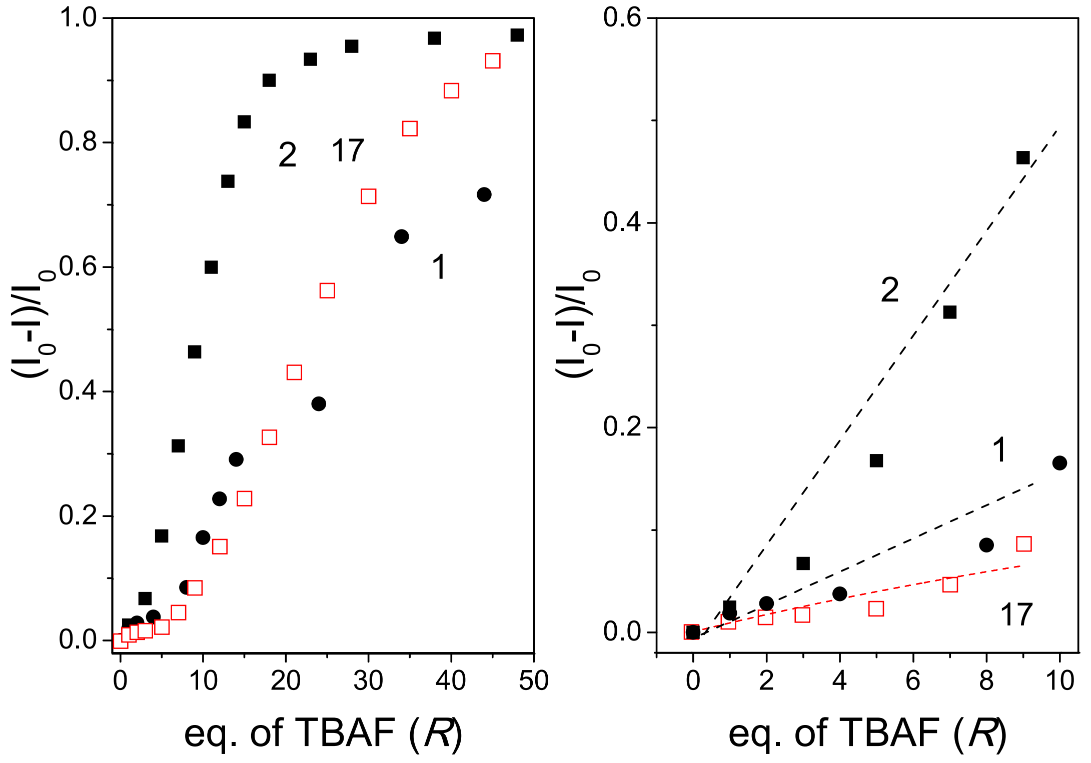

Figure S5. Normalized variation of the fluorescence intensities for **1**, **2** and **17** THF solutions at 25ºC (measured as the area under emission spectra) versus equivalents of TBAF added during titration ([**1**]=6.72x10^{-5} M, [**2**]=4.56x10^{-5} M and [**17**]=8.22x10^{-5} M; λ_{ex} =354 nm, 365 and 373 nm respectively). The first portion of the curves were adjusted to equation S12 to get binding constants (K) for the formation of the B-F adduct of **50±180**, **530±340** and **350±300** M^{-1} respectively. The fact K values are rather similar for **2** and **17** confirms that the quenching of fluorescence in **2** (and probably for **1**) is due to the formation of fluoroborate complexes, discarding that quenching was due to the F^- binding to the NH via hydrogen bonding.

Experimental evidence which confirms the presence of π-π *stacking* ground state aggregates of 13 BN-phenanthrene and 16 BN-tetraphene derivatives in solution.

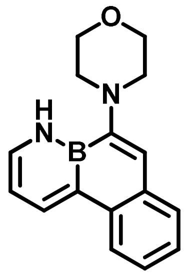

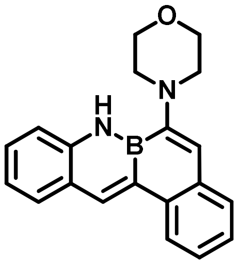

13 **16**

The **13 (16)** derivative fluorescence spectrum exhibits bands at 388 nm (364nm) and 521 nm (545 nm) in dilute solutions of cyclohexane. The second band located at 521 nm (545 nm) was attributed to the presence of rather stable ground state π-π stacking aggregates in cyclohexane solutions. This aggregation does not seem to occur for the other studied **1**, **11** and **12** BN-phenanthrene and **2**, **14** and **15** BN-tetraphene derivatives. Several experiments confirm this hypothesis:

i. The ratio of intensities of the emission spectra measured at 521 nm (545 nm), maximum of the band attributed to n-mers π-π stacking aggregates, and 388 nm (364 nm), due to the emission of the monomeric species, increases with the solution concentration of **13** (**16**) in cyclohexane. This agrees with the proposed aggregation in solution. The bands at 521 nm and 545 nm are responsible for the emission of such aggregate species (Figure S5).

ii. Emission spectra of **13** BN-phenanthrene and **16** BN-tetraphene derivatives in dilute solutions of cyclohexane, DMSO, acetonitrile, dichloromethane and formamide at 25ºC show that the intensity and location of the red-shifted band responsible for the emission of π-π stacking aggregates is sensitive to the nature of the solvent (Figure S6). In dichloromethane, the presence of aggregates considerably decreases.

iii. Emission spectra of **13** BN-phenanthrene and **16** BN-tetraphene derivatives in dilute solutions of cyclohexane by using several excitation wavelengths, depicted in Figure S7, show that the presence of the second red-shifted bands, located at 521 and 545 nm respectively, do not require the excitation of the monomer. This reinforces the idea that later bands can be attributed to species that are stable in the ground state, and not to the presence of intermolecular excimers.

iv. More complex fluorescence intensity decays at 388 and 521 nm (364 and 545 nm), upon 335nm excitation, than the single mono-exponential one, would be expected from the hypothetical presence of a monomer–excimer equilibrium in the excited state. This also discards the presence of intermolecular excimer. It again reinforces the idea of a ground state complex which emits at the wavelengths of the red shifted bands.

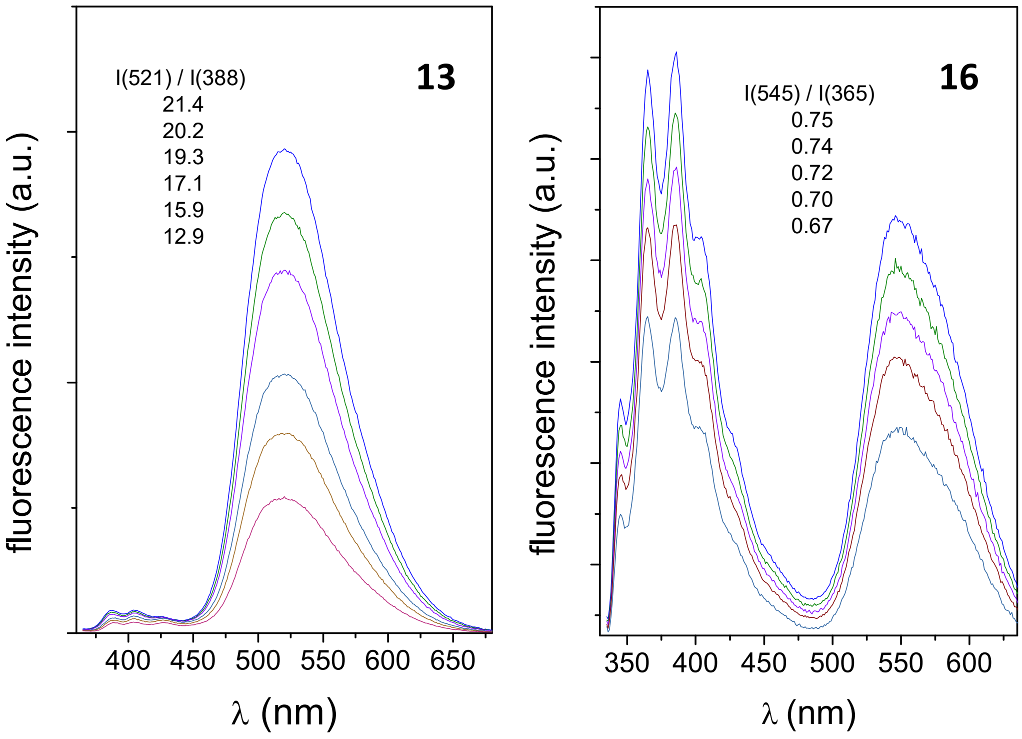

Figure S6. Emission spectra for solutions of **13** BN-phenanthrene and **16** BN-tetraphene derivatives in cyclohexane upon decreasing the concentration. Notice that the I(521)/I(388) and I(545)/I(365) ratios of intensities, decrease upon dilution. This means that, agreeing with aggregation, the amount of species stable in the ground state emitting at 521 and 545 nm increases with concentration. Dilutions start with cyclohexane solutions of **13** and **16** of approximately 0.1 absorbance at λ_{ex} of 350 nm and 323 nm respectively.

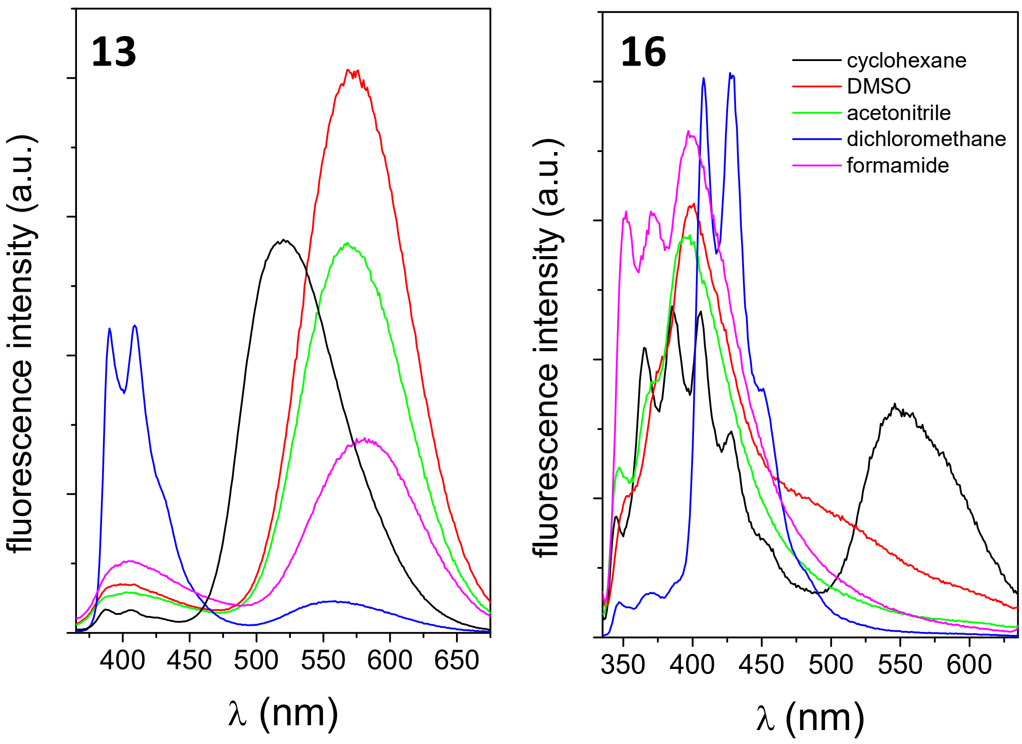

Figure S7. Emission spectra for solutions of **13** BN-phenanthrene and **16** BN-tetraphene derivatives in dilute solutions of cyclohexane, DMSO, acetonitrile, dichloromethane and formamide at 25ºC upon excitation of λ_{ex} =350 nm and 323 nm respectively. Intensity and location of the band responsible for the emission of π-π stacking aggregates is strongly dependent on the nature of the solvent and the type of derivative.

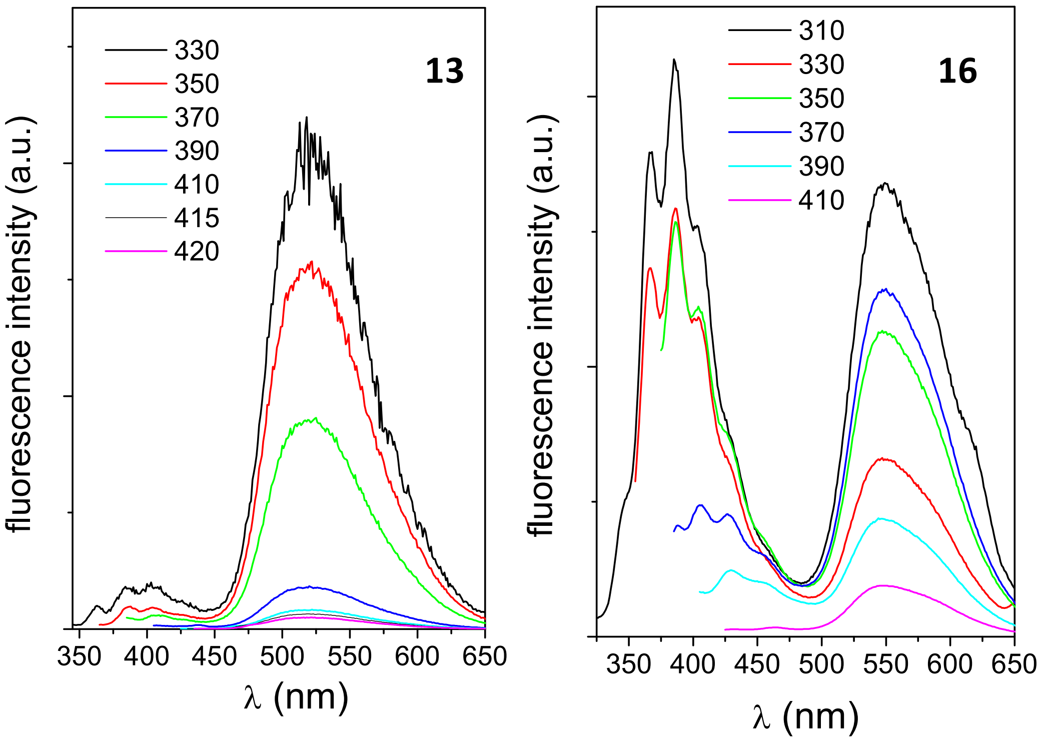

Figure S8. Emission spectra for solutions of **13** BN-phenanthrene and **16** BN-tetraphene derivatives in cyclohexane upon excitation at different wavelengths (shown in the graph) at 25ºC. Notice that the bands at 521 and 545 change in intensity with the excitation wavelength but even at the wavelengths were the monomer bands disappear (monomers were not excited), the bands still appear for both derivatives due to aggregation. The presence of the bands to the red do not require the excitation of the monomer.

X-RAY CRYSTALLOGRAPHIC DATA FOR 2

To crystallize compound **2**, it was dissolved in the minimum amount of toluene in an NMR tube, hexane was added slowly and allowed to stand at room temperature until crystals were formed.

Crystallographic data are presented in Tables S2-S8. A single crystal of **2** was coated in high-vacuum grease and mounted on a glass fibre. X-ray measurements were made using a Bruker D8 VENTURE PhtotonIII area-detector diffractometer with Cu-K_{α} radiation (α = 1.54 Å). Absorption corrections were applied, based on multiple and symmetry-equivalent measurements. The structure was solved by ShelXT structure solution program using Intrinsic Phasing and refined with the XL refinement package using Least Squares minimisation.[4]

All non-hydrogen atoms were assigned anisotropic displacement parameters and refined without positional constraints. H1 and H2A were assigned anisotropic displacement parameters and all other hydrogen atoms were constrained to ideal geometries and refined with fixed isotropic displacement parameters.

Refinement proceeded smoothly to give the residuals shown in Table S2.

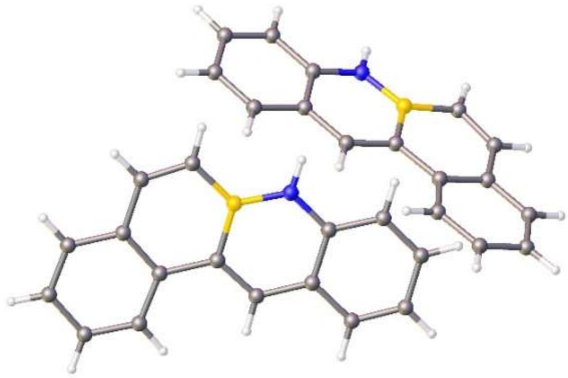

Figure S9. X-ray structure for **2**.

A colorless needle-like specimen of $C_{16}H_{12}BN$, approximate dimensions 0.010 mm x 0.030 mm x 0.200 mm, was used for the X-ray crystallographic analysis. The X-ray intensity data were measured (λ = 1.54184 Å).

[4] (a) Sheldrick, G.M. (2015). Acta Cryst A71, 3-8; APEX3 Version 2016.7 (Bruker AXS Inc.) Bruker Instrument Service vV6.2.10. (b) SAINT integration software, SAINT V8.38A (Bruker AXS Inc., 2017). (c) SADABS-2016/2 - Bruker AXS area detector scaling and absorption correction (Sheldrick, Bruker AXS Inc.). (d) *SHELXTL program system version 6.1;* XPREP Version 2013/3 (Sheldrick, Bruker AXS Inc.) XS Version 2013/1 (George M. Sheldrick, *Acta Cryst.* (2008). A**64**, 112-122). (e) *International Tables for Crystallography*, Kluwer, Dordrecht, 1992, vol. C.

Table S1: Data collection details for 2.

Axis	dx/mm	2θ/°	ω/°	ϕ/°	χ/°	Width/°	Frames	Time/s	Wavelength/Å	Voltage/kV	Current/mA	Temperature/K
Phi	39.907	108.58	12.50	0.00	24.00	1.80	200	10.00	1.54184	50	1.1	180
Phi	39.907	78.58	76.65	0.00	-44.50	1.80	200	10.00	1.54184	50	1.1	180
Omega	39.907	108.58	-7.18	-160.00	61.50	1.80	68	10.00	1.54184	50	1.1	180
Omega	39.907	108.58	-7.18	40.00	61.50	1.80	68	10.00	1.54184	50	1.1	180
Omega	39.907	-48.14	-54.69	-54.00	-61.50	1.80	62	10.00	1.54184	50	1.1	180
Omega	39.907	-18.14	-124.02	180.00	44.50	1.80	60	10.00	1.54184	50	1.1	180
Omega	39.907	-48.14	-54.69	153.00	-61.50	1.80	62	10.00	1.54184	50	1.1	180
Omega	39.907	-33.14	-132.80	270.00	44.50	1.80	57	10.00	1.54184	50	1.1	180
Phi	39.907	108.58	108.79	0.00	-24.00	1.80	200	10.00	1.54184	50	1.1	180
Omega	39.907	-33.14	-132.80	90.00	44.50	1.80	57	10.00	1.54184	50	1.1	180
Omega	39.907	108.58	-7.18	-120.00	61.50	1.80	68	10.00	1.54184	50	1.1	180
Omega	39.907	78.58	76.65	270.00	-44.50	1.80	55	10.00	1.54184	50	1.1	180
Phi	39.907	93.58	91.65	0.00	-44.50	1.80	200	10.00	1.54184	50	1.1	180
Omega	39.907	-33.14	-132.80	0.00	44.50	1.80	57	10.00	1.54184	50	1.1	180
Omega	39.907	108.58	-7.18	0.00	61.50	1.80	68	10.00	1.54184	50	1.1	180
Omega	39.907	108.58	-7.18	80.00	61.50	1.80	68	10.00	1.54184	50	1.1	180
Omega	39.907	93.58	91.65	180.00	-44.50	1.80	46	10.00	1.54184	50	1.1	180
Omega	39.907	108.58	-7.18	160.00	61.50	1.80	68	10.00	1.54184	50	1.1	180
Omega	39.907	93.58	91.65	270.00	-44.50	1.80	46	10.00	1.54184	50	1.1	180
Omega	39.907	78.58	76.65	90.00	-44.50	1.80	55	10.00	1.54184	50	1.1	180
Omega	39.907	78.58	76.65	180.00	-44.50	1.80	55	10.00	1.54184	50	1.1	180
Omega	39.907	-18.14	-124.02	0.00	44.50	1.80	60	10.00	1.54184	50	1.1	180
Omega	39.907	-18.14	-124.02	90.00	44.50	1.80	60	10.00	1.54184	50	1.1	180

A total of 1940 frames were collected. The total exposure time was 5.39 hours. The frames were integrated with the Bruker SAINT software package using a narrow-frame algorithm. The integration of the data using an orthorhombic unit cell yielded a total of 27480 reflections to a

maximum θ angle of 62.51° (0.87 Å resolution). The final cell constants of a = 6.1633(4) Å, b = 17.0506(9) Å, c = 23.0590(12) Å, volume = 2423.2(2) Å^3, are based upon the refinement of the XYZ-centroids of 9946 reflections above 20 σ(I) with 6.447° < 2θ < 122.0°. Data were corrected for absorption effects using the Multi-Scan method (SADABS). The ratio of minimum to maximum apparent transmission was 0.737.

Table S2 Crystal data and structure refinement for **2**.

Identification code	CCDC 2073370
Empirical formula	$C_{16}H_{12}BN$
Formula weight	229.08
Temperature/K	180.0
Crystal system	orthorhombic
Space group	$P2_12_12_1$
a/Å	6.1649(4)
b/Å	17.0435(11)
c/Å	23.0574(14)
α/°	90
β/°	90
γ/°	90
Volume/Å^3	2422.7(3)
Z	8
ρ_{calc}g/cm^3	1.256
μ/mm^{-1}	0.548
F(000)	960.0
Crystal size/mm^3	0.2 × 0.03 × 0.01
Radiation	CuKα (λ = 1.54178)
2Θ range for data collection/°	6.448 to 130.362
Index ranges	-7 ≤ h ≤ 5, -18 ≤ k ≤ 19, -22 ≤ l ≤ 27
Reflections collected	25107
Independent reflections	4067 [R_{int} = 0.0906, R_{sigma} = 0.0548]
Data/restraints/parameters	4067/0/334
Goodness-of-fit on F^2	1.041
Final R indexes [I>=2σ (I)]	R_1 = 0.0557, wR_2 = 0.1474
Final R indexes [all data]	R_1 = 0.1252, wR_2 = 0.2048
Largest diff. peak/hole / e Å^{-3}	0.14/-0.15
Flack parameter	-1.2(5)

Table S3 Fractional Atomic Coordinates (×10^4) and Equivalent Isotropic Displacement Parameters (Å^2×10^3) for **2**. U_{eq} is defined as 1/3 of of the trace of the orthogonalised U_{IJ} tensor.

**Atom**	***x***	***y***	***z***	**U(eq)**
N1	7113(9)	5893(3)	4229(2)	87.7(15)
N2	3129(12)	4369(4)	3688(3)	101.6(17)
C10	10833(9)	6714(3)	4549(2)	79.3(15)
C9	9409(8)	7107(3)	4204(2)	72.7(14)
C8	9739(9)	7911(4)	3995(2)	76.8(15)
C11	10506(9)	5929(3)	4738(3)	78.1(15)
C25	6548(11)	3754(4)	3324(3)	85.8(17)
C32	3124(12)	4896(4)	3235(3)	90.1(18)
C16	8596(10)	5519(4)	4575(3)	83.3(16)
C3	8151(11)	8243(4)	3628(3)	88.0(18)
C27	4878(11)	4881(4)	2846(3)	88.0(17)
C7	11531(10)	8367(4)	4137(2)	86.3(17)
C26	6551(12)	4309(4)	2899(3)	89.6(18)
C12	12025(11)	5537(4)	5087(3)	90.5(18)
C24	8221(13)	3153(4)	3390(3)	89.0(18)
C5	10226(16)	9447(4)	3572(3)	107(2)
C23	9893(14)	3025(4)	2987(3)	103(2)
C4	8461(14)	9015(4)	3427(3)	100(2)
C6	11787(13)	9124(4)	3929(3)	101.9(19)
C17	4796(16)	3209(5)	4279(3)	110(2)
C18	6447(16)	2711(4)	4306(3)	110(2)
C2	6233(11)	7806(5)	3451(3)	100(2)
C1	5769(10)	7075(5)	3620(3)	97.5(19)
C31	1465(13)	5444(5)	3160(3)	107(2)
C28	4904(14)	5445(4)	2399(3)	101(2)
C15	8306(12)	4755(4)	4770(3)	101.3(19)
C22	11487(16)	2459(5)	3061(3)	118(2)
C19	8176(15)	2653(4)	3878(3)	100(2)
C20	9810(17)	2090(4)	3946(3)	112(2)
C29	3263(16)	5977(4)	2339(3)	115(2)
C30	1512(15)	5960(5)	2719(4)	116(2)
C13	11682(14)	4776(4)	5267(3)	109(2)
C14	9803(16)	4387(4)	5111(3)	115(2)
B2	4709(16)	3791(5)	3764(3)	92(2)
C21	11445(18)	1981(4)	3554(4)	125(3)
B1	7360(11)	6672(5)	4014(3)	81.4(19)

Table S4 Anisotropic Displacement Parameters ($Å^2 \times 10^3$) for **2**. The Anisotropic displacement factor exponent takes the form: $-2\pi^2[h^2a^{*2}U_{11}+2hka^*b^*U_{12}+\ldots]$.

Atom	U_{11}	U_{22}	U_{33}	U_{23}	U_{13}	U_{12}
N1	75(3)	89(4)	99(4)	-13(3)	-11(3)	-6(3)
N2	99(4)	126(5)	79(4)	-8(4)	7(4)	-25(4)
C10	70(3)	84(4)	84(3)	-9(3)	-5(3)	-6(3)
C9	61(3)	82(4)	74(3)	-11(3)	1(3)	8(3)
C8	73(3)	87(4)	70(3)	-9(3)	10(3)	9(3)
C11	76(3)	73(4)	85(4)	-13(3)	2(3)	-2(3)
C25	100(4)	84(4)	74(4)	-2(3)	-11(4)	-29(4)
C32	102(5)	95(5)	73(4)	-6(4)	-13(4)	-26(4)
C16	78(4)	83(4)	89(4)	-16(3)	-2(4)	-8(3)
C3	91(5)	102(5)	71(3)	-6(3)	3(4)	23(4)
C27	97(4)	90(5)	77(4)	2(3)	-8(4)	-19(4)
C7	86(4)	82(4)	91(4)	-3(3)	8(4)	5(4)
C26	105(5)	96(4)	68(3)	5(3)	2(3)	-27(4)
C12	87(4)	86(4)	98(4)	-12(3)	-14(4)	6(4)
C24	112(5)	86(4)	69(4)	-1(3)	-5(4)	-28(4)
C5	129(6)	93(5)	98(5)	13(4)	23(5)	16(5)
C23	128(6)	90(5)	93(4)	-3(4)	-4(5)	-15(5)
C4	111(5)	108(5)	81(4)	9(4)	7(4)	25(5)
C6	104(5)	102(5)	100(4)	-7(4)	21(5)	-4(4)
C17	121(6)	126(6)	84(4)	9(4)	5(5)	-40(5)
C18	147(7)	101(6)	82(4)	13(4)	-5(5)	-50(5)
C2	88(5)	127(6)	85(4)	-2(4)	-8(3)	21(5)
C1	76(4)	122(5)	95(4)	-17(4)	-10(3)	5(4)
C31	93(5)	124(6)	105(5)	-27(5)	-8(5)	-5(5)
C28	117(5)	97(5)	90(4)	10(4)	-7(4)	-13(5)
C15	110(5)	80(5)	114(5)	-15(4)	0(5)	-15(4)
C22	143(6)	109(5)	103(5)	-14(4)	-6(5)	1(6)
C19	132(6)	85(5)	83(4)	5(4)	-22(5)	-31(5)
C20	155(7)	89(5)	92(5)	5(4)	-26(6)	-28(6)
C29	141(7)	106(6)	99(5)	5(4)	-25(6)	-13(6)
C30	119(6)	115(6)	115(6)	-4(5)	-31(6)	1(5)
C13	117(5)	87(5)	123(5)	-5(4)	-10(5)	13(5)
C14	147(7)	77(4)	122(6)	2(4)	-5(6)	-4(5)
B2	112(6)	95(5)	68(4)	0(4)	-4(5)	-41(5)
C21	154(8)	100(5)	121(6)	-4(5)	-35(6)	-12(5)
B1	70(4)	95(5)	78(4)	-19(4)	-1(4)	8(4)

Table S5 Bond Lengths for **2**.

Atom	Atom	Length/Å	Atom	Atom	Length/Å
N1	C16	1.370(7)	C27	C28	1.410(8)
N1	B1	1.426(9)	C7	C6	1.386(8)
N2	C32	1.378(8)	C12	C13	1.377(9)
N2	B2	1.396(10)	C24	C23	1.406(9)
C10	C9	1.361(7)	C24	C19	1.411(8)
C10	C11	1.422(8)	C5	C4	1.356(11)
C9	C8	1.466(8)	C5	C6	1.381(10)
C9	B1	1.530(8)	C23	C22	1.388(10)
C8	C3	1.412(8)	C17	C18	1.326(10)
C8	C7	1.390(8)	C17	B2	1.549(10)
C11	C16	1.419(7)	C18	C19	1.455(10)
C11	C12	1.403(8)	C2	C1	1.336(9)
C25	C26	1.363(8)	C1	B1	1.504(9)
C25	C24	1.462(9)	C31	C30	1.344(10)
C25	B2	1.522(10)	C28	C29	1.365(10)
C32	C27	1.405(9)	C15	C14	1.365(9)
C32	C31	1.396(9)	C22	C21	1.399(10)
C16	C15	1.389(8)	C19	C20	1.400(10)
C3	C4	1.407(9)	C20	C21	1.367(11)
C3	C2	1.456(10)	C29	C30	1.390(10)
C27	C26	1.424(9)	C13	C14	1.383(10)

Table S6 Bond Angles for **2**.

Atom	Atom	Atom	Angle/°	Atom	Atom	Atom	Angle/°
C16	N1	B1	124.3(6)	C23	C24	C25	123.8(6)
C32	N2	B2	123.8(7)	C23	C24	C19	116.7(7)
C9	C10	C11	123.5(5)	C19	C24	C25	119.5(7)
C10	C9	C8	124.3(5)	C4	C5	C6	119.3(7)
C10	C9	B1	117.5(5)	C22	C23	C24	123.0(7)
C8	C9	B1	118.2(5)	C5	C4	C3	122.4(7)
C3	C8	C9	118.4(6)	C5	C6	C7	119.9(7)
C7	C8	C9	123.7(5)	C18	C17	B2	118.2(7)
C7	C8	C3	117.9(6)	C17	C18	C19	125.0(7)
C16	C11	C10	119.9(6)	C1	C2	C3	124.7(6)
C12	C11	C10	122.0(5)	C2	C1	B1	117.6(6)
C12	C11	C16	118.1(6)	C30	C31	C32	121.0(8)
C26	C25	C24	124.1(6)	C29	C28	C27	121.2(7)

Table S6 Bond Angles for **2**. (continued)

**Atom**	**Atom**	**Atom**	**Angle/°**	**Atom**	**Atom**	**Atom**	**Angle/°**
C26	C25	B2	116.9(7)	C14	C15	C16	122.0(7)
C24	C25	B2	119.0(6)	C23	C22	C21	119.5(8)
N2	C32	C27	118.0(7)	C24	C19	C18	120.9(8)
N2	C32	C31	122.1(7)	C20	C19	C24	119.2(8)
C31	C32	C27	119.8(6)	C20	C19	C18	119.8(7)
N1	C16	C11	118.7(6)	C21	C20	C19	123.4(8)
N1	C16	C15	122.5(6)	C28	C29	C30	119.9(7)
C15	C16	C11	118.8(6)	C31	C30	C29	120.4(8)
C8	C3	C2	121.7(6)	C12	C13	C14	120.1(7)
C4	C3	C8	118.6(7)	C15	C14	C13	119.7(7)
C4	C3	C2	119.7(7)	N2	B2	C25	117.7(6)
C32	C27	C26	121.1(6)	N2	B2	C17	124.9(7)
C32	C27	C28	117.5(7)	C25	B2	C17	117.3(8)
C28	C27	C26	121.4(7)	C20	C21	C22	118.1(8)
C6	C7	C8	122.0(6)	N1	B1	C9	116.1(6)
C25	C26	C27	122.4(6)	N1	B1	C1	124.5(6)
C13	C12	C11	121.2(7)	C1	B1	C9	119.4(6)

Table S7 Torsion Angles for **2**.

**A**	**B**	**C**	**D**	**Angle/°**	**A**	**B**	**C**	**D**	**Angle/°**
N1	C16	C15	C14	179.5(6)	C26	C25	C24	C23	-7.1(9)
N2	C32	C27	C26	3.6(8)	C26	C25	C24	C19	174.0(5)
N2	C32	C27	C28	-177.3(5)	C26	C25	B2	N2	2.1(8)
N2	C32	C31	C30	179.0(6)	C26	C25	B2	C17	-175.0(6)
C10	C9	C8	C3	178.6(5)	C26	C27	C28	C29	177.9(6)
C10	C9	C8	C7	-1.5(8)	C12	C11	C16	N1	-179.2(5)
C10	C9	B1	N1	1.4(7)	C12	C11	C16	C15	0.5(8)
C10	C9	B1	C1	-178.4(5)	C12	C13	C14	C15	1.0(11)
C10	C11	C16	N1	0.8(8)	C24	C25	C26	C27	179.4(5)
C10	C11	C16	C15	-179.5(6)	C24	C25	B2	N2	-179.6(5)
C10	C11	C12	C13	179.9(6)	C24	C25	B2	C17	3.3(8)
C9	C10	C11	C16	-1.0(8)	C24	C23	C22	C21	0.7(10)
C9	C10	C11	C12	179.0(5)	C24	C19	C20	C21	-1.7(10)
C9	C8	C3	C4	179.9(5)	C23	C24	C19	C18	-176.9(6)
C9	C8	C3	C2	-0.8(8)	C23	C24	C19	C20	2.5(8)
C9	C8	C7	C6	179.8(5)	C23	C22	C21	C20	0.2(10)
C8	C9	B1	N1	-180.0(5)	C4	C3	C2	C1	-179.4(6)
C8	C9	B1	C1	0.3(7)	C4	C5	C6	C7	-0.4(10)

Table S7 Torsion Angles for **2**. (continued)

A	B	C	D	Angle/°	A	B	C	D	Angle/°
C8	C3	C4	C5	0.0(9)	C6	C5	C4	C3	0.2(10)
C8	C3	C2	C1	1.4(9)	C17	C18	C19	C24	0.9(10)
C8	C7	C6	C5	0.4(9)	C17	C18	C19	C20	-178.4(7)
C11	C10	C9	C8	-178.7(5)	C18	C17	B2	N2	-177.3(6)
C11	C10	C9	B1	-0.1(8)	C18	C17	B2	C25	-0.4(9)
C11	C16	C15	C14	-0.2(9)	C18	C19	C20	C21	177.7(6)
C11	C12	C13	C14	-0.8(10)	C2	C3	C4	C5	-179.3(6)
C25	C24	C23	C22	179.0(6)	C2	C1	B1	N1	-179.6(6)
C25	C24	C19	C18	2.1(8)	C2	C1	B1	C9	0.2(9)
C25	C24	C19	C20	-178.5(5)	C31	C32	C27	C26	-177.5(5)
C32	N2	B2	C25	1.1(9)	C31	C32	C27	C28	1.6(8)
C32	N2	B2	C17	178.0(6)	C28	C27	C26	C25	-179.5(5)
C32	C27	C26	C25	-0.3(9)	C28	C29	C30	C31	2.7(11)
C32	C27	C28	C29	-1.3(9)	C19	C24	C23	C22	-2.1(9)
C32	C31	C30	C29	-2.3(10)	C19	C20	C21	C22	0.3(11)
C16	N1	B1	C9	-1.7(8)	B2	N2	C32	C27	-3.9(9)
C16	N1	B1	C1	178.1(6)	B2	N2	C32	C31	177.2(6)
C16	C11	C12	C13	0.0(9)	B2	C25	C26	C27	-2.5(8)
C16	C15	C14	C13	-0.5(11)	B2	C25	C24	C23	174.8(5)
C3	C8	C7	C6	-0.2(8)	B2	C25	C24	C19	-4.1(8)
C3	C2	C1	B1	-1.0(9)	B2	C17	C18	C19	-1.7(10)
C27	C32	C31	C30	0.1(9)	B1	N1	C16	C11	0.5(8)
C27	C28	C29	C30	-0.8(10)	B1	N1	C16	C15	-179.1(6)
C7	C8	C3	C4	0.0(8)	B1	C9	C8	C3	0.0(7)
C7	C8	C3	C2	179.3(5)	B1	C9	C8	C7	180.0(5)

Table S8 Hydrogen Atom Coordinates (Å×10^4) and Isotropic Displacement Parameters (Å^2×10^3) for **2**.

Atom	*x*	*y*	*z*	U(eq)
H10	12113.33	6977.38	4669.52	95
H7	12612.68	8153.05	4384.44	104
H26	7709.31	4313.98	2626.96	108
H12	13313.57	5800.68	5200.73	109
H5	10386.43	9966.98	3429.59	128
H23	9933.63	3341.64	2647.41	124
H4	7395.88	9240.64	3180.15	120
H6	13034.46	9420.49	4031.72	122
H17	3696.75	3207.57	4567.89	132
H18	6506.71	2366.52	4629.09	132

Table S8 Hydrogen Atom Coordinates (Å×10^4) and Isotropic Displacement Parameters (Å^2×10^3) for **2**. (continued)

**Atom**	***x***	***y***	***z***	**U(eq)**
H2	5240.91	8058.99	3197.09	120
H1A	4480.09	6820.1	3495.45	117
H31	283.58	5452.46	3424.26	129
H28	6085.74	5455.66	2134.7	122
H15	7027.27	4480.62	4662.37	122
H22	12597.59	2397.07	2778.81	142
H20	9781.86	1767.75	4281.98	134
H29	3317.16	6357.35	2038.7	139
H30	342.02	6315.95	2667.01	139
H13	12738.34	4518.95	5499.03	131
H14	9554.6	3865.28	5239.93	138
H21	12522.57	1590.72	3613.92	150
H1	5940(100)	5600(30)	4160(30)	100(20)
H2A	2120(120)	4360(40)	3950(30)	110(20)

Verification of fluoride titrations of BN-phenanthrene 1 by ^{19}F, ^{11}B and ^{10}B NMR measurements

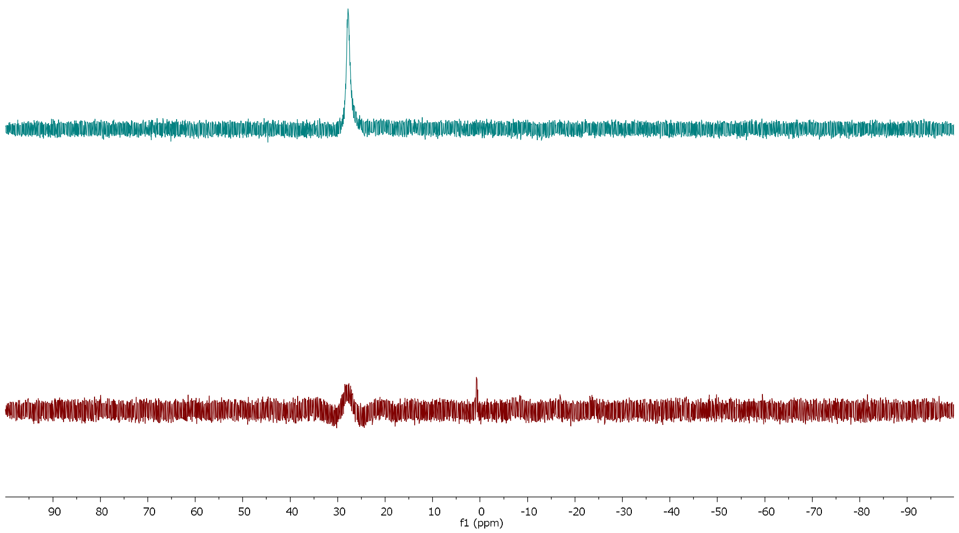

Figure S10. ^{11}B NMR of BN-phenanthrene **1** in THF-d_8 before (up) and after (down) the addition of 4 equivalents of TBAF.

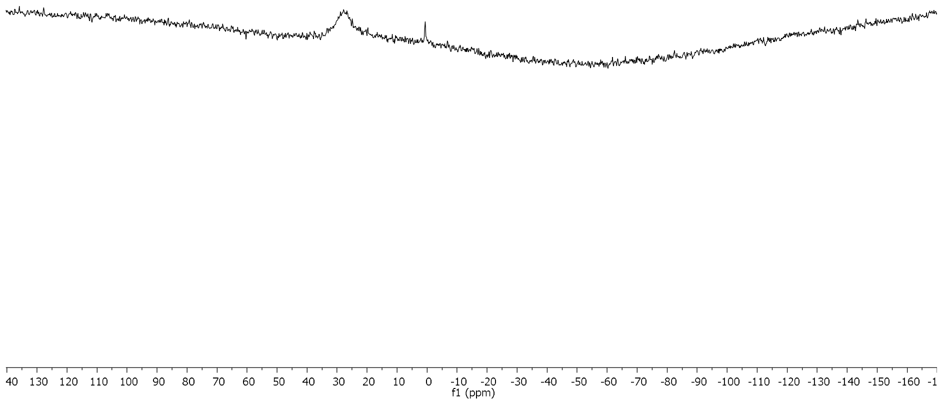

Figure S11. ^{10}B NMR of BN-phenanthrene **1** in THF-d_8 after the addition of 4 equivalents of TBAF.

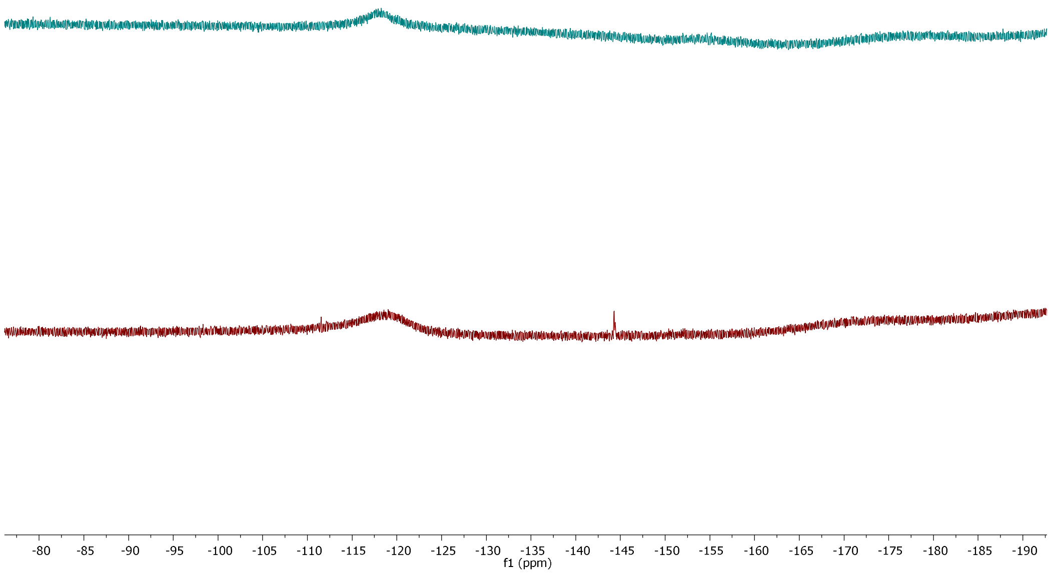

Figure S12. ^{19}F NMR of TBAF (up) and BN-phenanthrene **1** after the addition of 4 equivalents of TBAF (down) in THF-d_8.

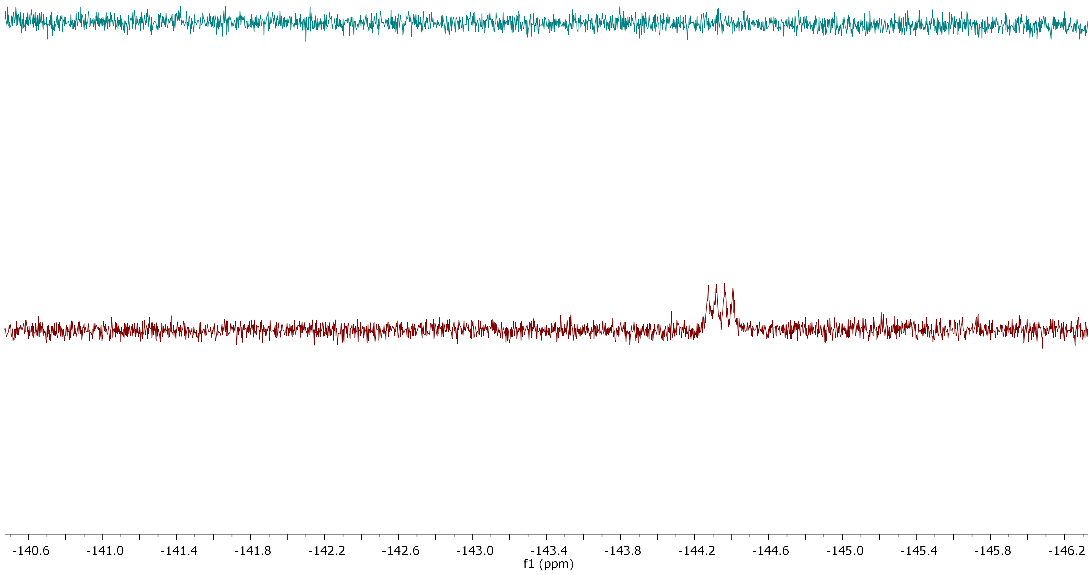

Figure S13. ^{19}F NMR of TBAF (up) and BN-phenanthrene **1** after the addition of 4 equivalents of TBAF (down) in THF-d_8 (enlargement).

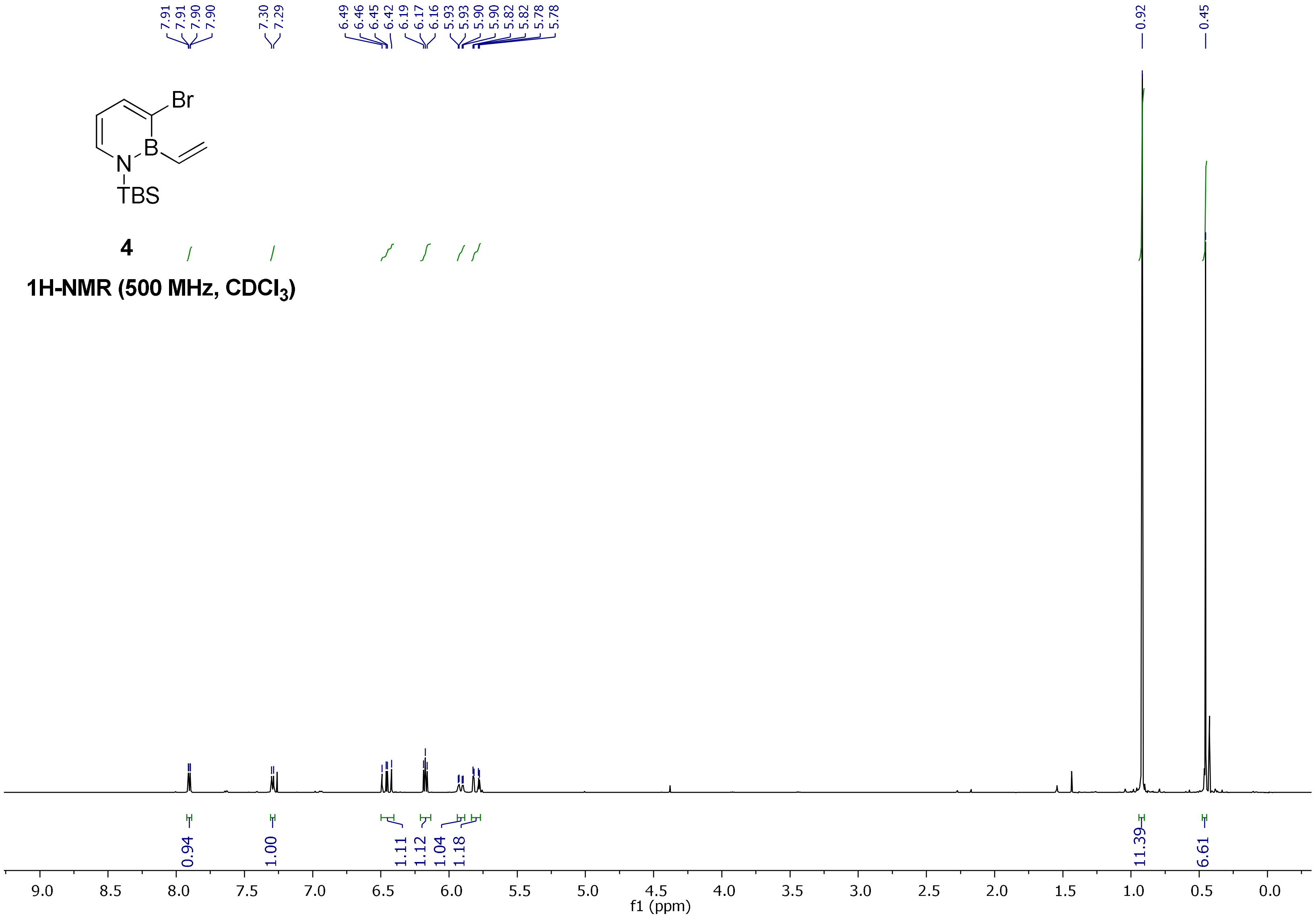

7.91
7.91
7.90
7.90
7.30
7.29
6.49
6.46
6.45
6.42
6.19
6.17
6.16
5.93
5.93
5.90
5.90
5.82
5.82
5.78
5.78
0.92
0.45
Br
B
N
TBS
4
1H-NMR (500 MHz, CDCl3)
0.94
1.00
1.11
1.12
1.04
1.18
11.39
6.61
9.0
8.5
8.0
7.5
7.0
6.5
6.0
5.5
5.0
4.5
4.0
3.5
3.0
2.5
2.0
1.5
1.0
0.5
0.0
f1 (ppm)

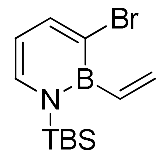

4

^{13}C-NMR (125 MHz, CDCl$_3$)

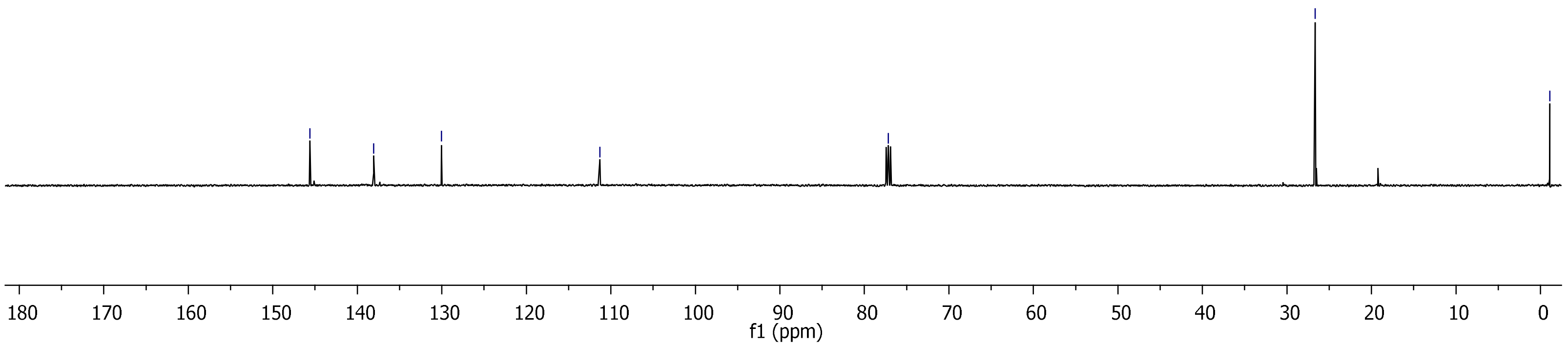

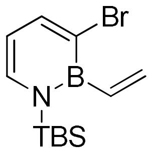

4

^{11}B-NMR (160 MHz, $CDCl_3$)

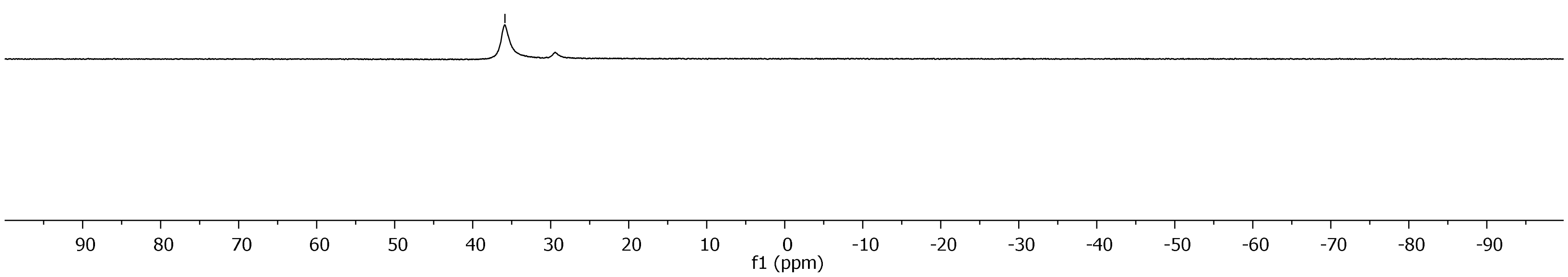

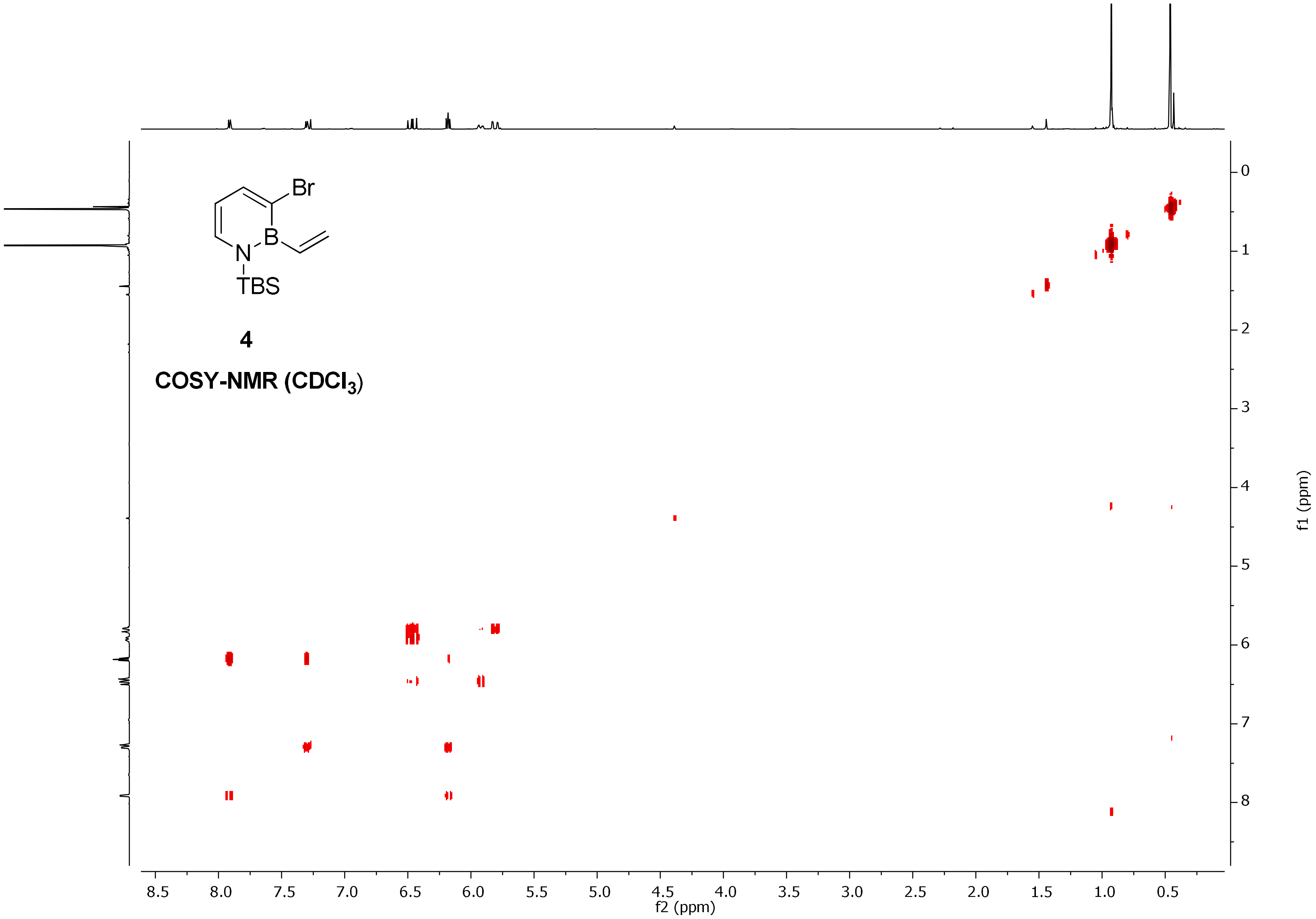

Br
B
N
TBS
4
COSY-NMR ($CDCl_3$)
0
1
2
3
4
5
6
7
8
f1 (ppm)
8.5
8.0
7.5
7.0
6.5
6.0
5.5
5.0
4.5
4.0
3.5
3.0
2.5
2.0
1.5
1.0
0.5
f2 (ppm)

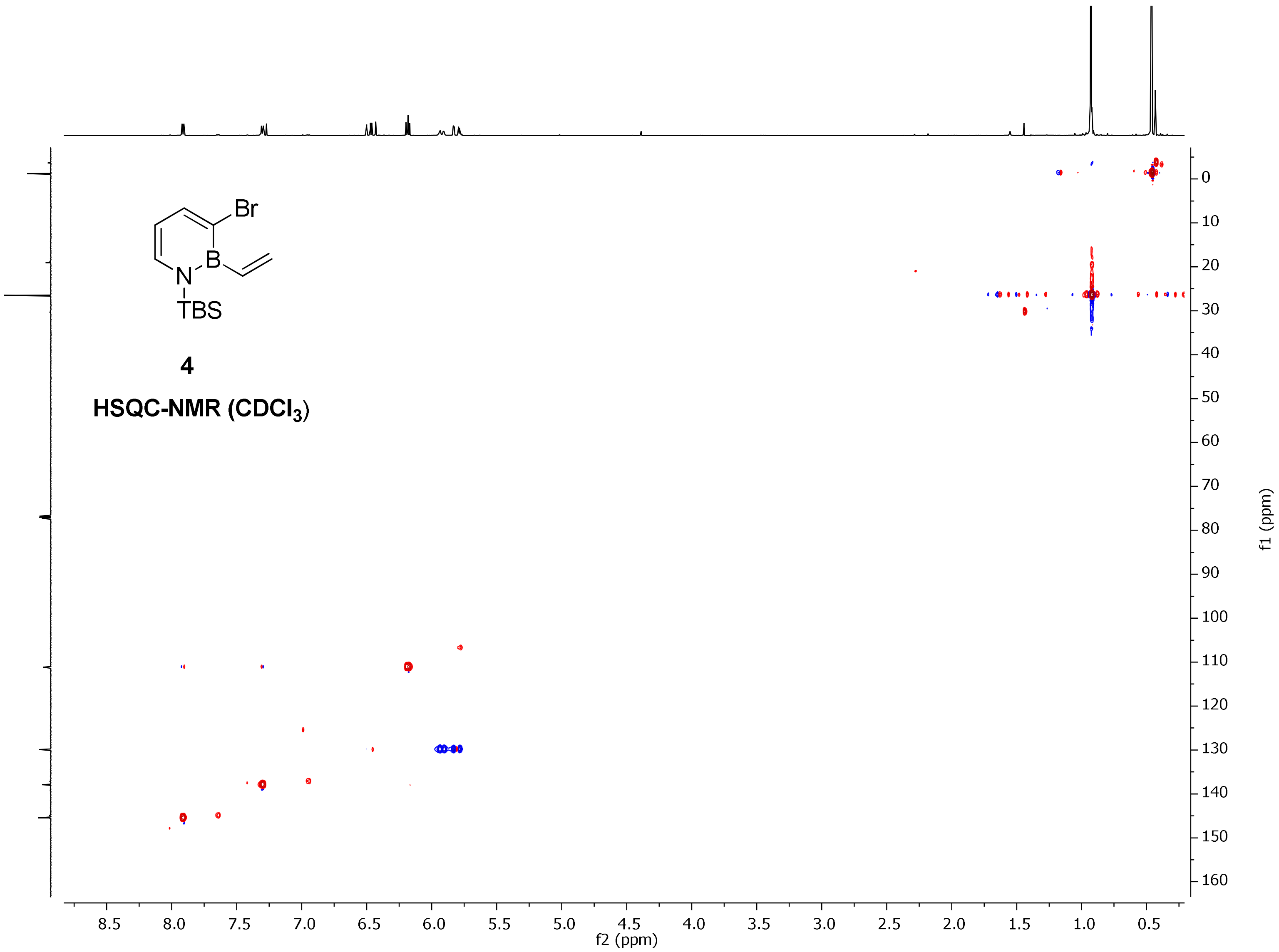
Br
B
N
TBS
4
HSQC-NMR (CDCl$_3$)
8.5 8.0 7.5 7.0 6.5 6.0 5.5 5.0 4.5 4.0 3.5 3.0 2.5 2.0 1.5 1.0 0.5
f2 (ppm)
0 10 20 30 40 50 60 70 80 90 100 110 120 130 140 150 160
f1 (ppm)

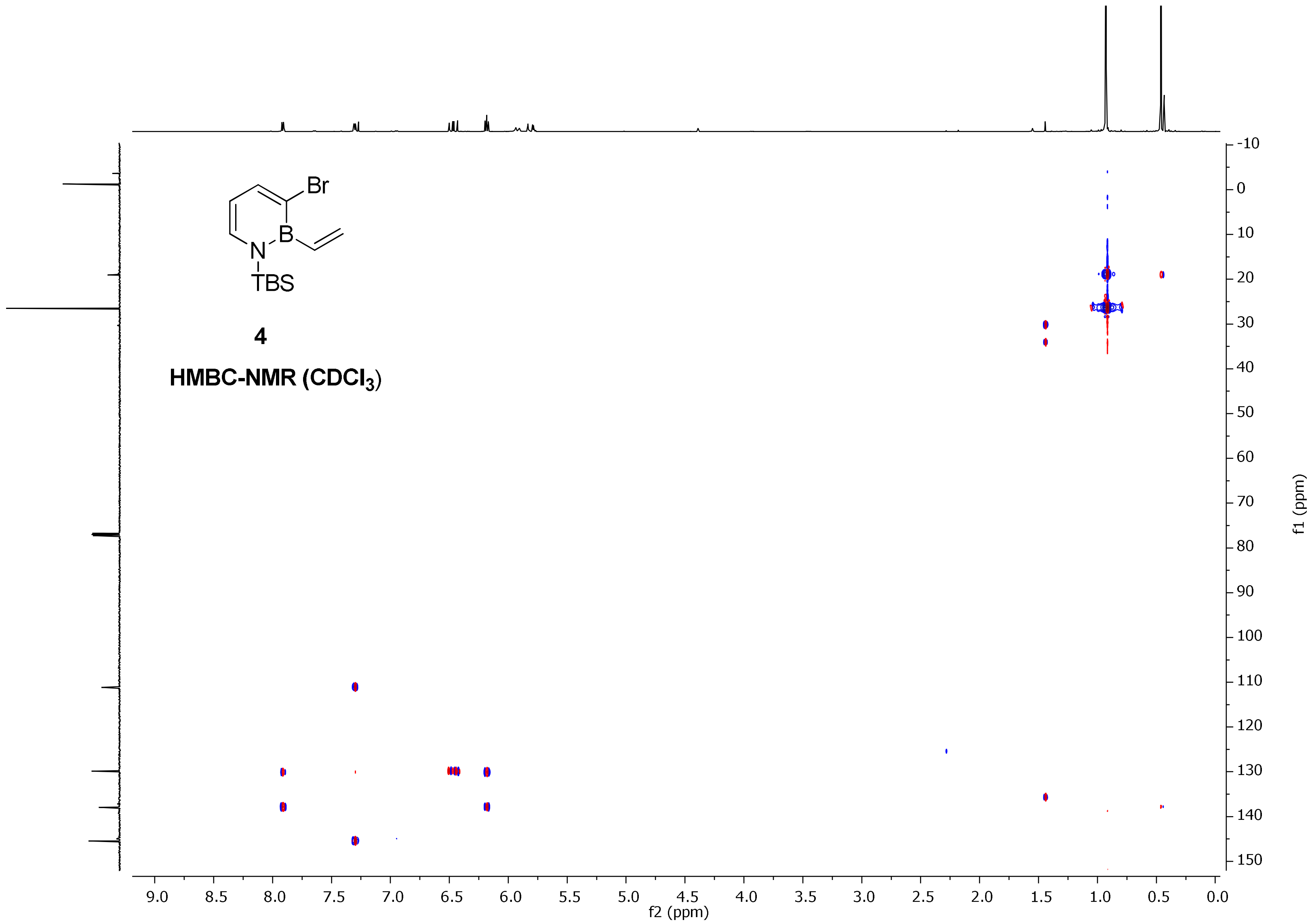
Br
B
N
TBS
4
HMBC-NMR (CDCl3)
f2 (ppm)
f1 (ppm)

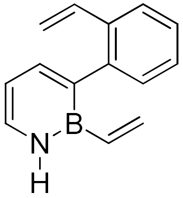

5

^{1}H-NMR (500 MHz, $CDCl_3$)

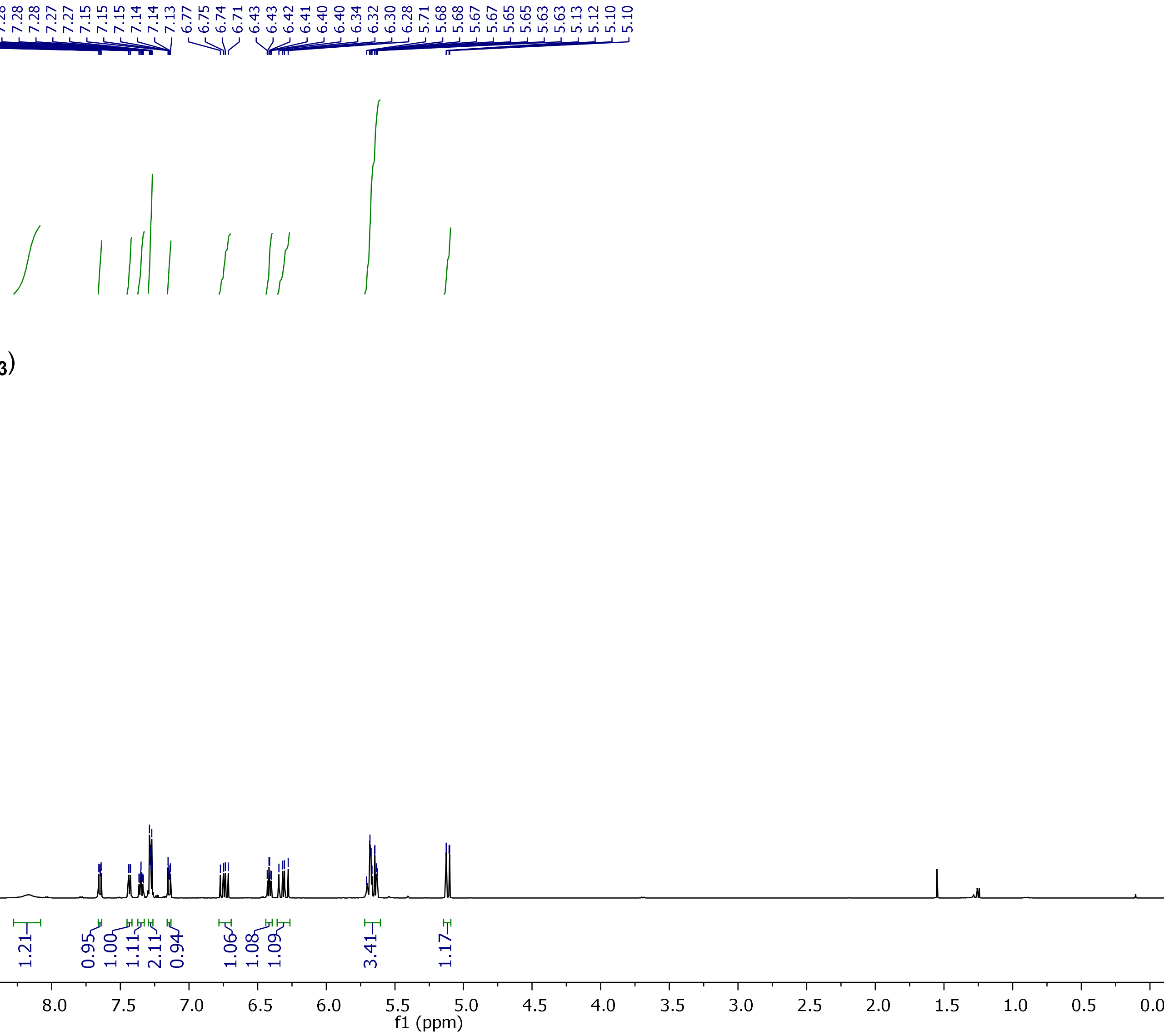

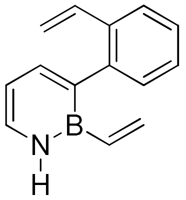

5

^{13}C-NMR (125 MHz, CDCl$_3$)

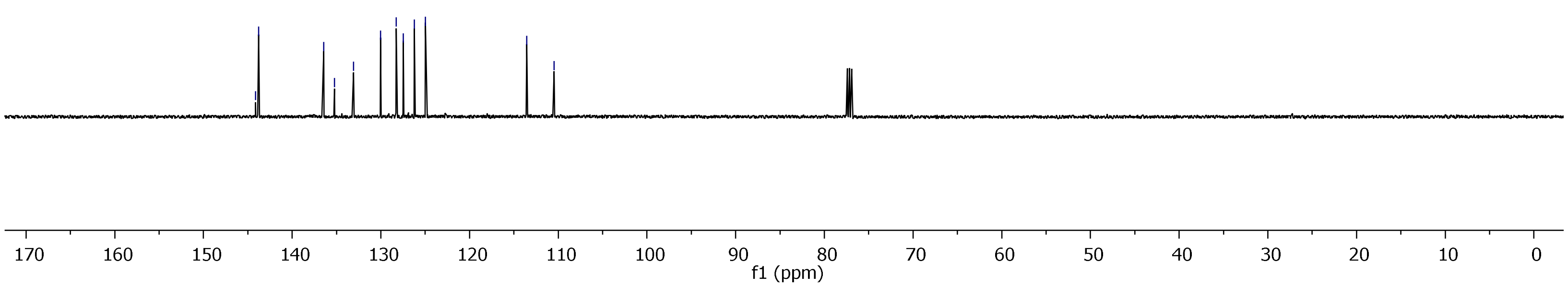

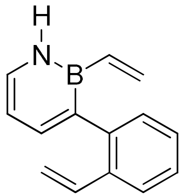

5

^{11}B-NMR (160 MHz, $CDCl_3$)

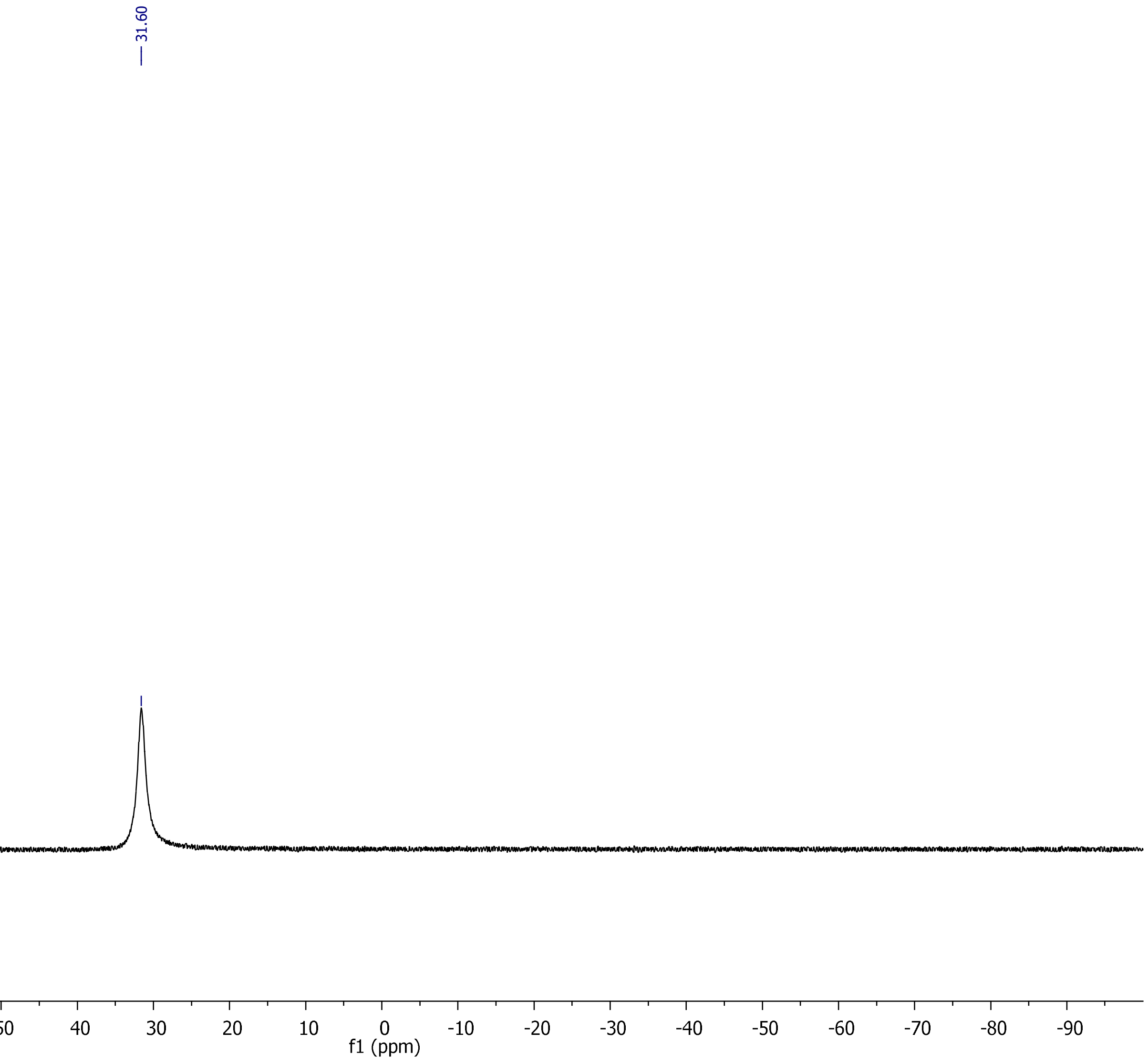

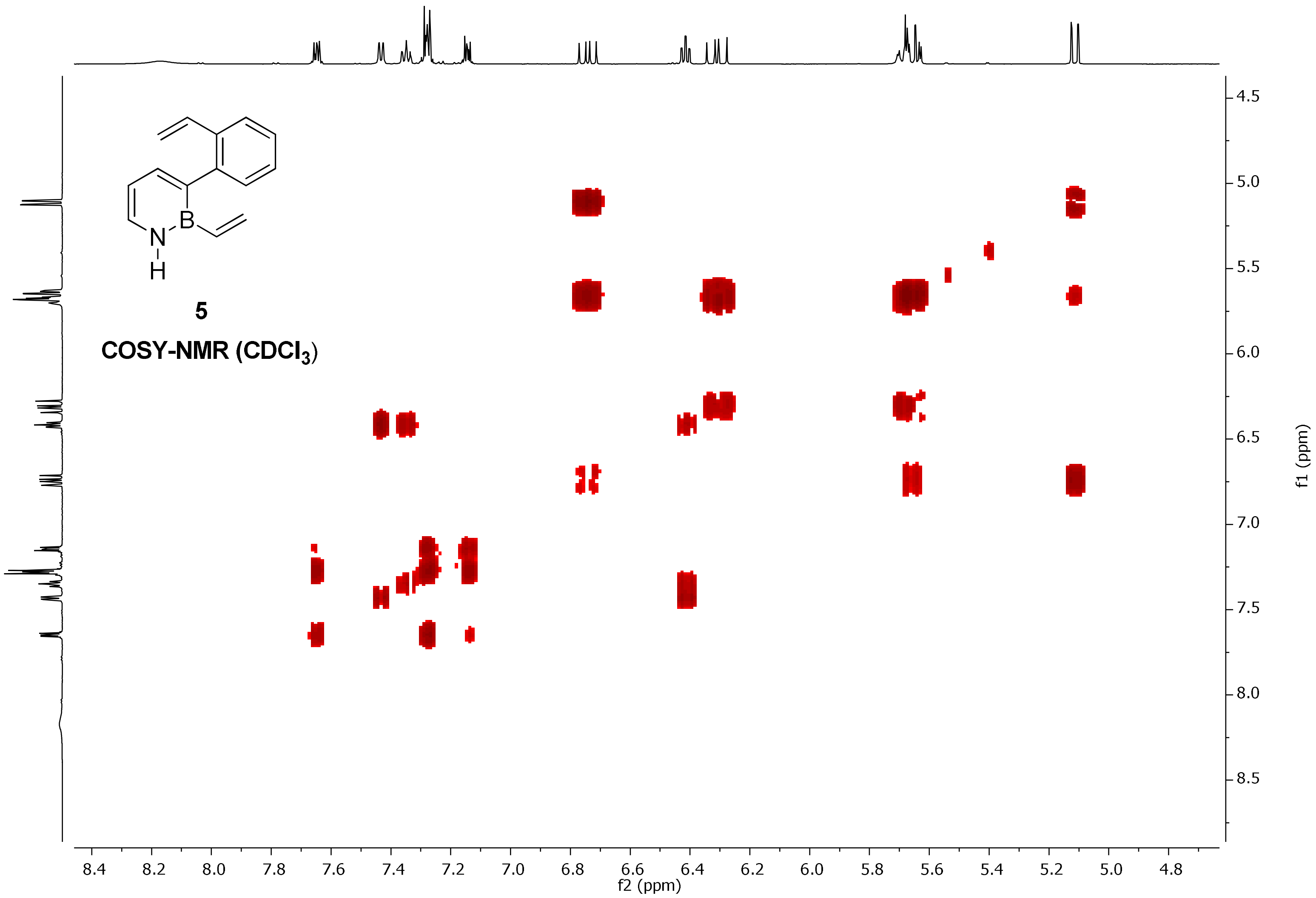
N
B
H
5
COSY-NMR (CDCl3)
4.5
5.0
5.5
6.0
6.5
7.0
7.5
8.0
8.5
f1 (ppm)
8.4
8.2
8.0
7.8
7.6
7.4
7.2
7.0
6.8
6.6
6.4
6.2
6.0
5.8
5.6
5.4
5.2
5.0
4.8
f2 (ppm)

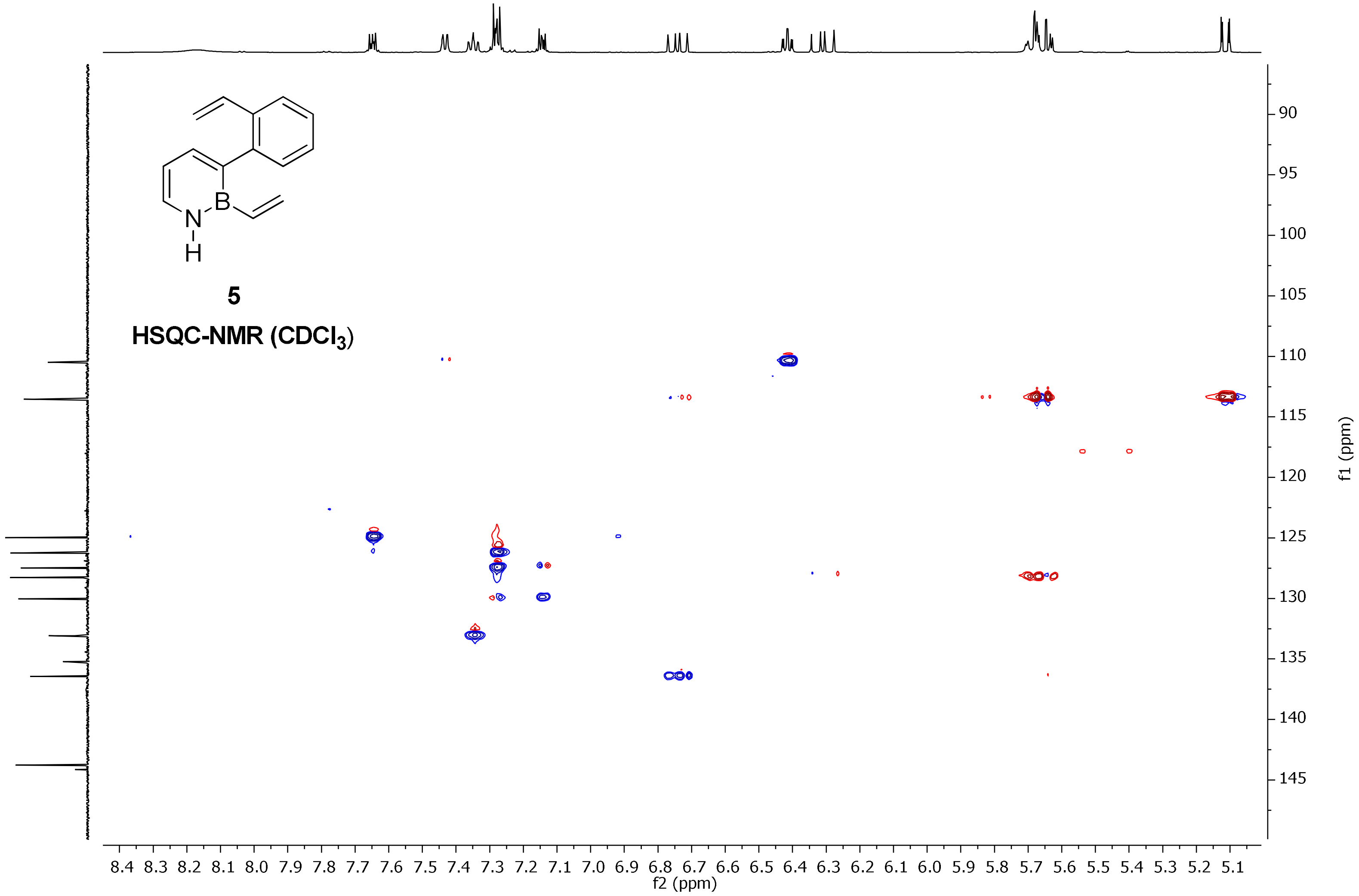

5
HSQC-NMR ($CDCl_3$)
f2 (ppm)
f1 (ppm)

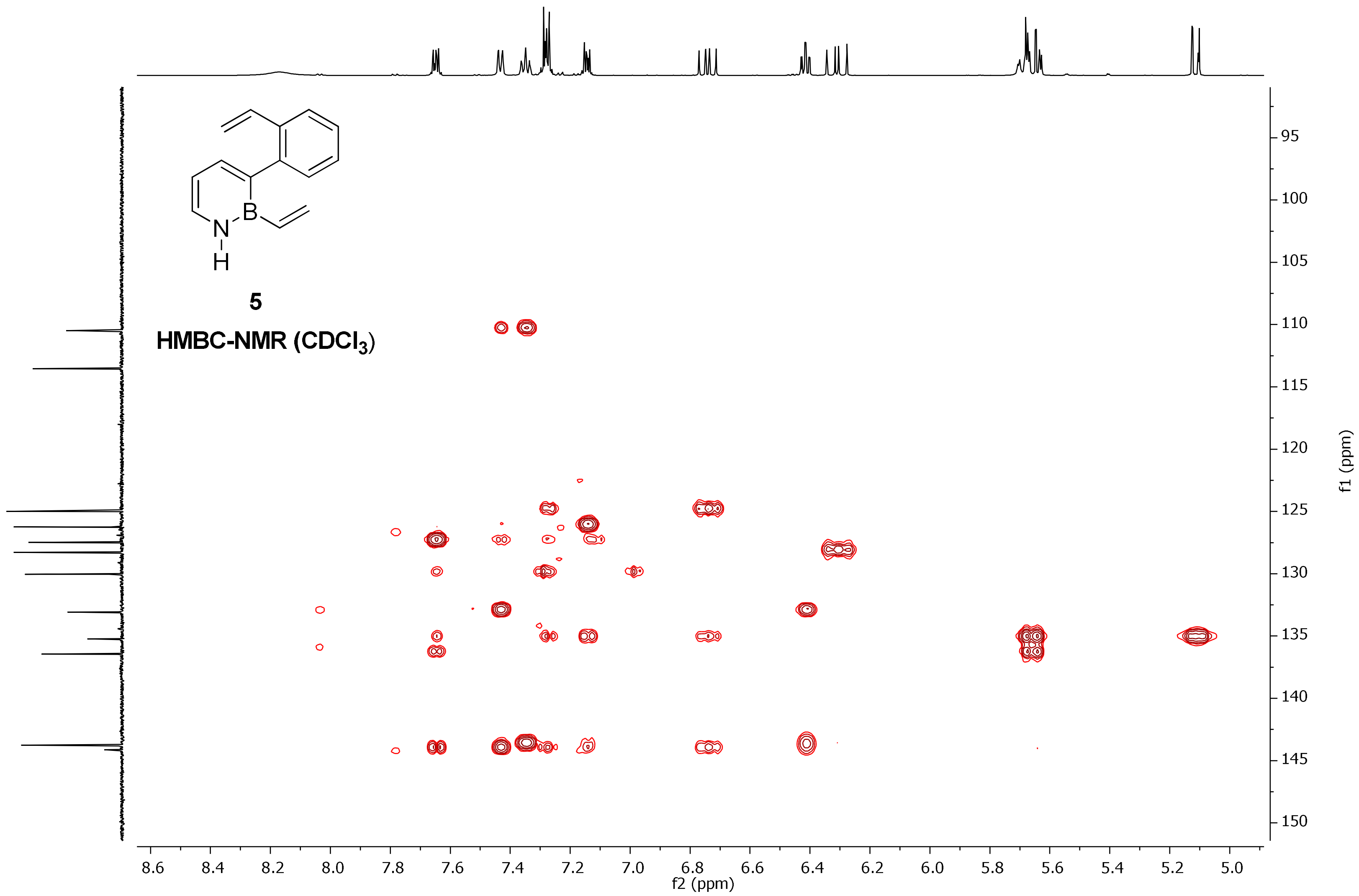

5
HMBC-NMR (CDCl3)
f2 (ppm)
f1 (ppm)

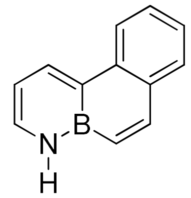

1

^{1}H-NMR (300 MHz, $CDCl_3$)

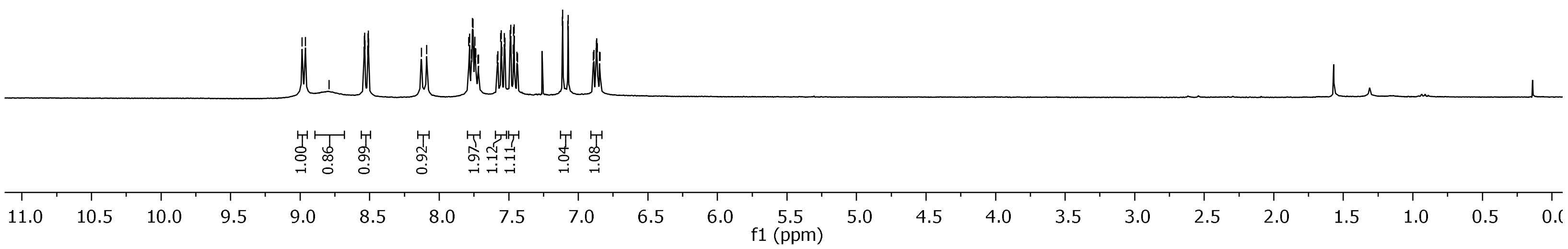

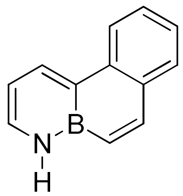

1

^{13}C-NMR (125 MHz, CDCl$_3$)

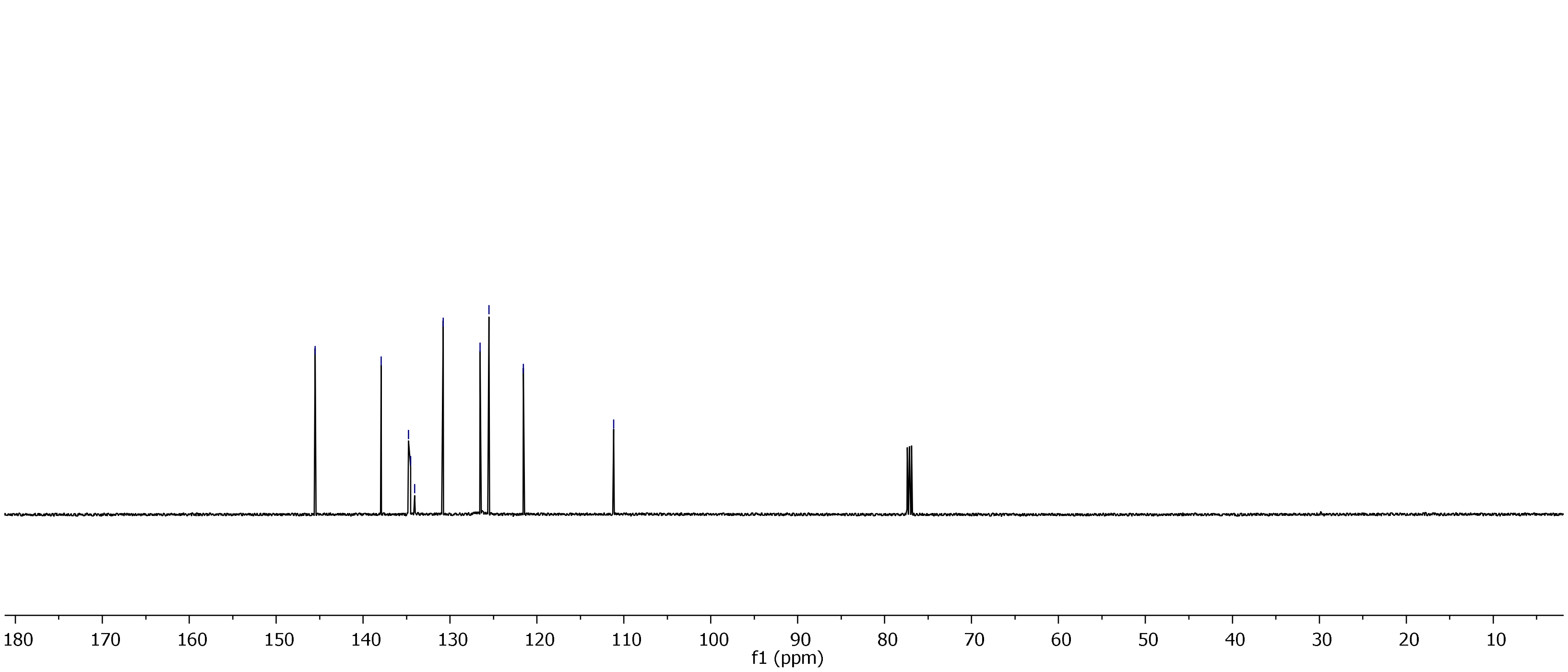

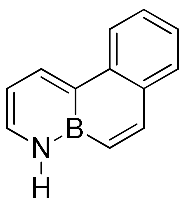

1

^{11}B-NMR (160 MHz, $CDCl_3$)

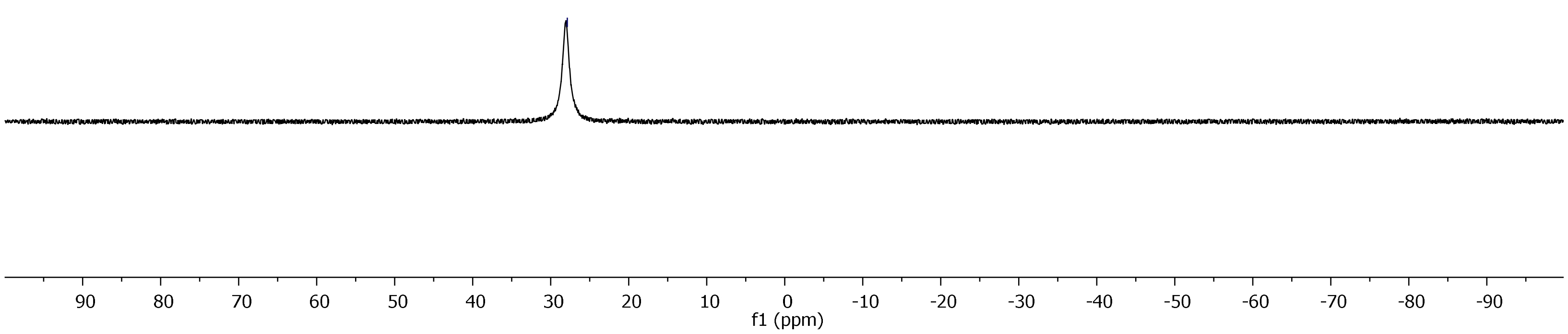

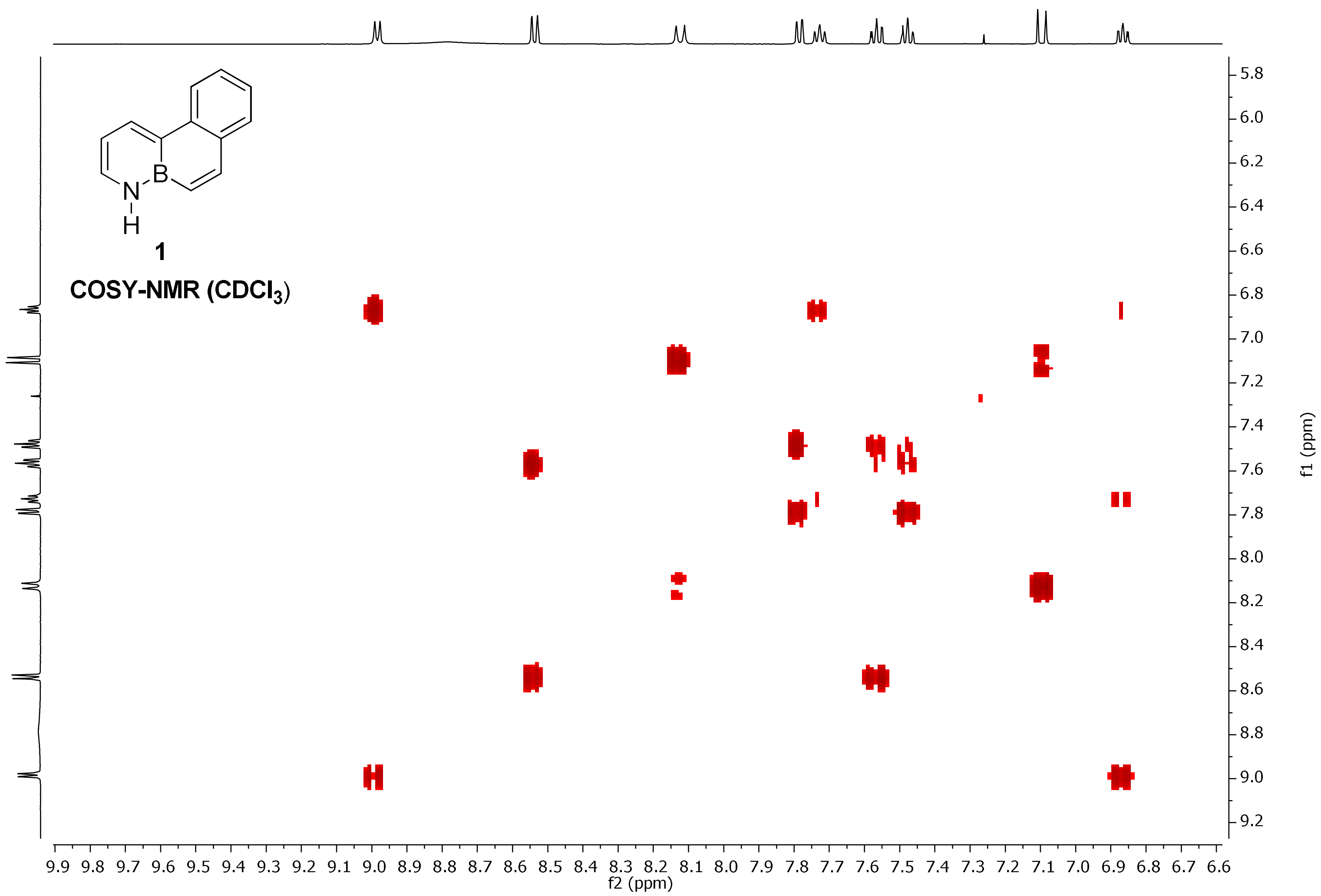

N
B
H
1
COSY-NMR ($CDCl_3$)
f2 (ppm)
f1 (ppm)

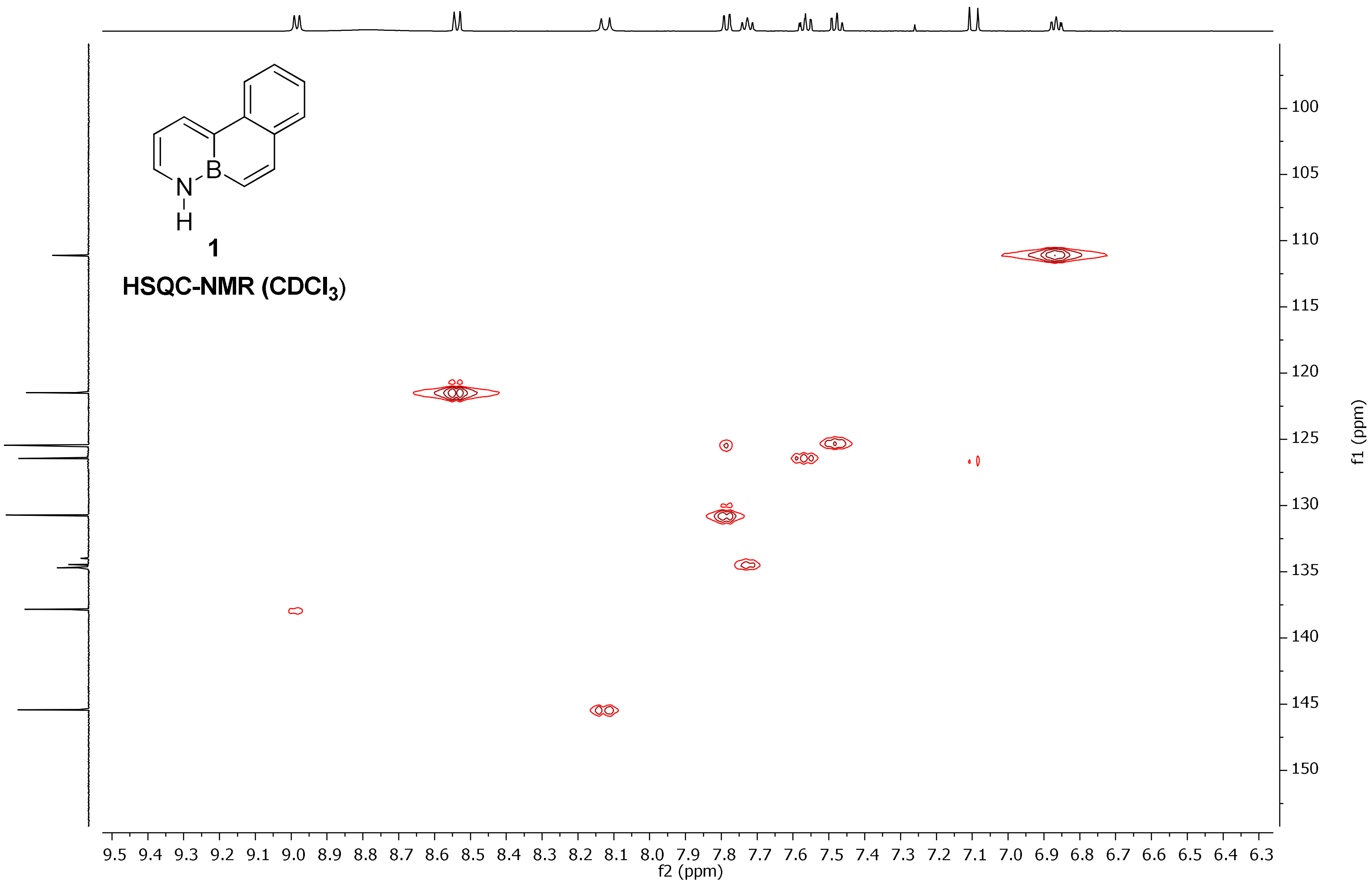
1
HSQC-NMR (CDCl3)
f2 (ppm)
f1 (ppm)

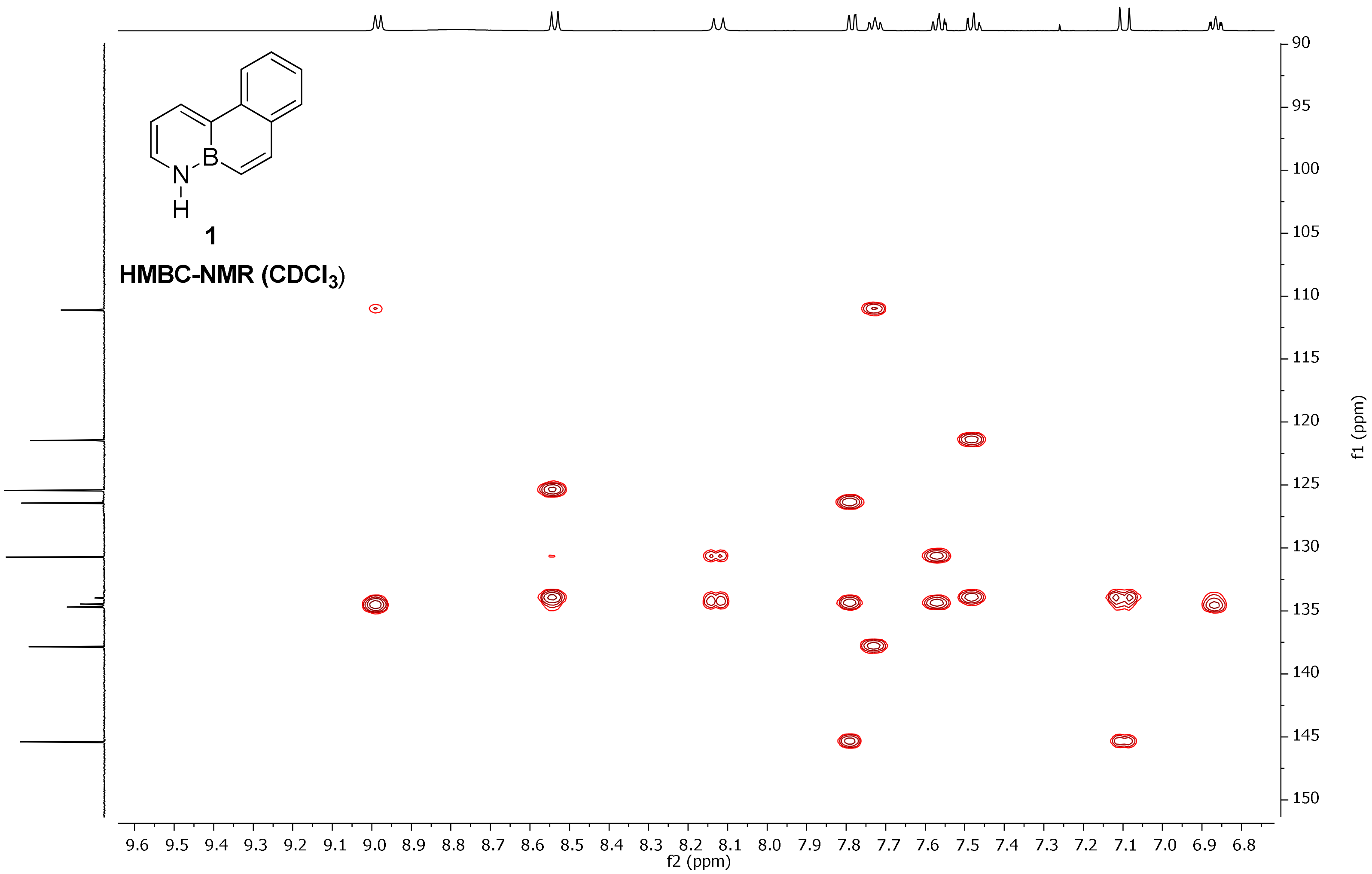

1
HMBC-NMR ($CDCl_3$)
f2 (ppm)
f1 (ppm)

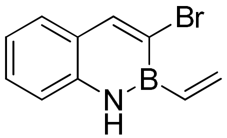

7

^{1}H-NMR (300 MHz, $CDCl_3$)

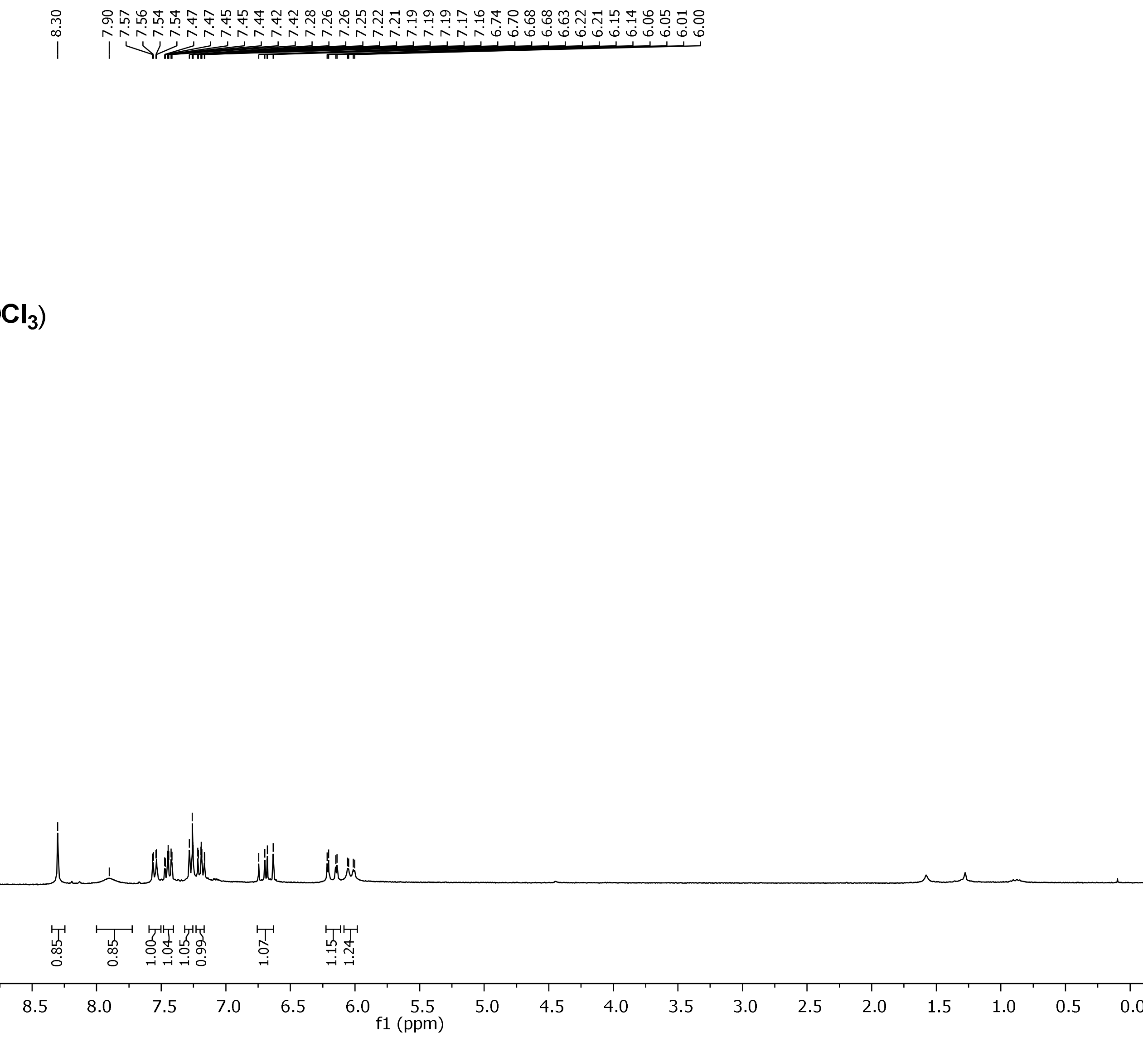

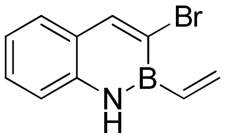

7

^{13}C-NMR (125 MHz, $CDCl_3$)

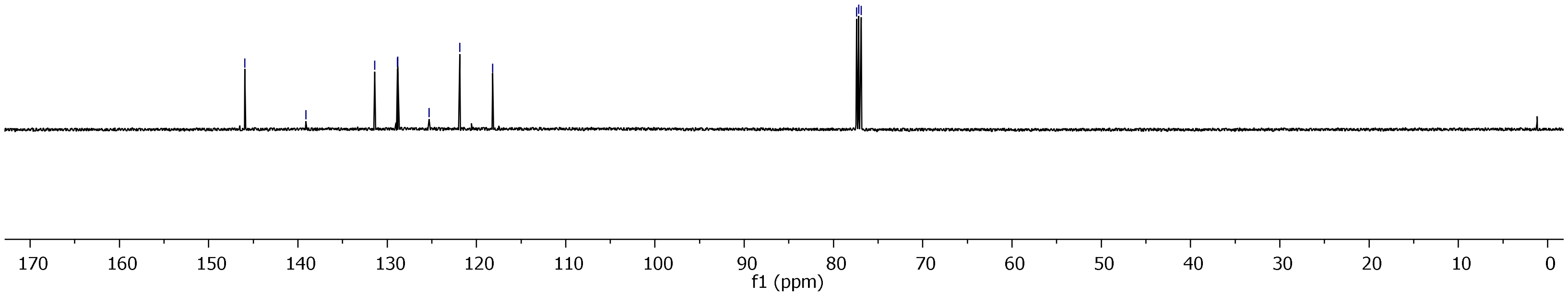

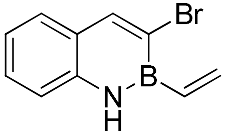

7

^{11}B-NMR (160 MHz, $CDCl_3$)

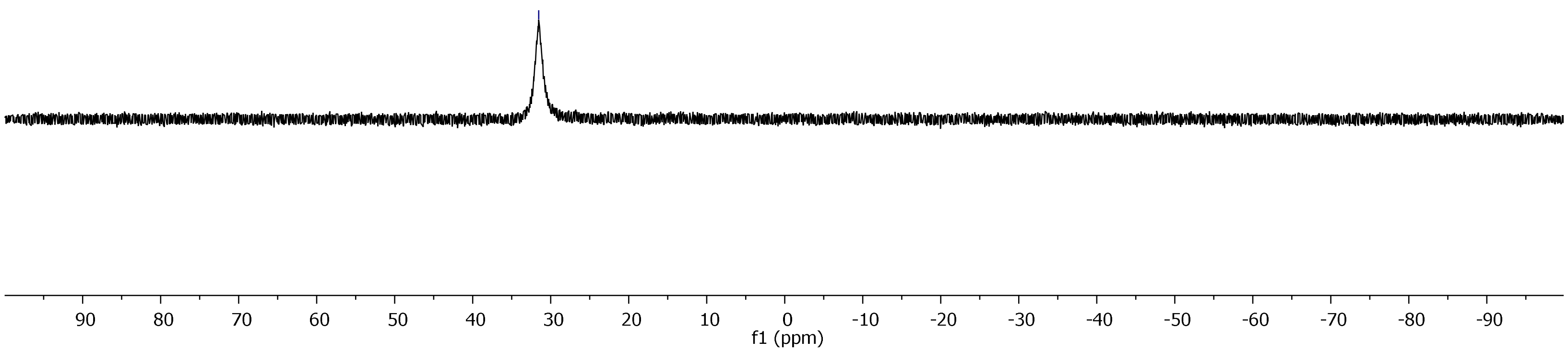

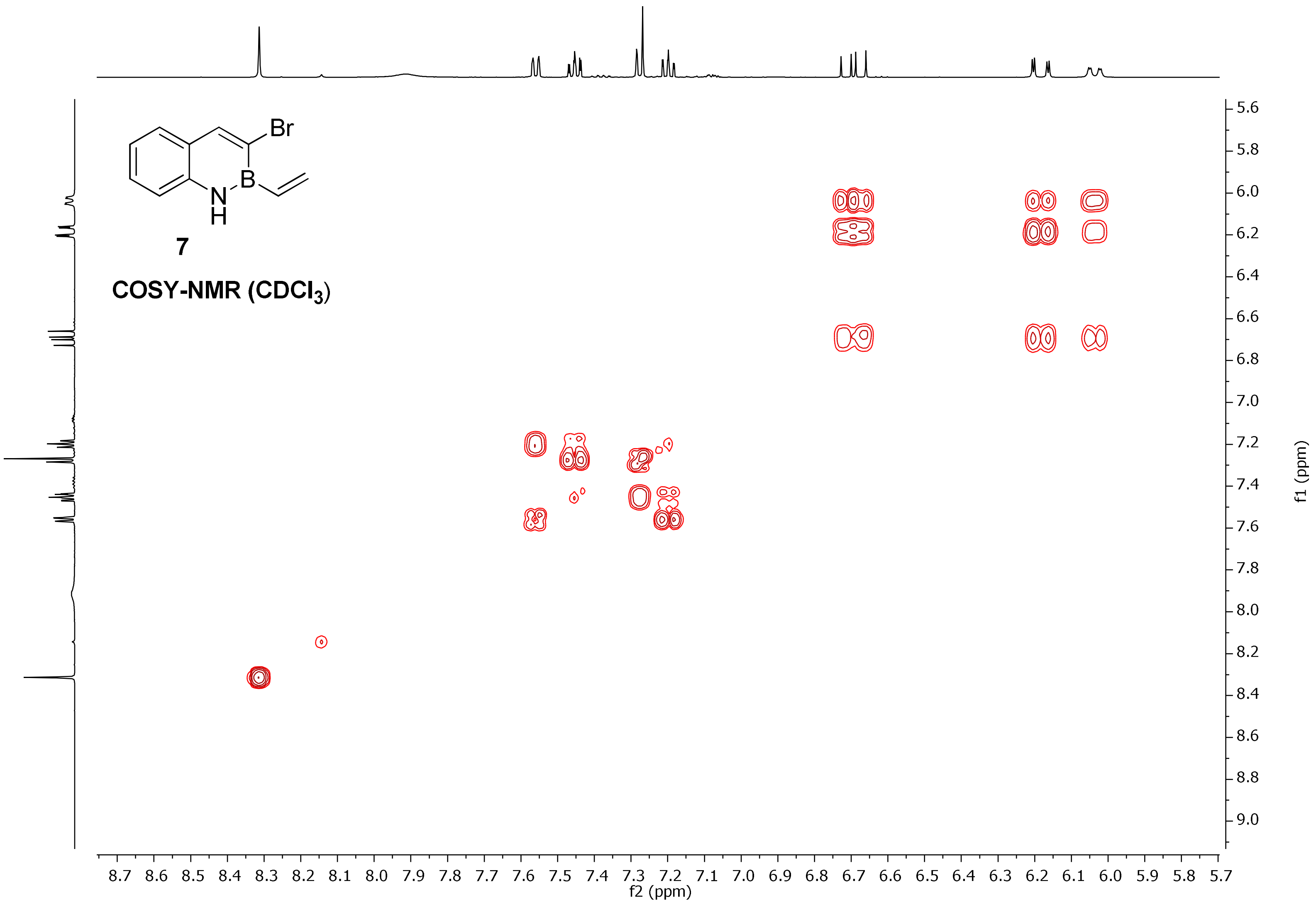
Br
B
N
H
7
COSY-NMR ($CDCl_3$)
f2 (ppm)
f1 (ppm)

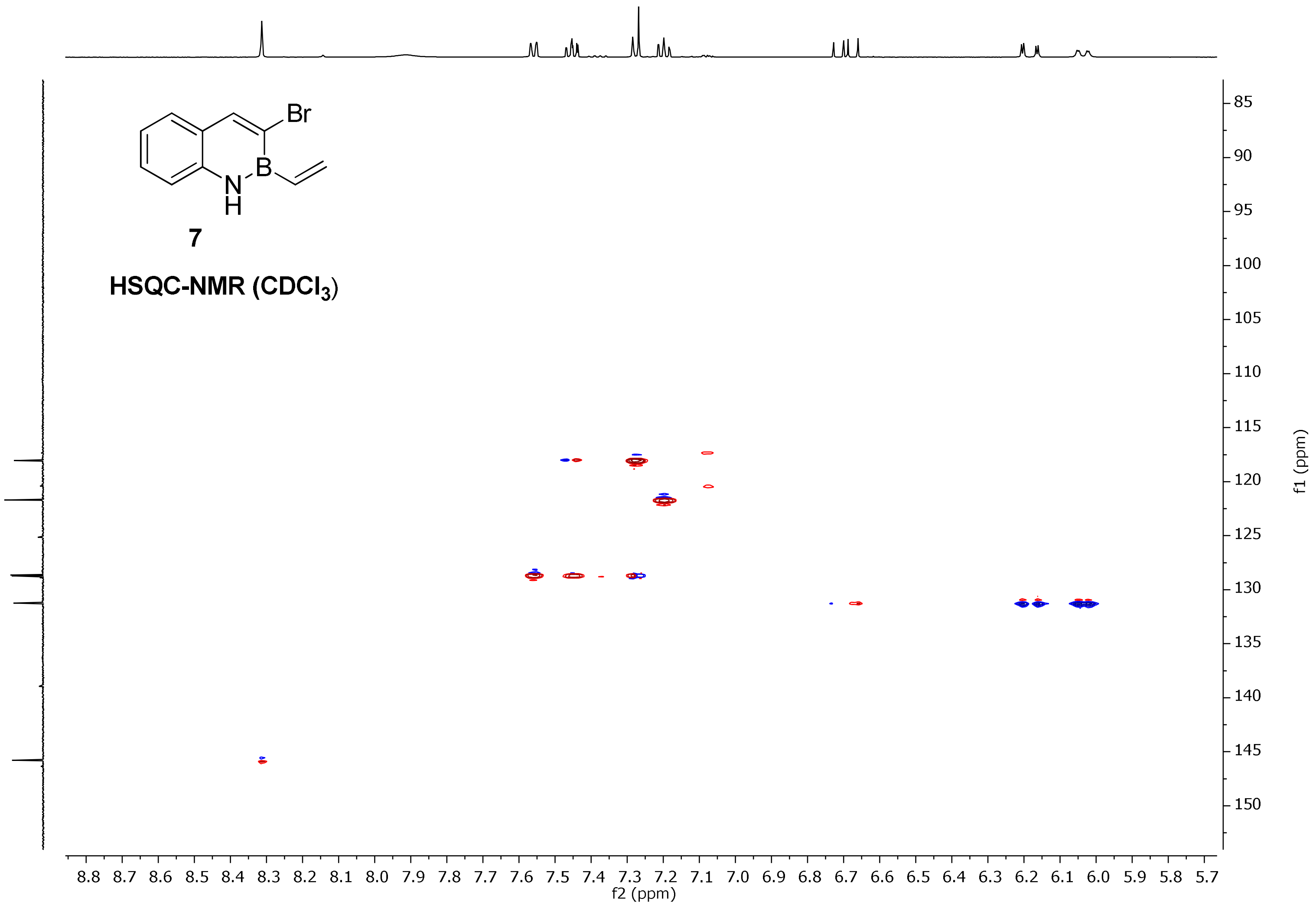
Br
B
N
H
7
HSQC-NMR (CDCl3)
85
90
95
100
105
110
115
120
125
130
135
140
145
150
f1 (ppm)
8.8 8.7 8.6 8.5 8.4 8.3 8.2 8.1 8.0 7.9 7.8 7.7 7.6 7.5 7.4 7.3 7.2 7.1 7.0 6.9 6.8 6.7 6.6 6.5 6.4 6.3 6.2 6.1 6.0 5.9 5.8 5.7
f2 (ppm)

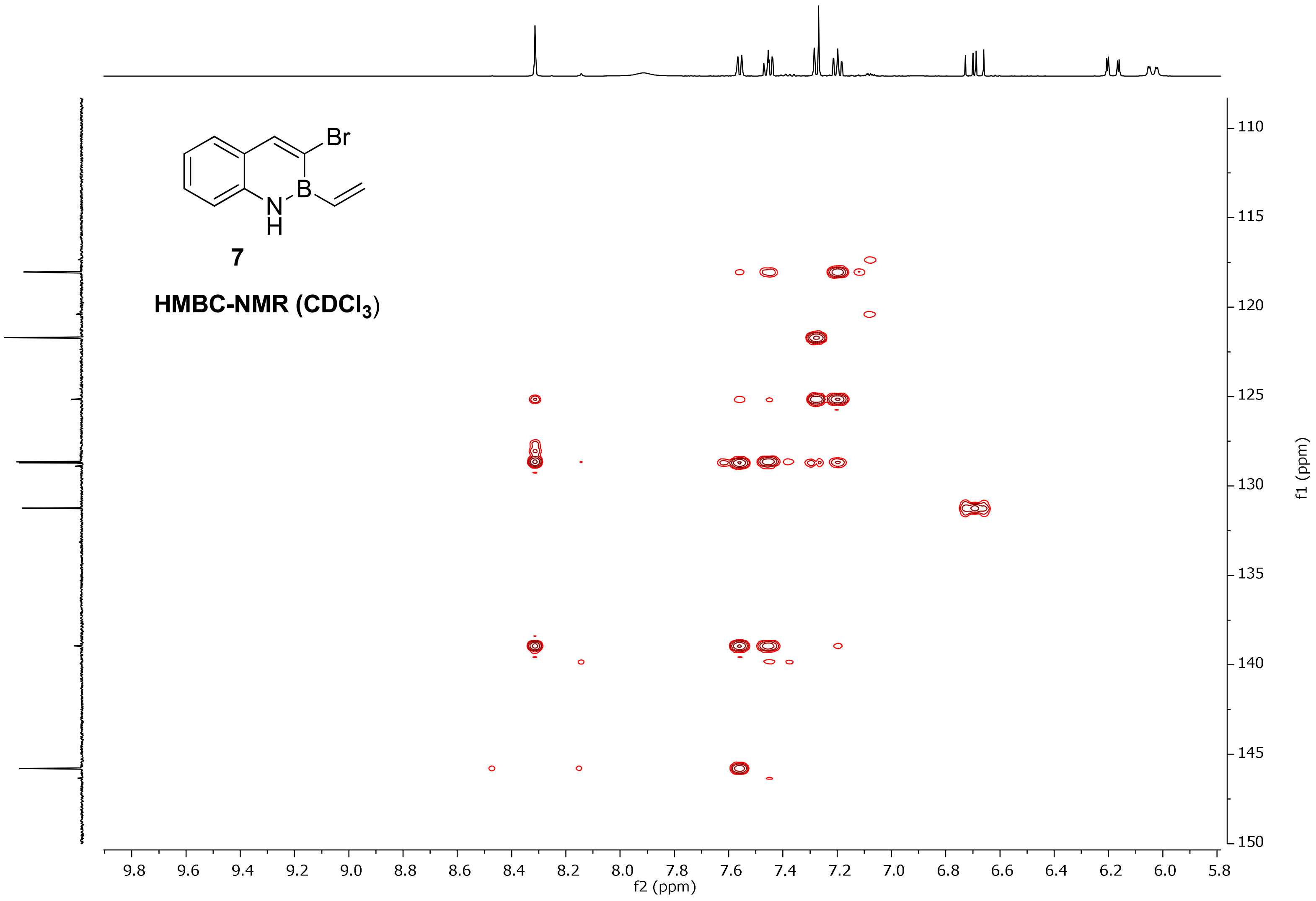
Br
B
N
H
7
HMBC-NMR (CDCl$_3$)
110
115
120
125
130
135
140
145
150
f1 (ppm)
9.8
9.6
9.4
9.2
9.0
8.8
8.6
8.4
8.2
8.0
7.8
7.6
7.4
7.2
7.0
6.8
6.6
6.4
6.2
6.0
5.8
f2 (ppm)

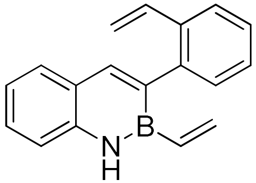

8

^{1}H-NMR (300 MHz, $CDCl_3$)

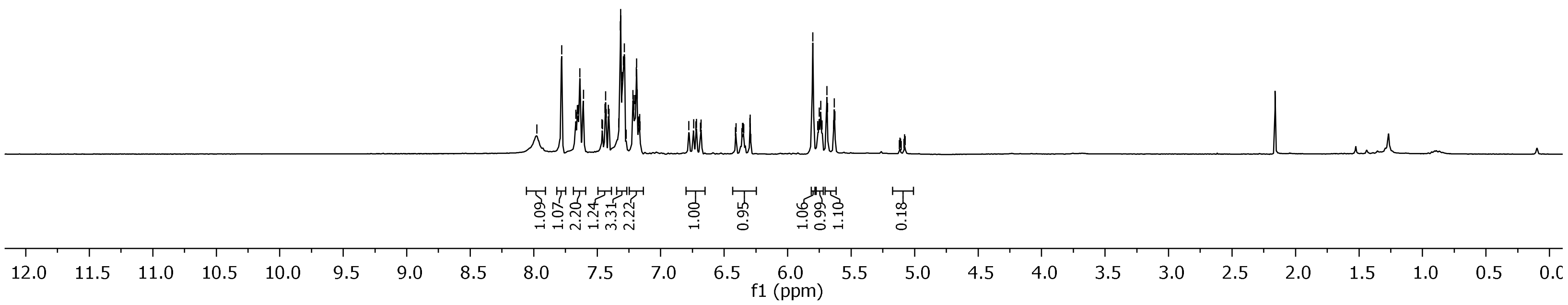

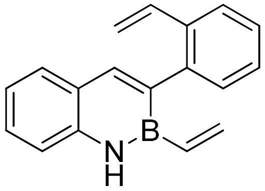

8

^{13}C-NMR (125 MHz, $CDCl_3$)

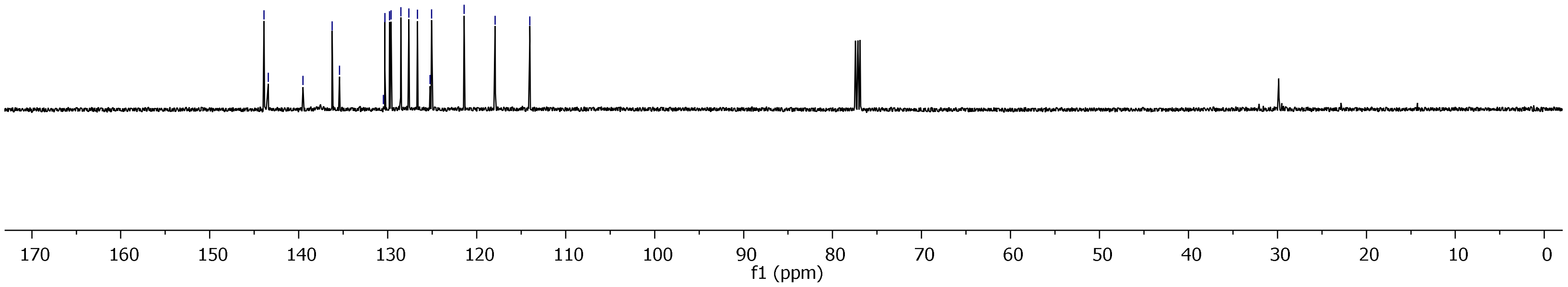

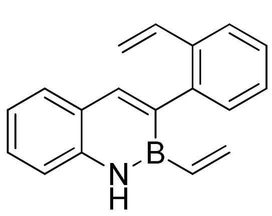

8

^{11}B-NMR (160 MHz, $CDCl_3$)

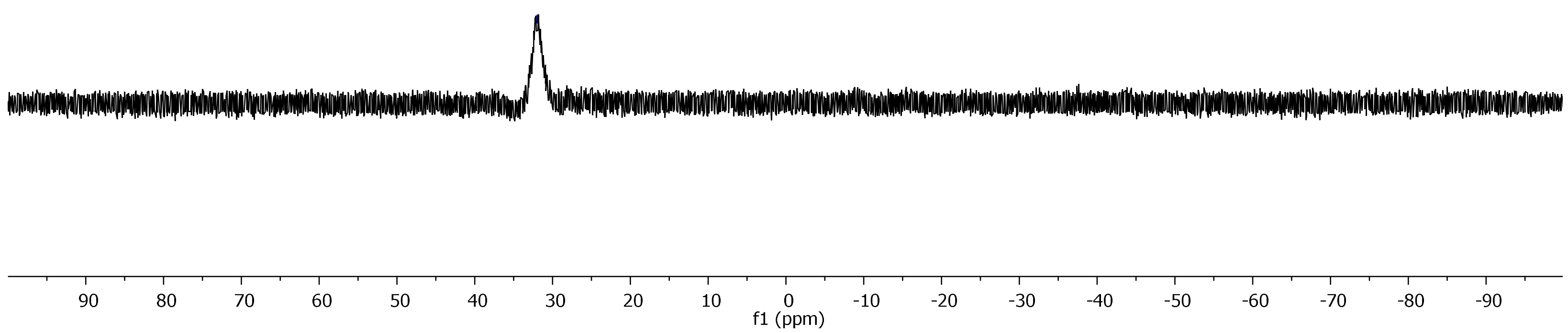

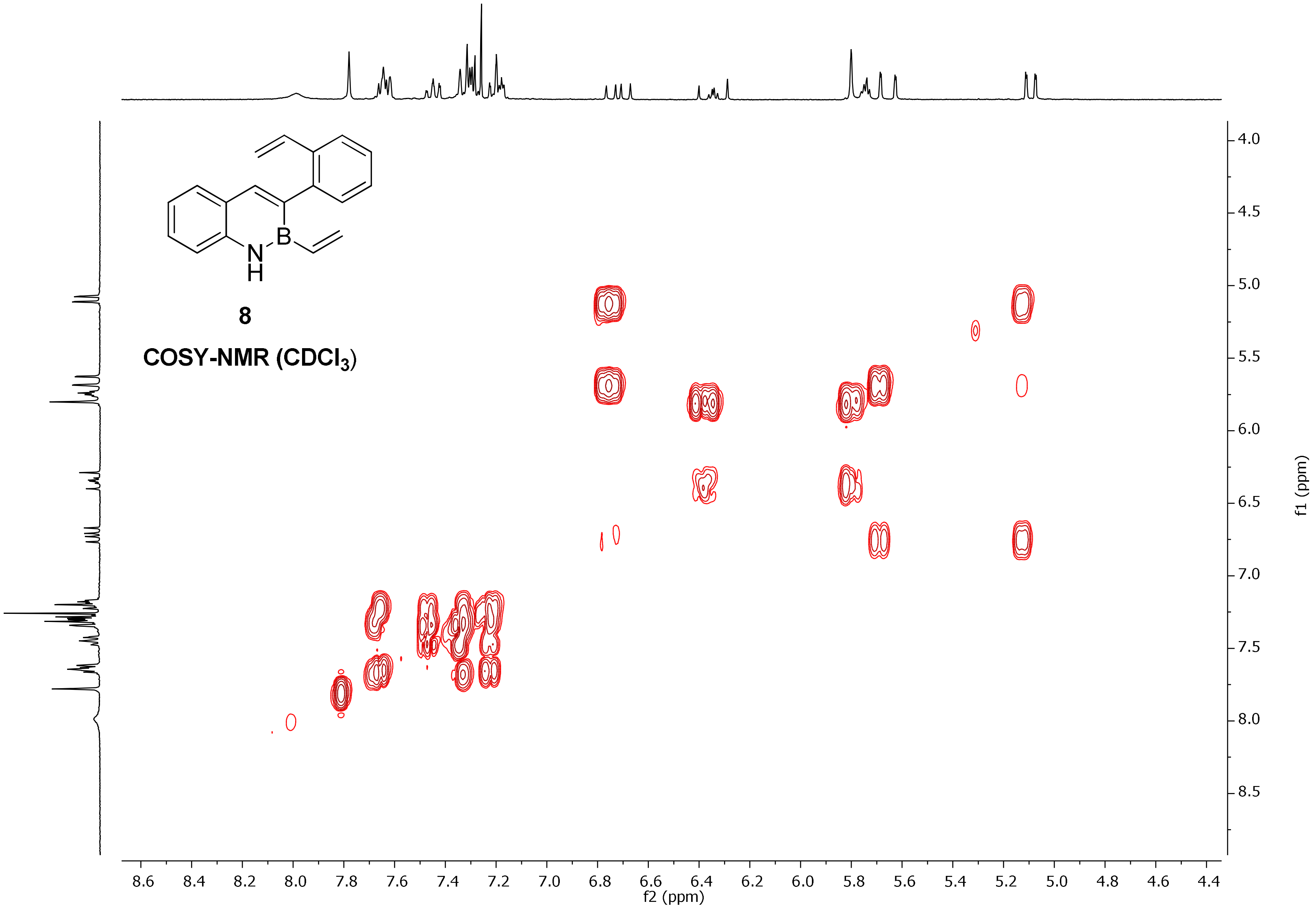

8
COSY-NMR ($CDCl_3$)
f2 (ppm)
f1 (ppm)

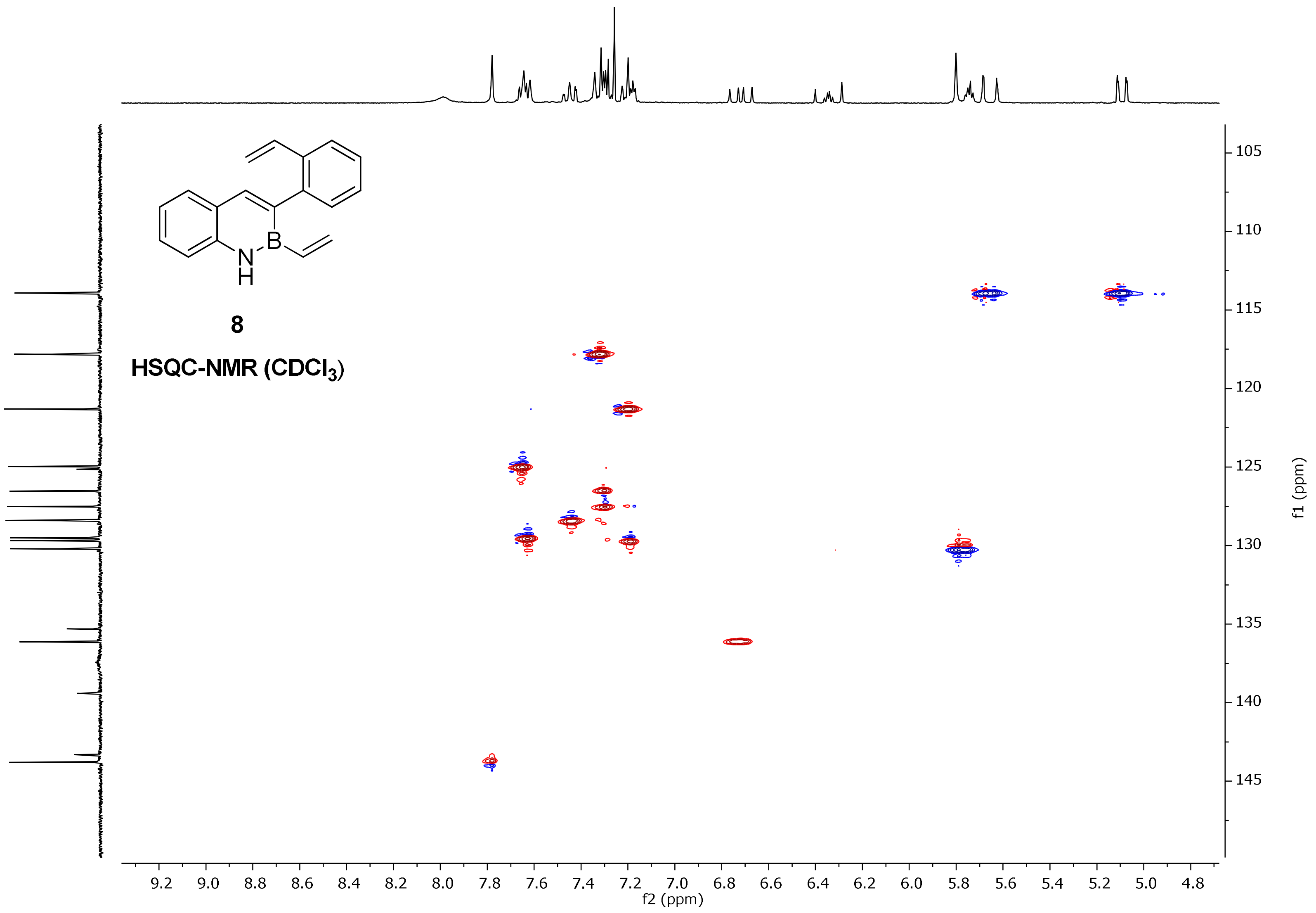

N
B
H
8
HSQC-NMR (CDCl3)
f1 (ppm)
105
110
115
120
125
130
135
140
145
9.2 9.0 8.8 8.6 8.4 8.2 8.0 7.8 7.6 7.4 7.2 7.0 6.8 6.6 6.4 6.2 6.0 5.8 5.6 5.4 5.2 5.0 4.8
f2 (ppm)

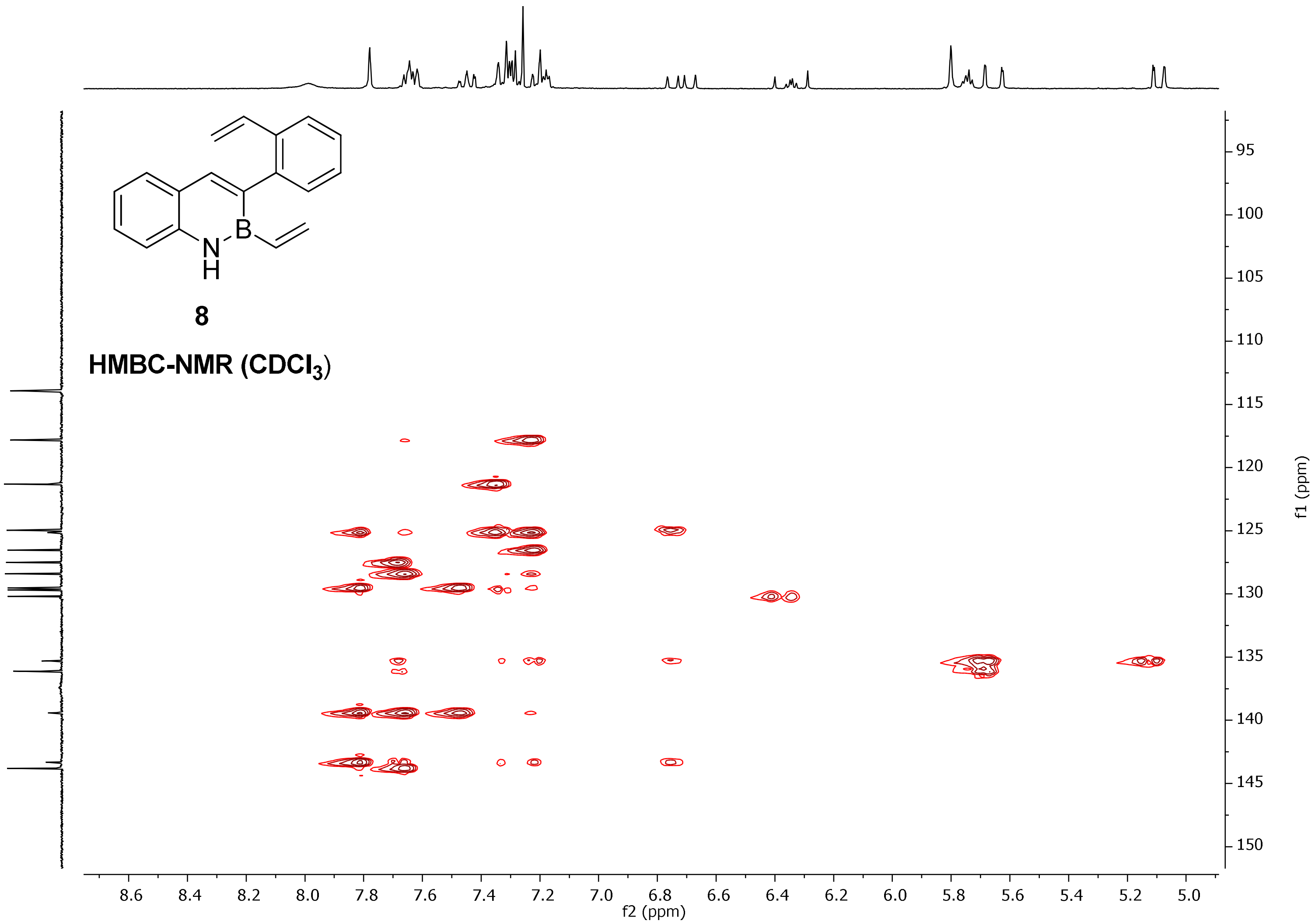

8
HMBC-NMR ($CDCl_3$)
f1 (ppm)
f2 (ppm)

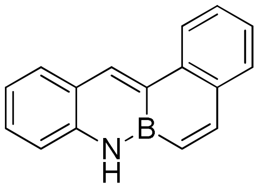

2

^{1}H-NMR (500 MHz, $CDCl_3$)

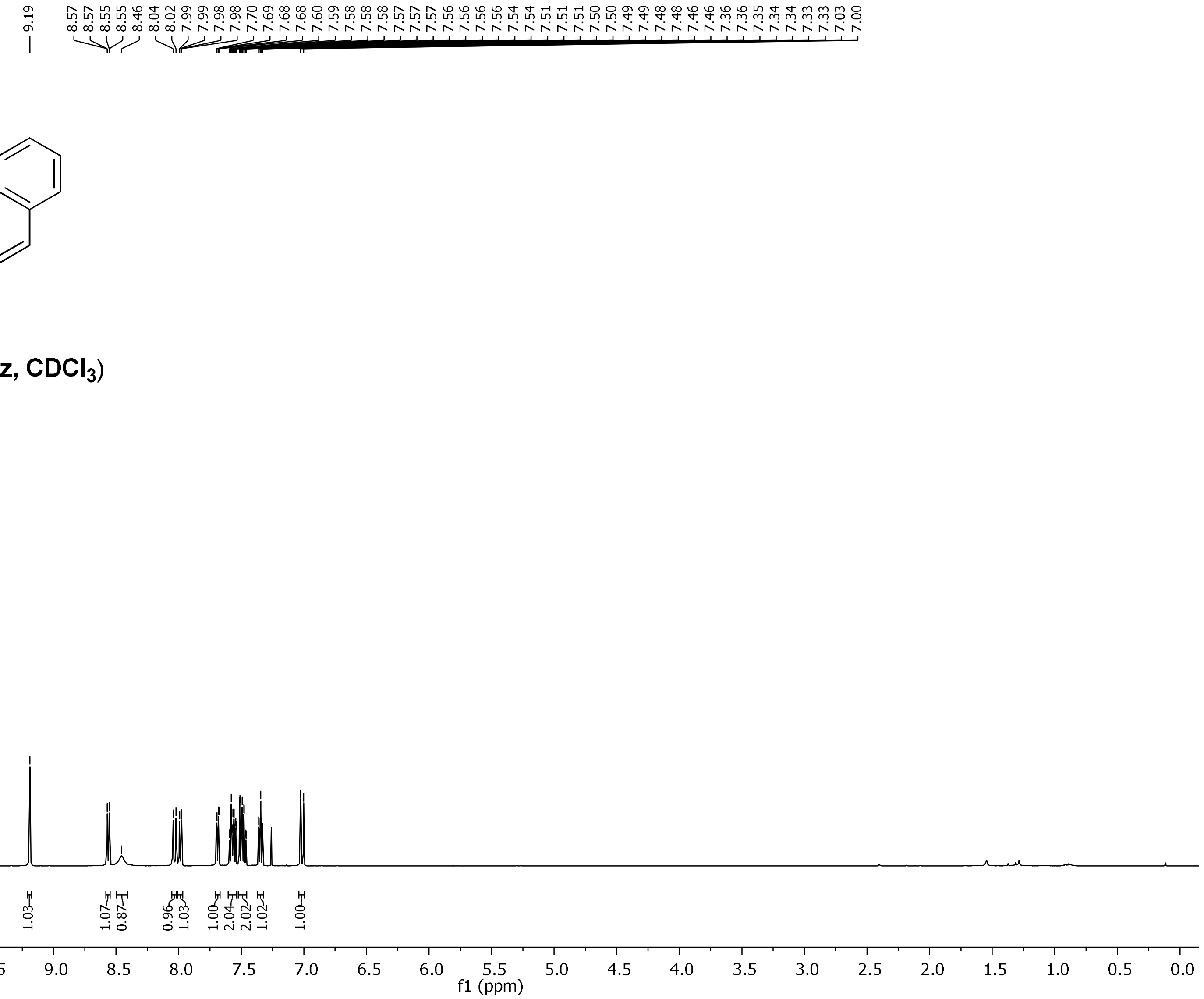

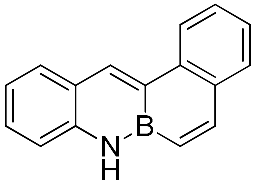

2

^{13}C-NMR (125 MHz, $CDCl_3$)

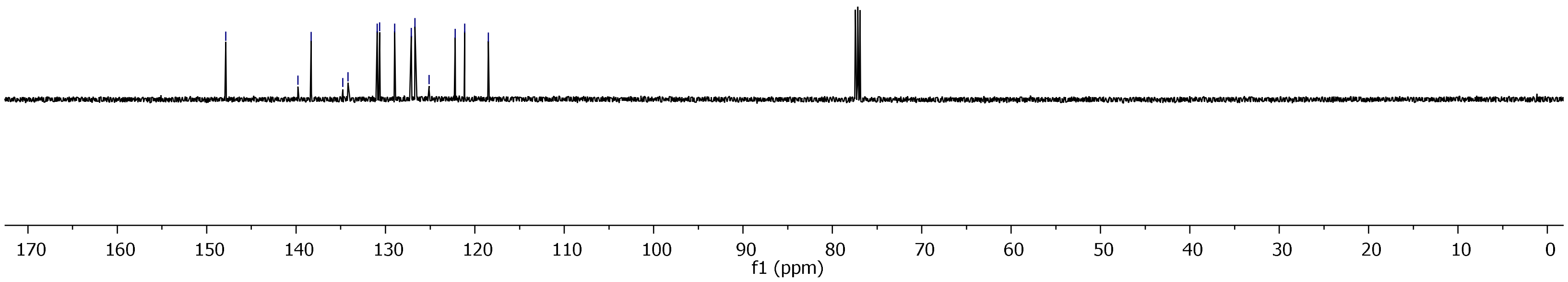

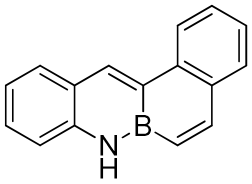

2

^{11}B-NMR (160 MHz, $CDCl_3$)

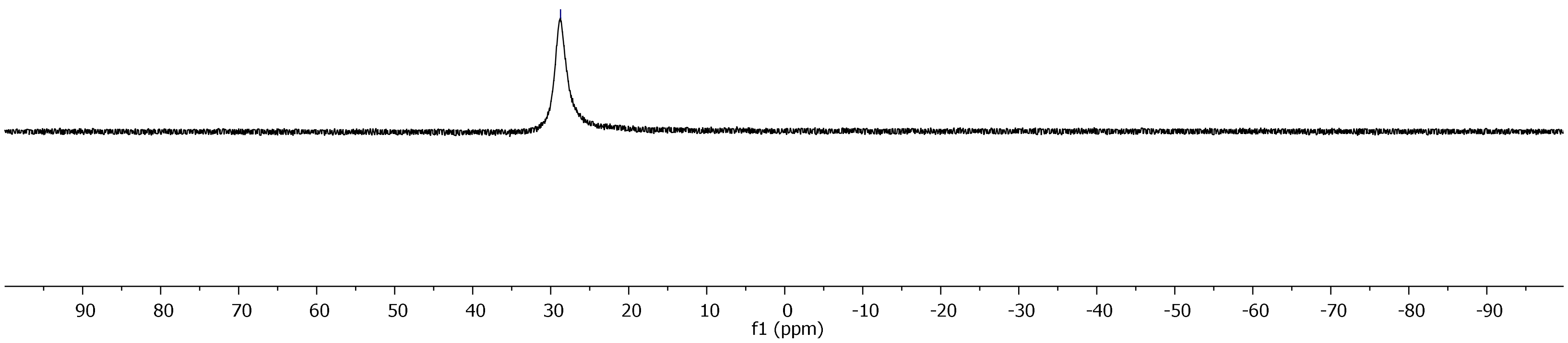

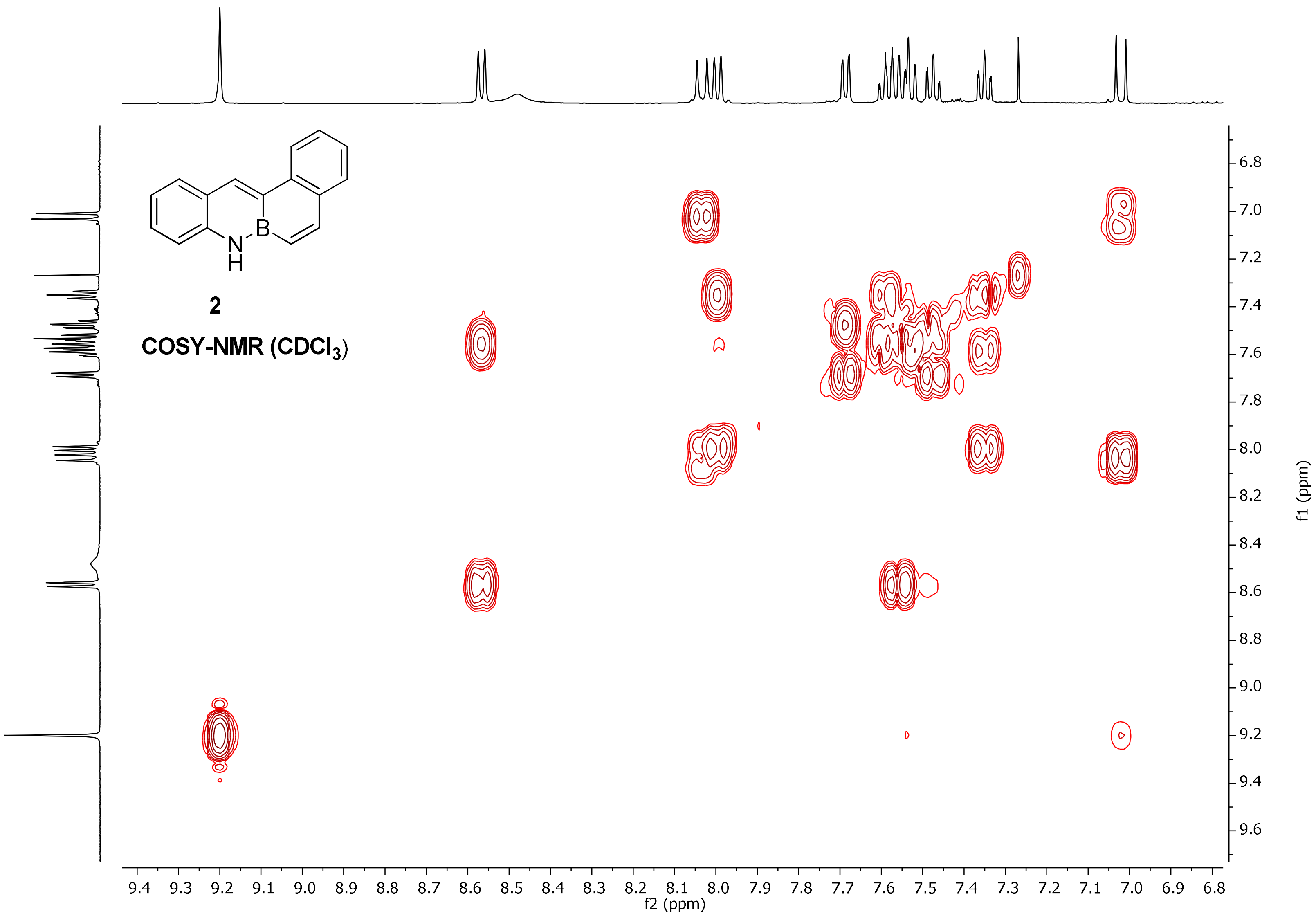

N
B
H
2
COSY-NMR ($CDCl_3$)
f2 (ppm)
f1 (ppm)

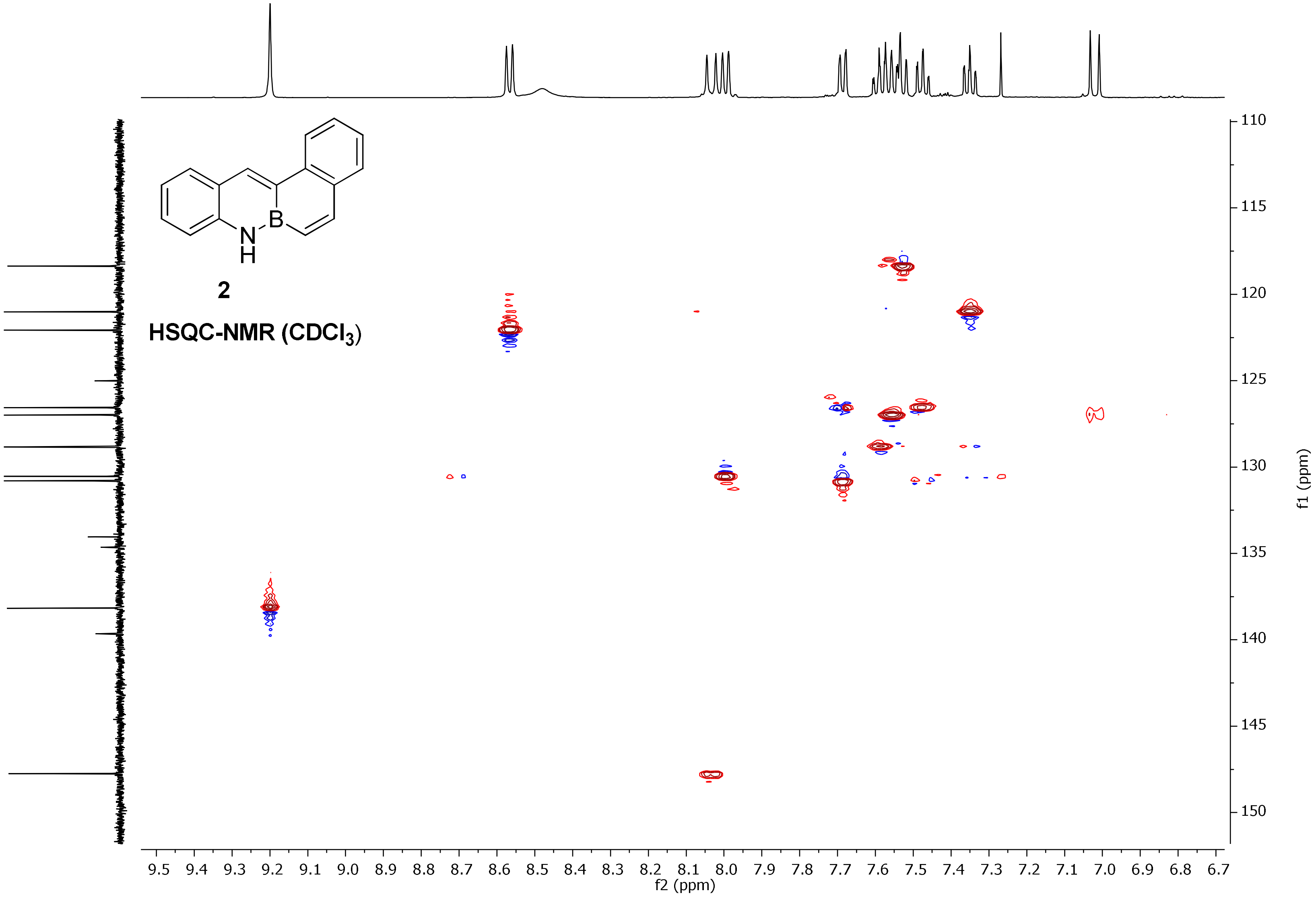

N
H
B
2
HSQC-NMR ($CDCl_3$)
f2 (ppm)
f1 (ppm)

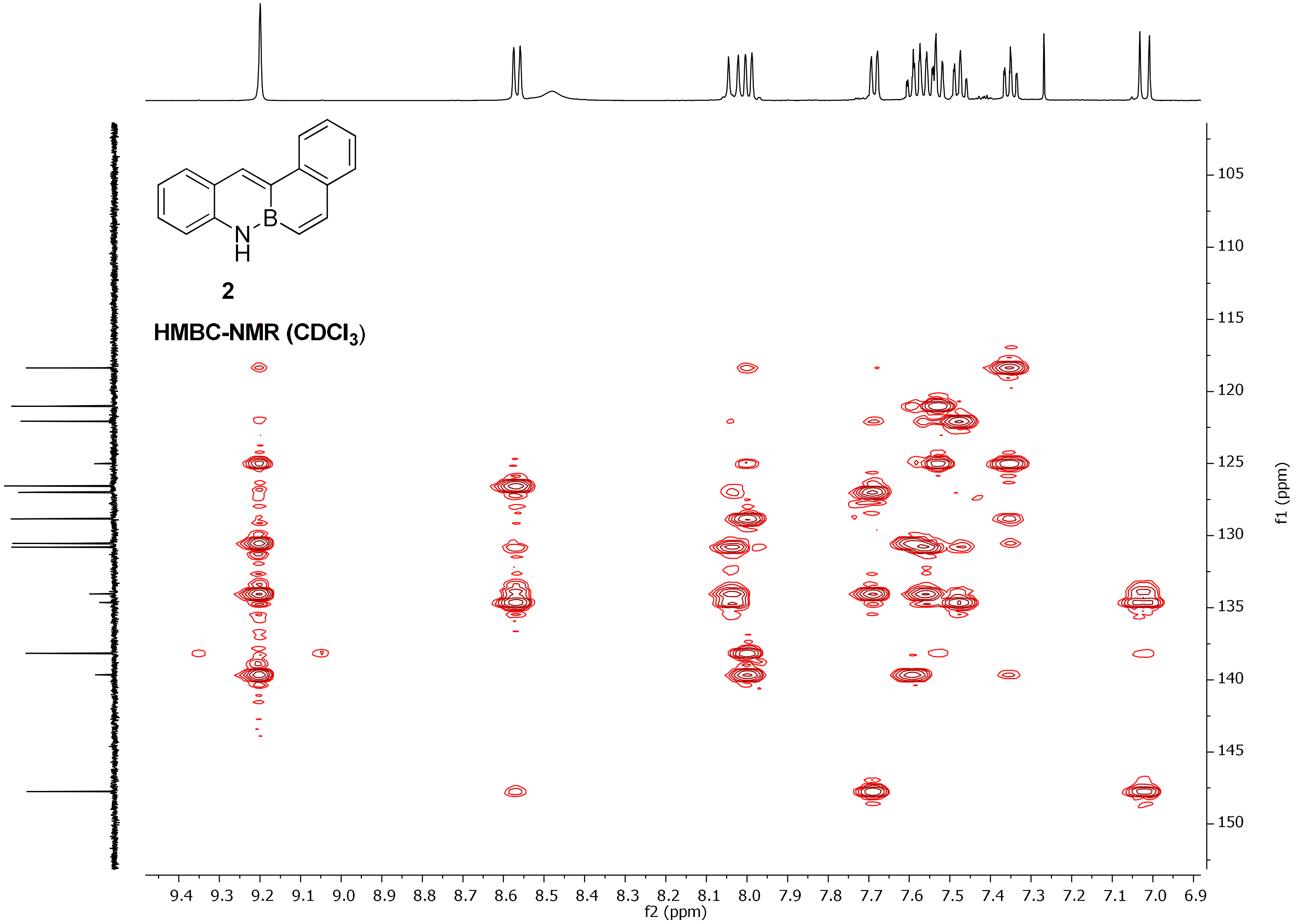
2
HMBC-NMR ($CDCl_3$)
f2 (ppm)
f1 (ppm)
N
H
B

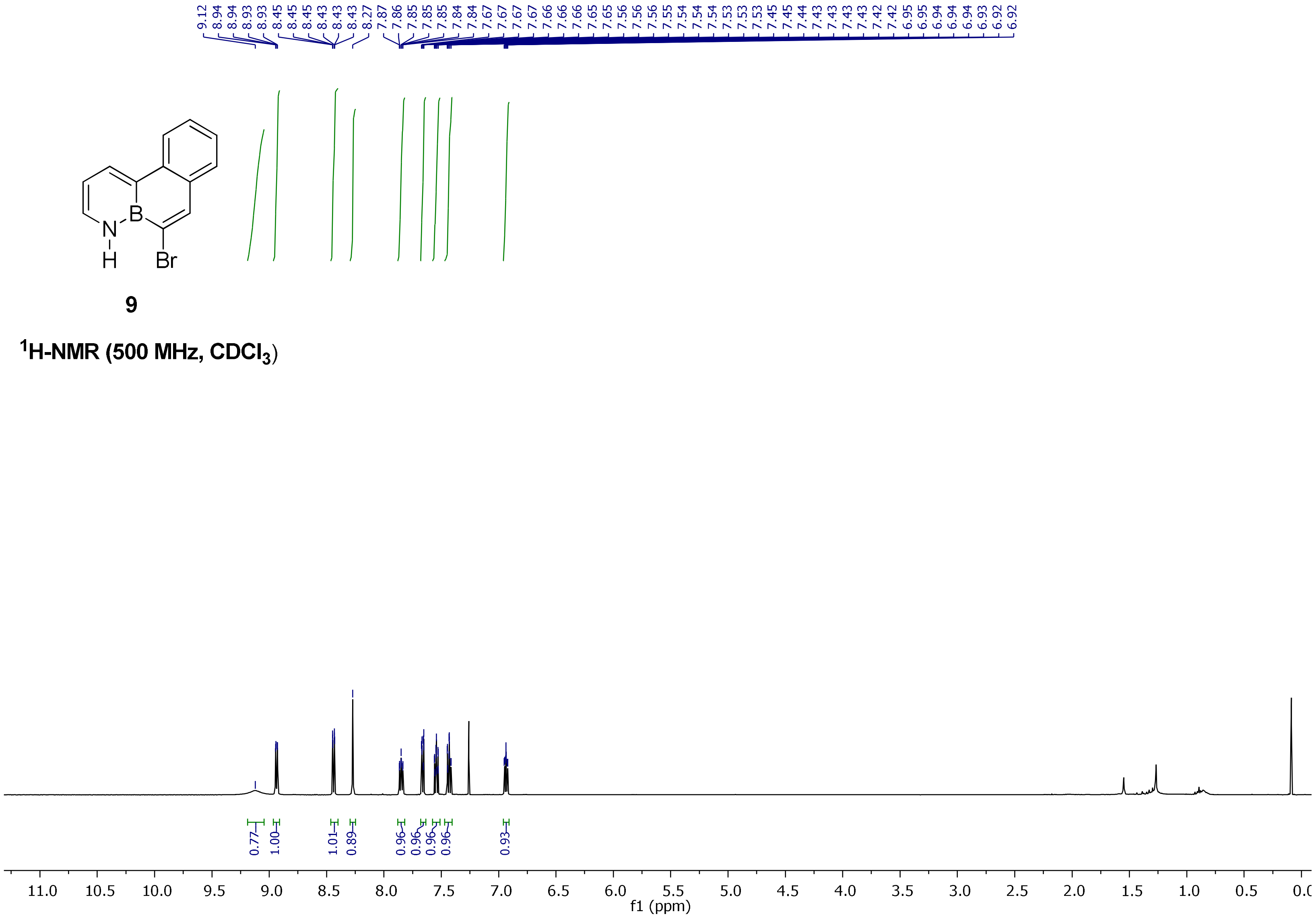
9.12 8.94 8.94 8.93 8.93 8.45 8.45 8.45 8.43 8.43 8.43 8.27 7.87 7.86 7.85 7.85 7.85 7.84 7.84 7.67 7.67 7.67 7.66 7.66 7.66 7.65 7.65 7.56 7.56 7.56 7.55 7.54 7.54 7.54 7.53 7.53 7.53 7.45 7.45 7.44 7.43 7.43 7.43 7.42 7.42 6.95 6.95 6.94 6.94 6.94 6.93 6.92 6.92
N
B
H
Br
9
^{1}H-NMR (500 MHz, CDCl$_3$)
0.77 1.00 1.01 0.89 0.96 0.96 0.96 0.96 0.93
11.0 10.5 10.0 9.5 9.0 8.5 8.0 7.5 7.0 6.5 6.0 5.5 5.0 4.5 4.0 3.5 3.0 2.5 2.0 1.5 1.0 0.5 0.0
f1 (ppm)

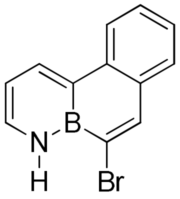

9

^{13}C-NMR (125 MHz, $CDCl_3$)

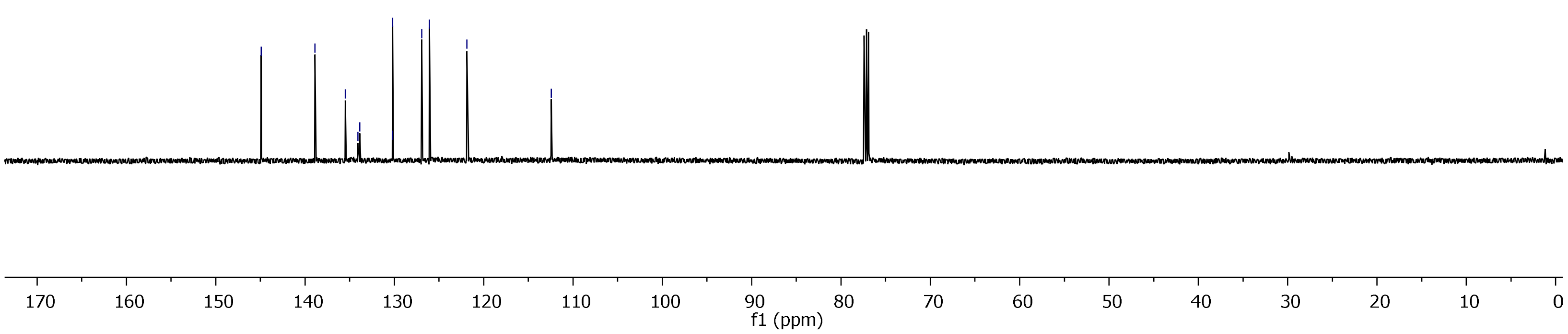

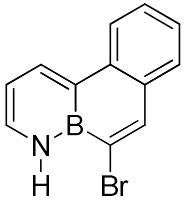

9

^{11}B-NMR (160 MHz, $CDCl_3$)

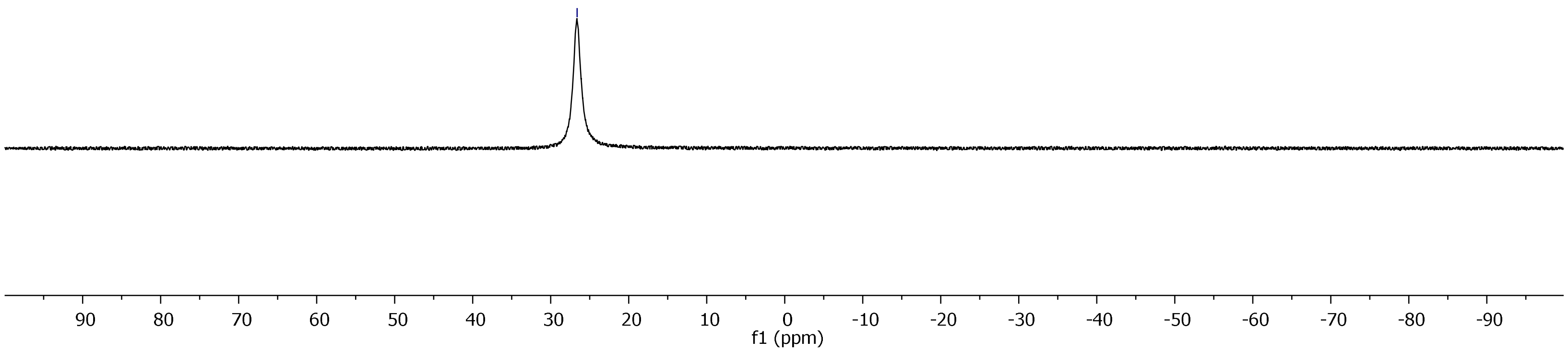

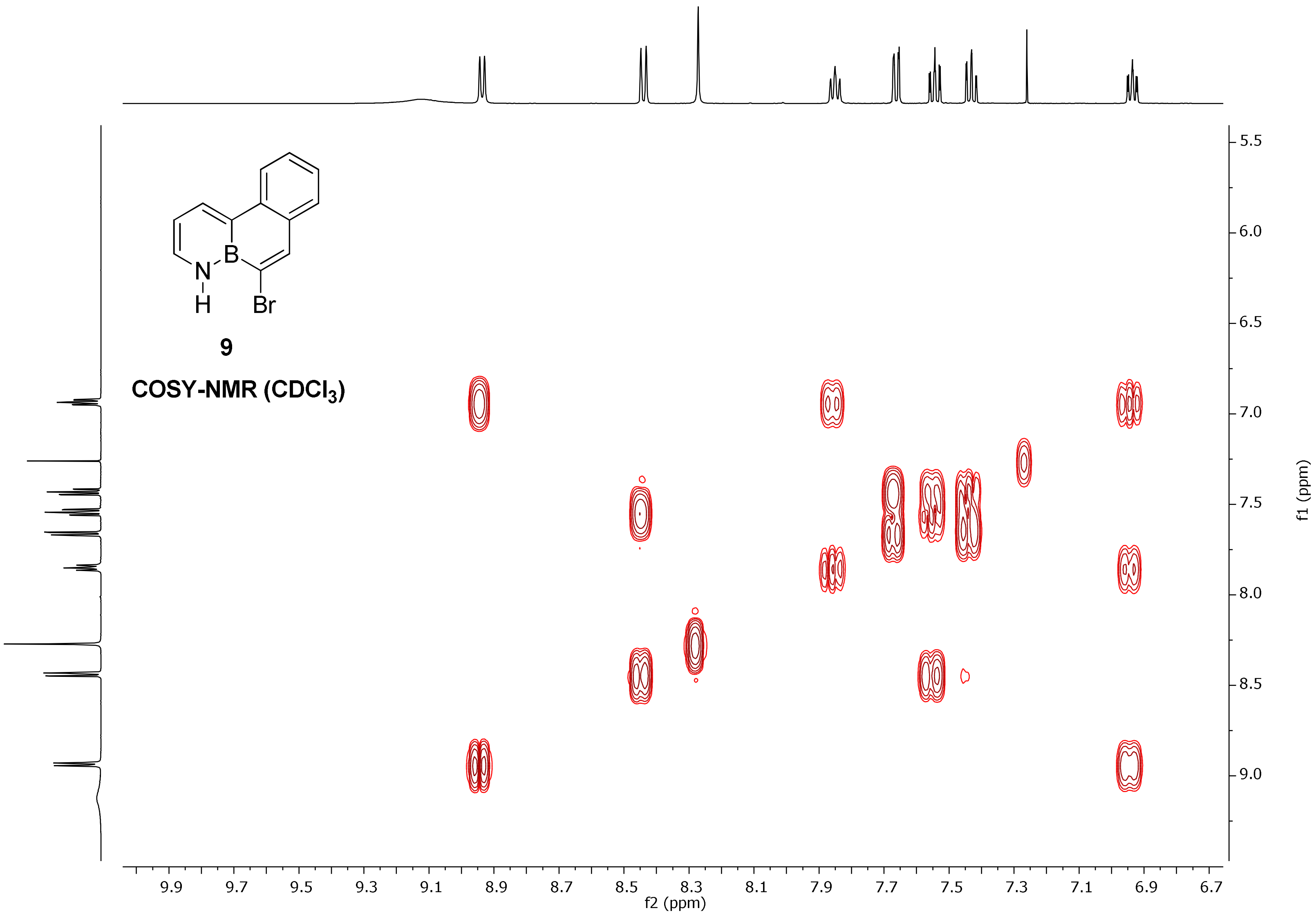
B
N
H
Br
9
COSY-NMR ($CDCl_3$)
9.9 9.7 9.5 9.3 9.1 8.9 8.7 8.5 8.3 8.1 7.9 7.7 7.5 7.3 7.1 6.9 6.7
f2 (ppm)
5.5 6.0 6.5 7.0 7.5 8.0 8.5 9.0
f1 (ppm)

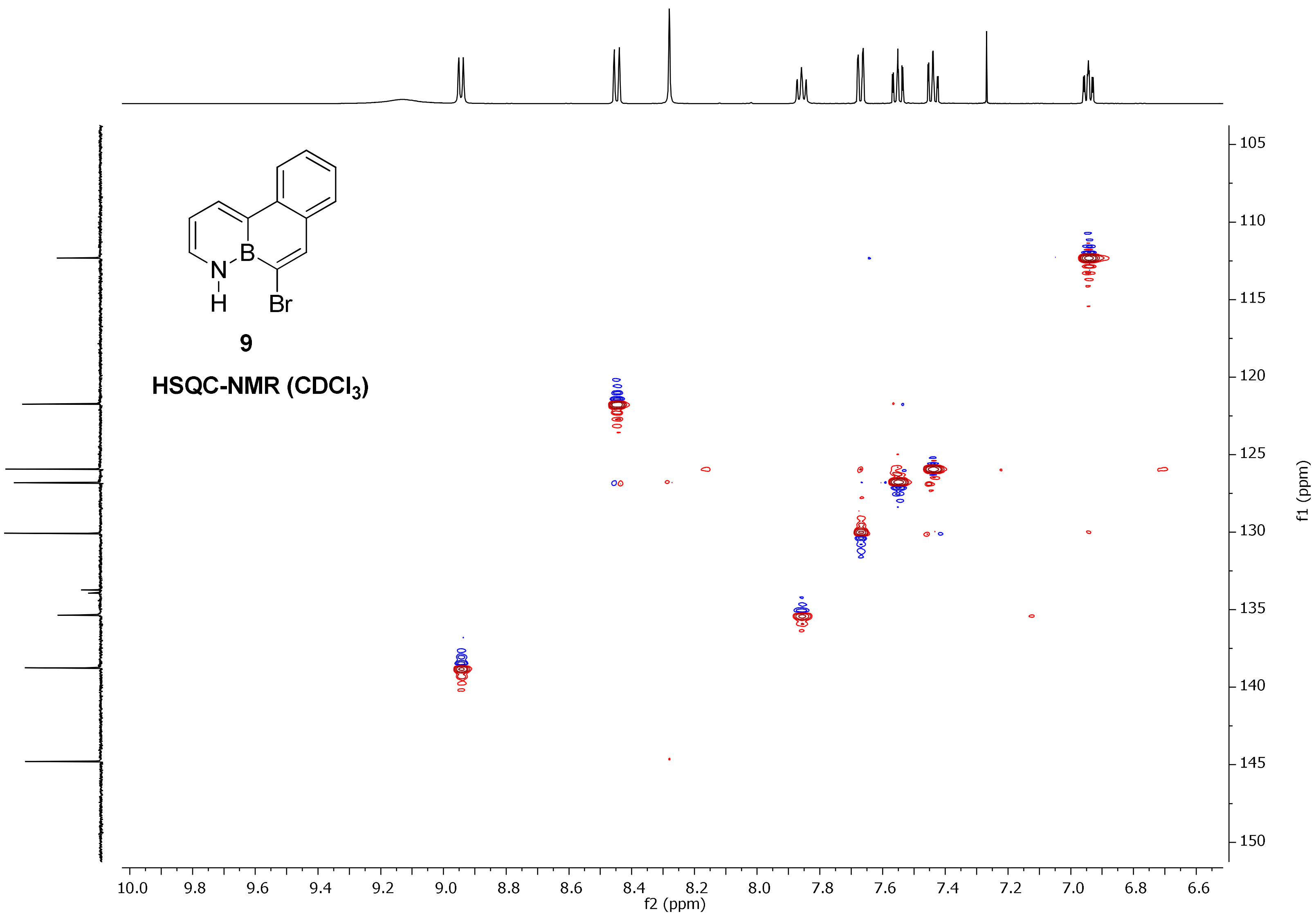
9
HSQC-NMR (CDCl3)
105
110
115
120
125
130
135
140
145
150
f1 (ppm)
10.0
9.8
9.6
9.4
9.2
9.0
8.8
8.6
8.4
8.2
8.0
7.8
7.6
7.4
7.2
7.0
6.8
6.6
f2 (ppm)
N
B
H
Br

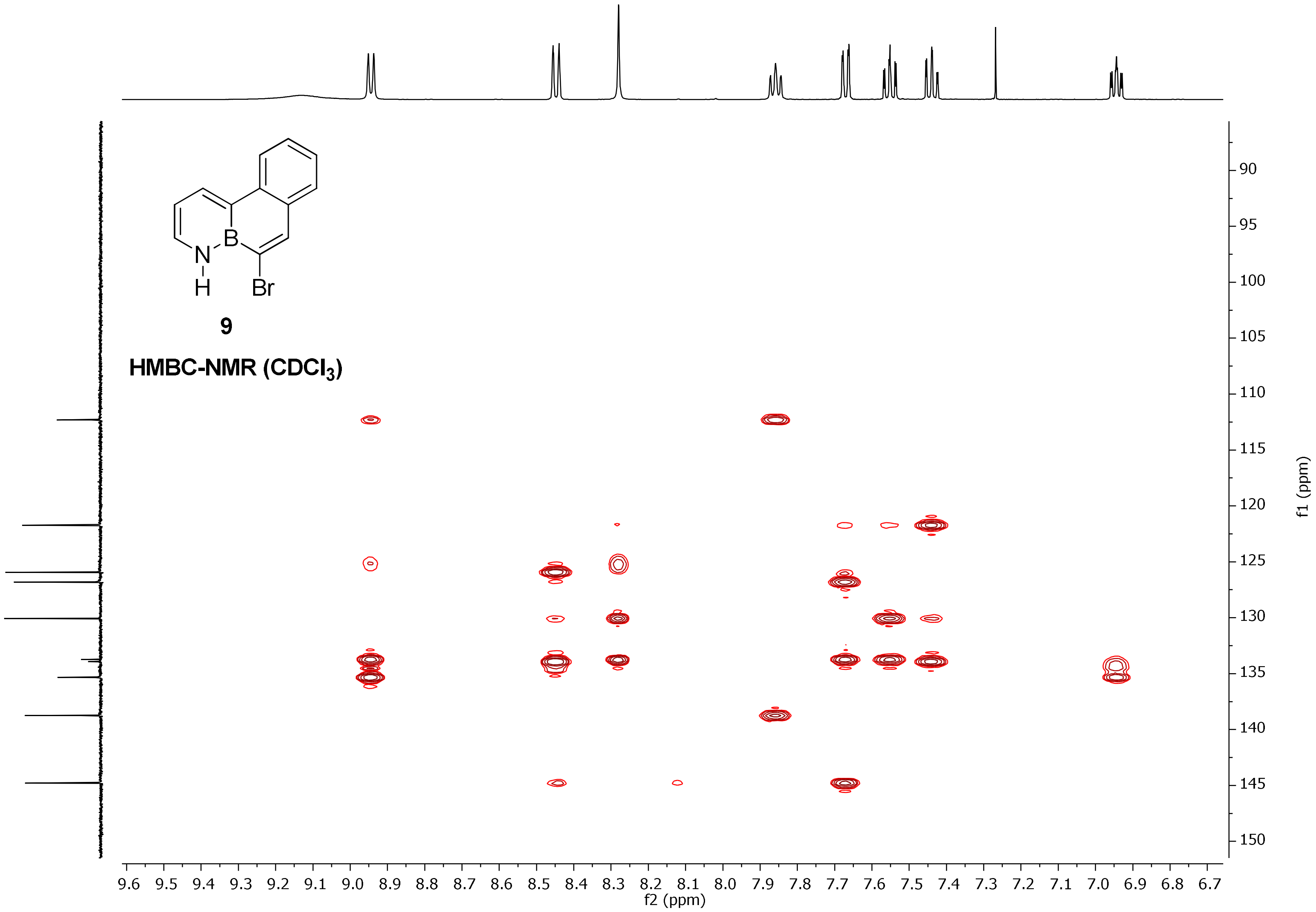

9
HMBC-NMR (CDCl3)
f2 (ppm)
f1 (ppm)

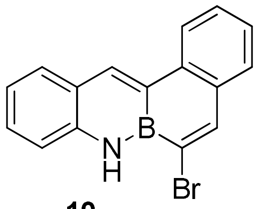

10

^{1}H-NMR (500 MHz, CDCl$_3$)

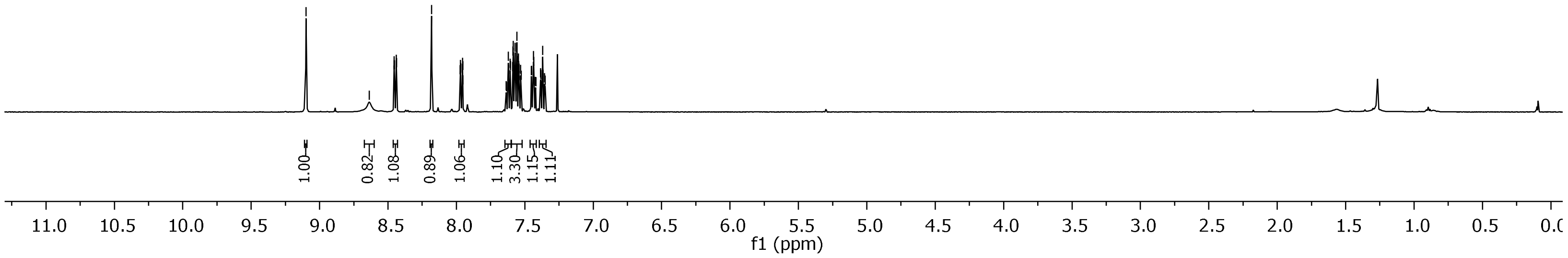

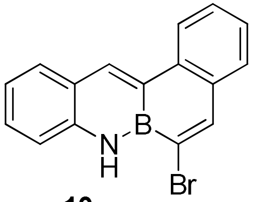

10

^{13}C-NMR (125 MHz, $CDCl_3$)

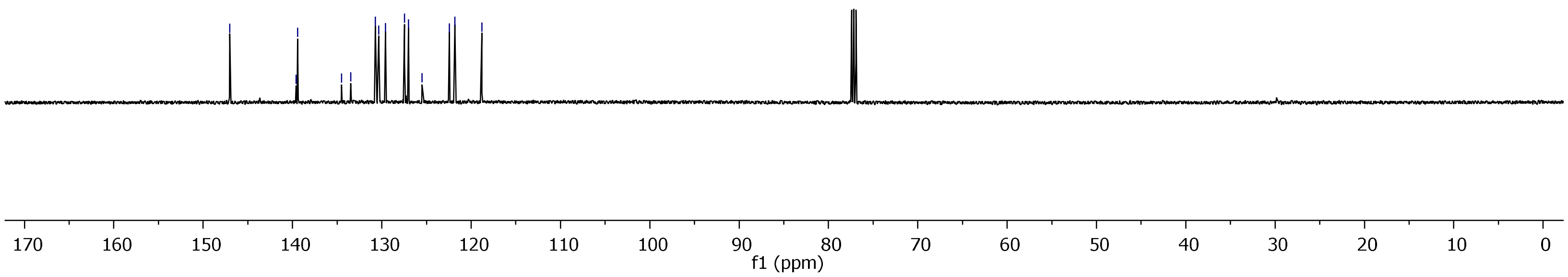

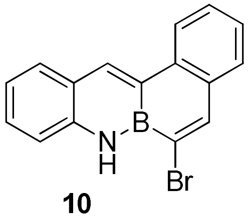

10

^{11}B-NMR (160 MHz, $CDCl_3$)

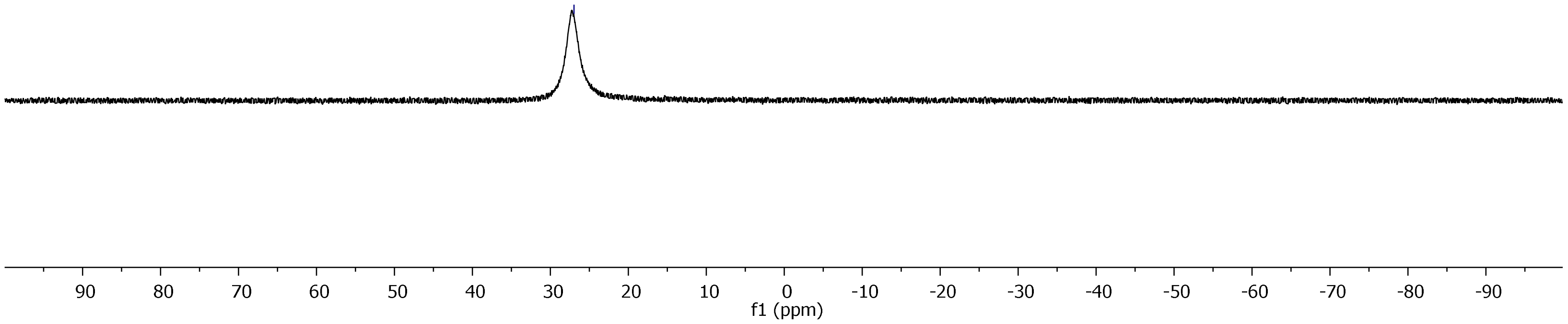

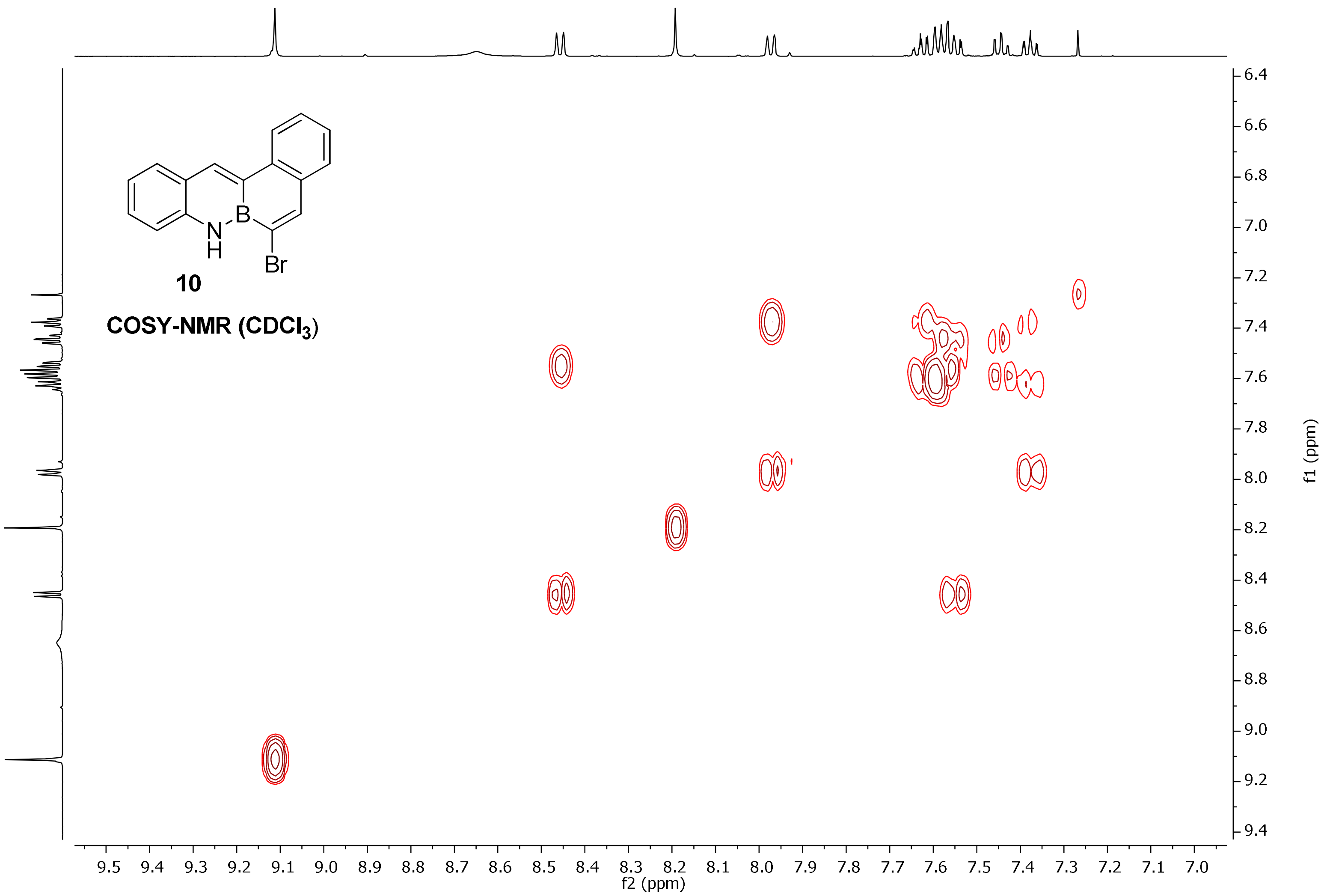
10
COSY-NMR (CDCl3)
f2 (ppm)
f1 (ppm)

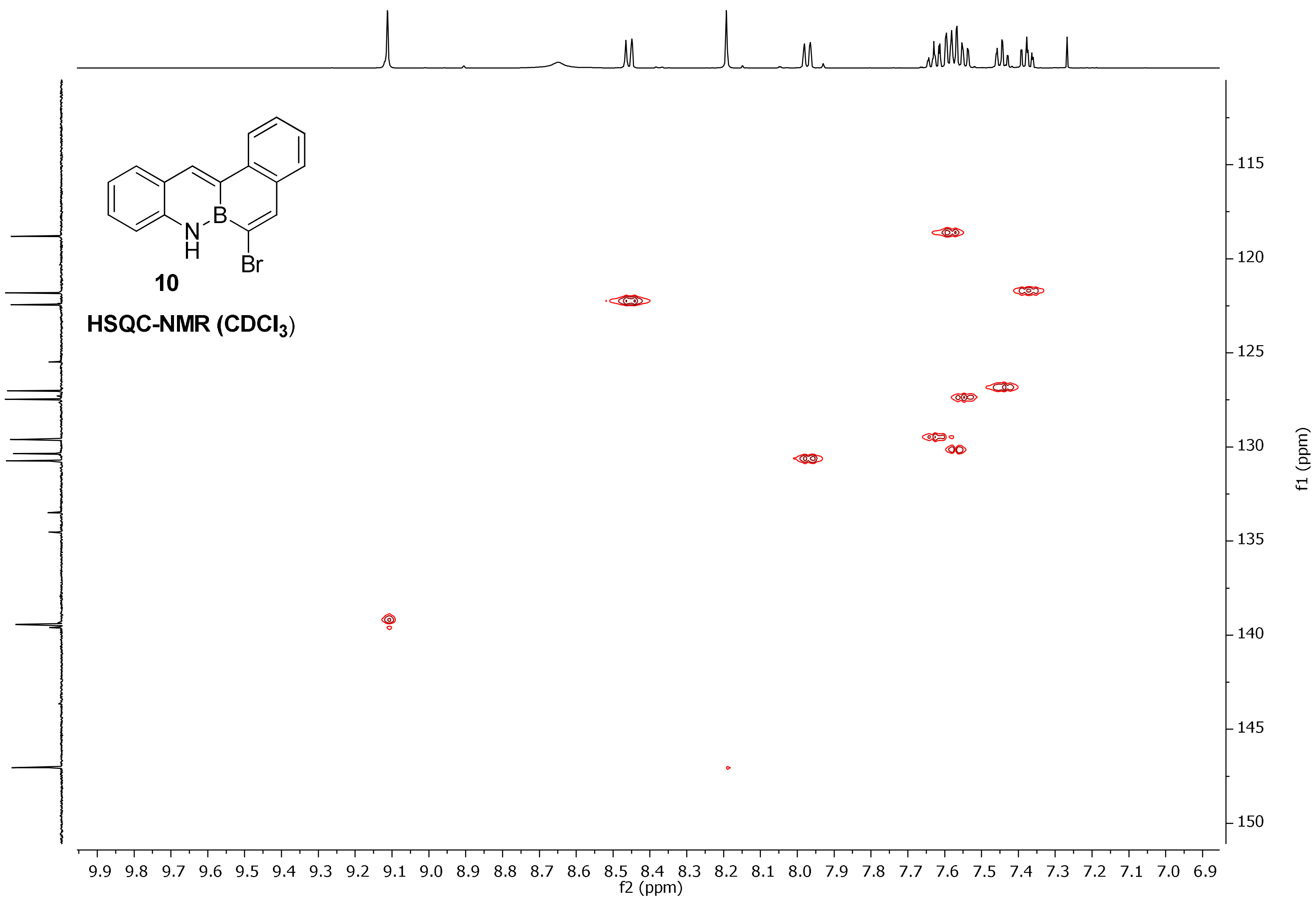

N
H
B
Br
10
HSQC-NMR ($CDCl_3$)
f2 (ppm)
f1 (ppm)

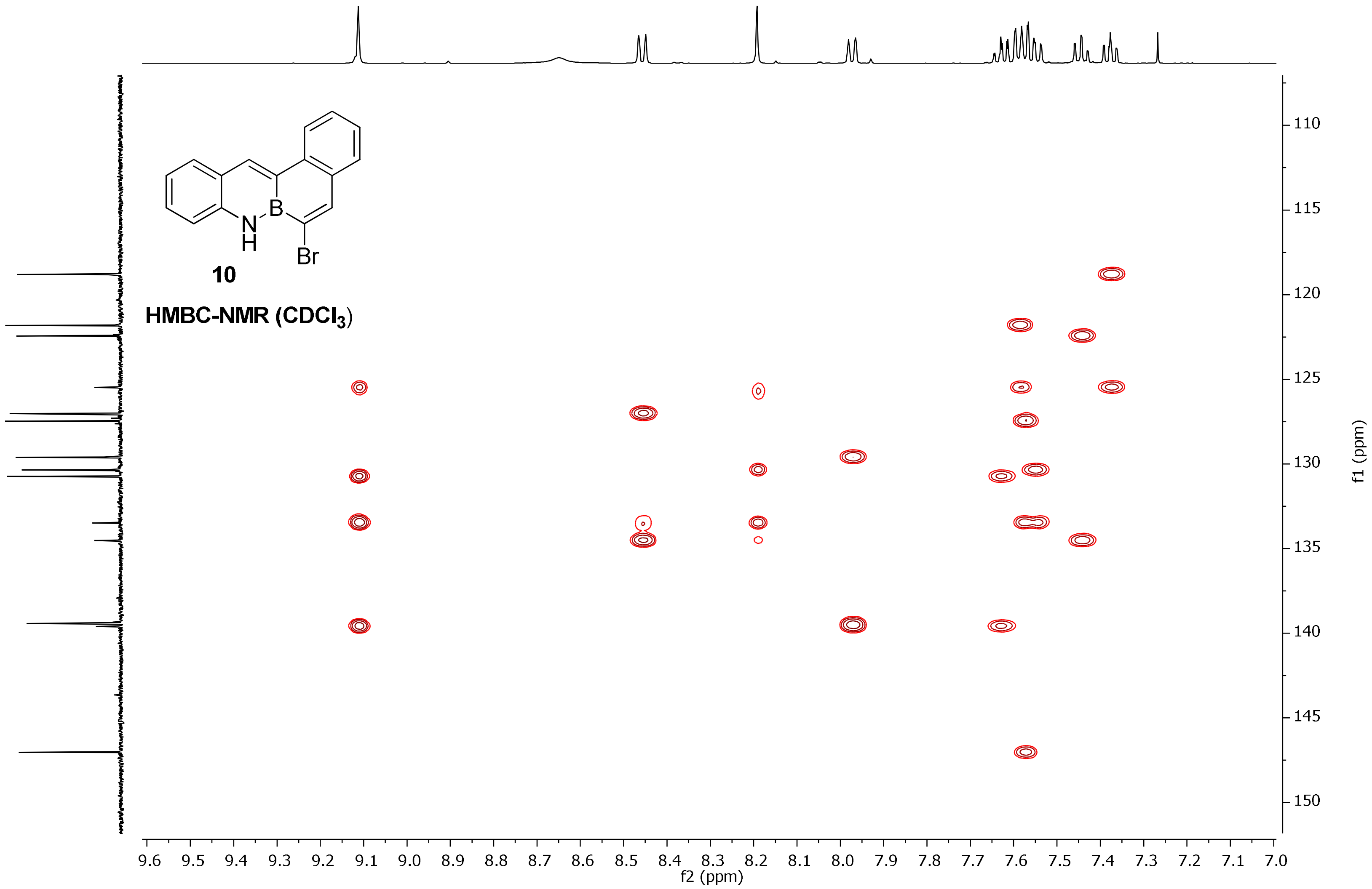
10
HMBC-NMR ($CDCl_3$)
N
H
B
Br
f1 (ppm)
f2 (ppm)
110
115
120
125
130
135
140
145
150
9.6 9.5 9.4 9.3 9.2 9.1 9.0 8.9 8.8 8.7 8.6 8.5 8.4 8.3 8.2 8.1 8.0 7.9 7.8 7.7 7.6 7.5 7.4 7.3 7.2 7.1 7.0

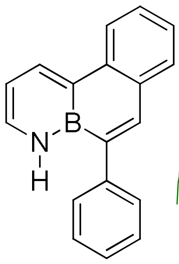

11

^{1}H-NMR (500 MHz, CDCl$_3$)

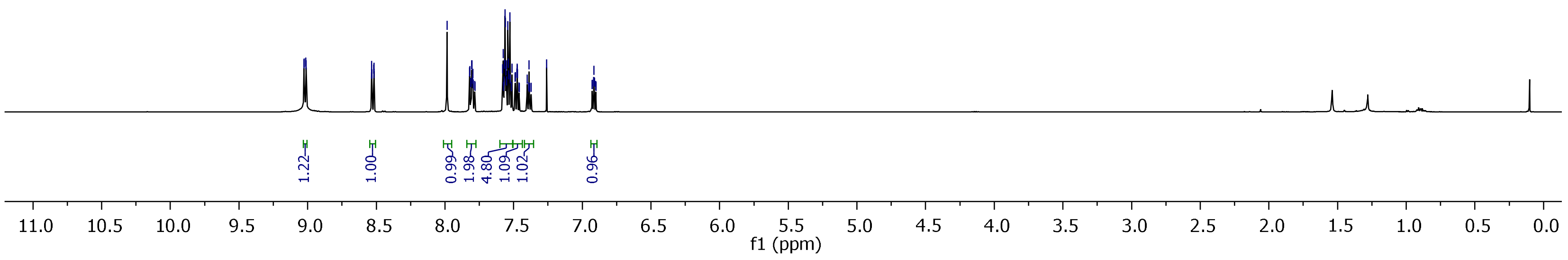

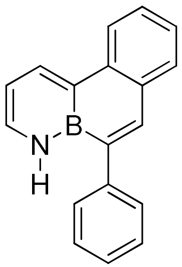

11

^{13}C-NMR (125 MHz, $CDCl_3$)

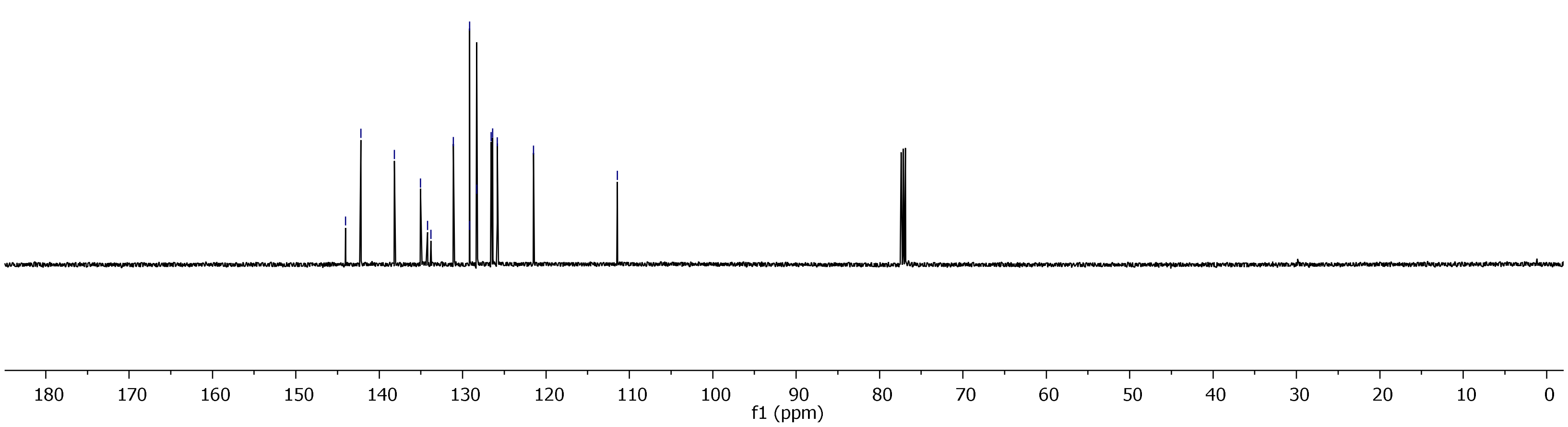

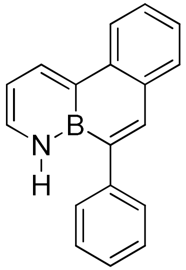

11

^{11}B-NMR (160 MHz, $CDCl_3$)

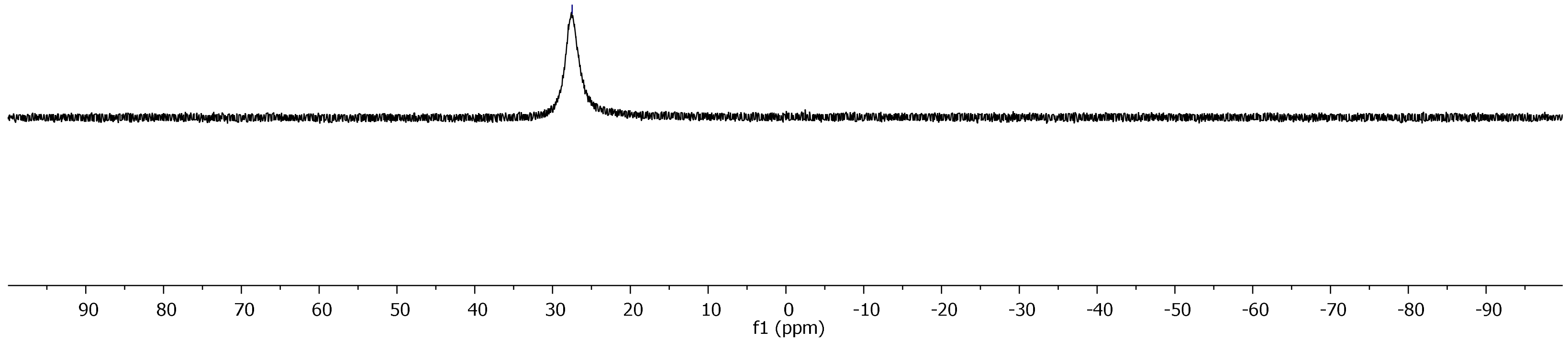

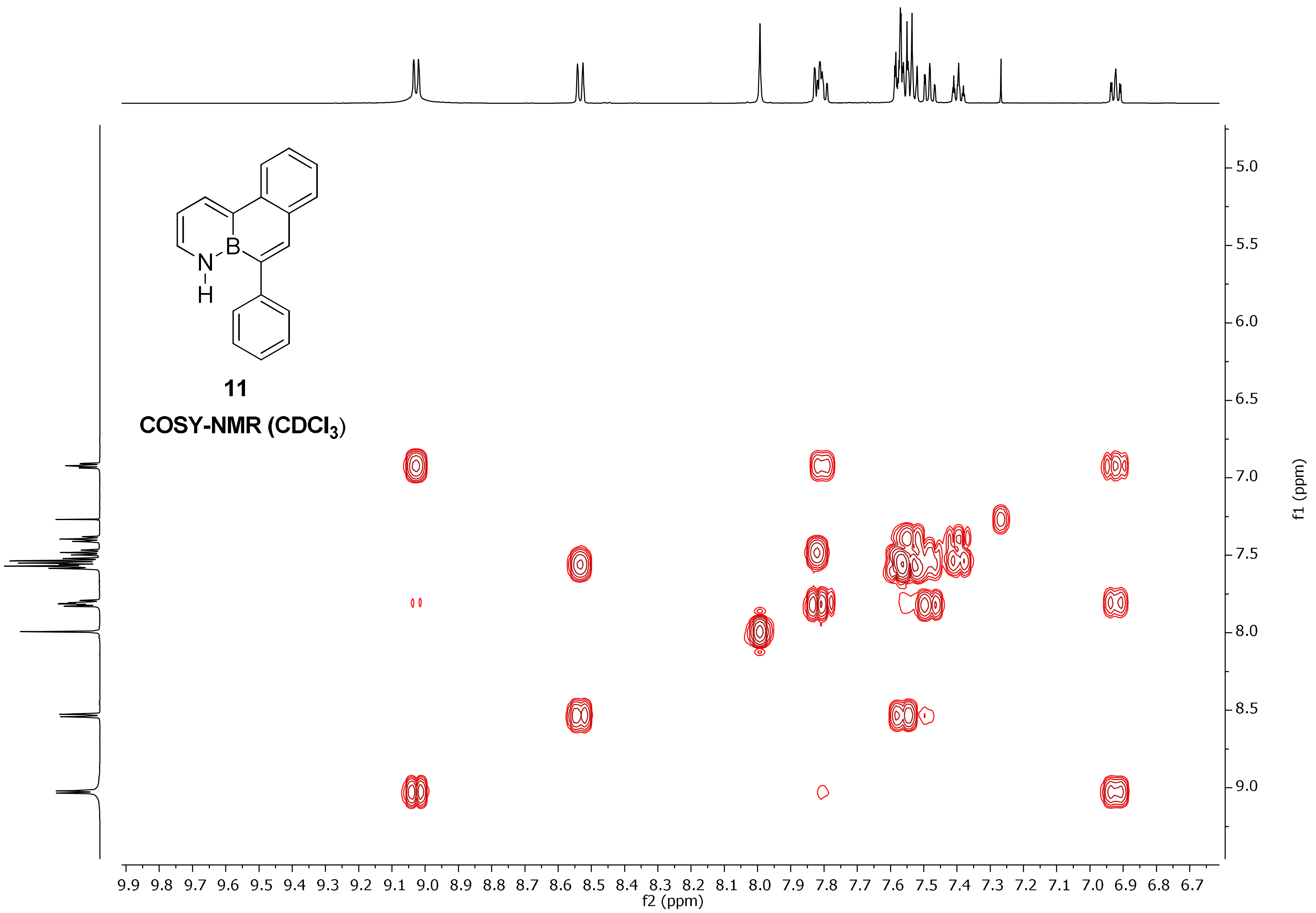
11
COSY-NMR (CDCl3)
f2 (ppm)
f1 (ppm)

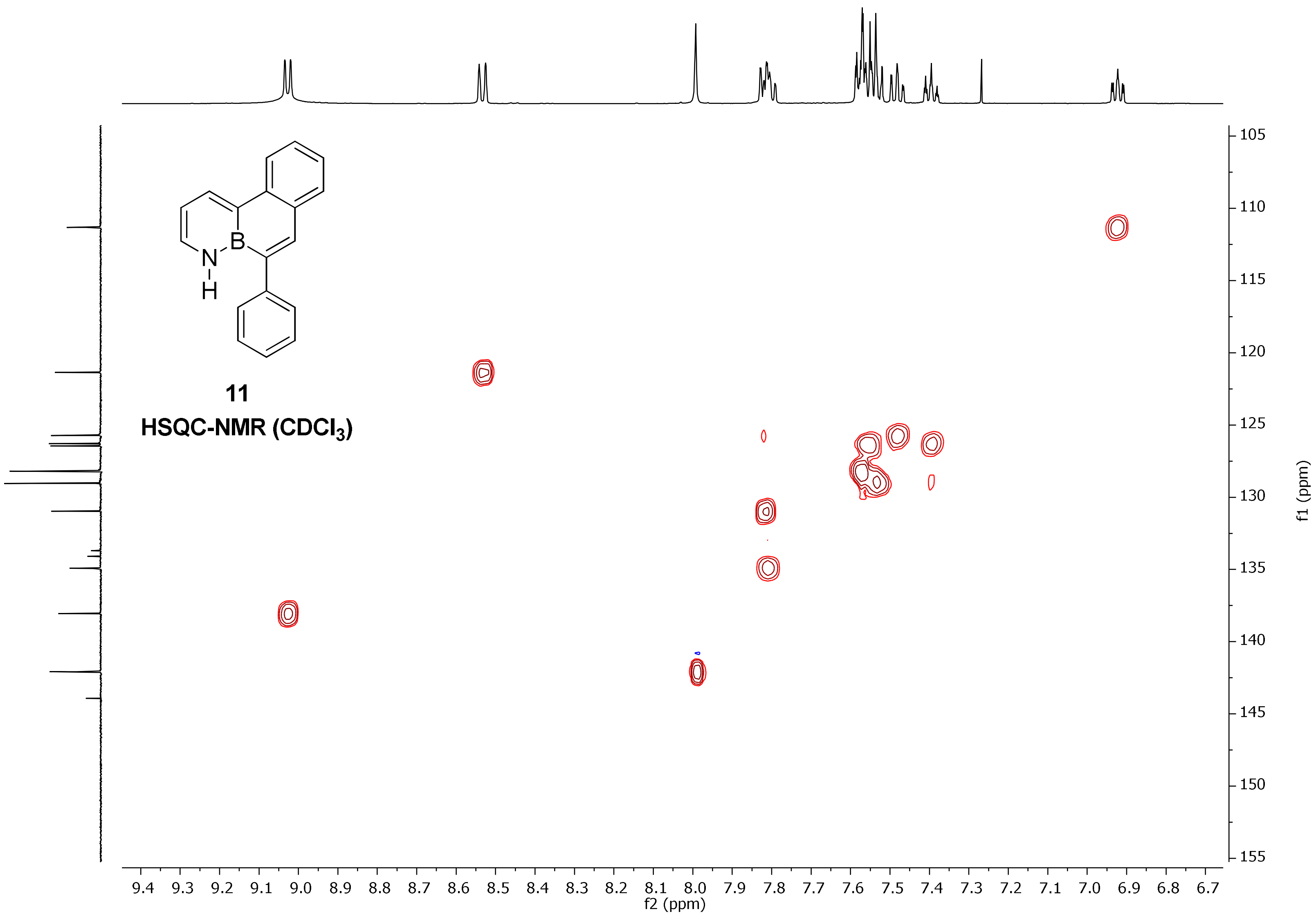
11
HSQC-NMR (CDCl3)
f2 (ppm)
f1 (ppm)
9.4 9.3 9.2 9.1 9.0 8.9 8.8 8.7 8.6 8.5 8.4 8.3 8.2 8.1 8.0 7.9 7.8 7.7 7.6 7.5 7.4 7.3 7.2 7.1 7.0 6.9 6.8 6.7
105 110 115 120 125 130 135 140 145 150 155
N
B
H

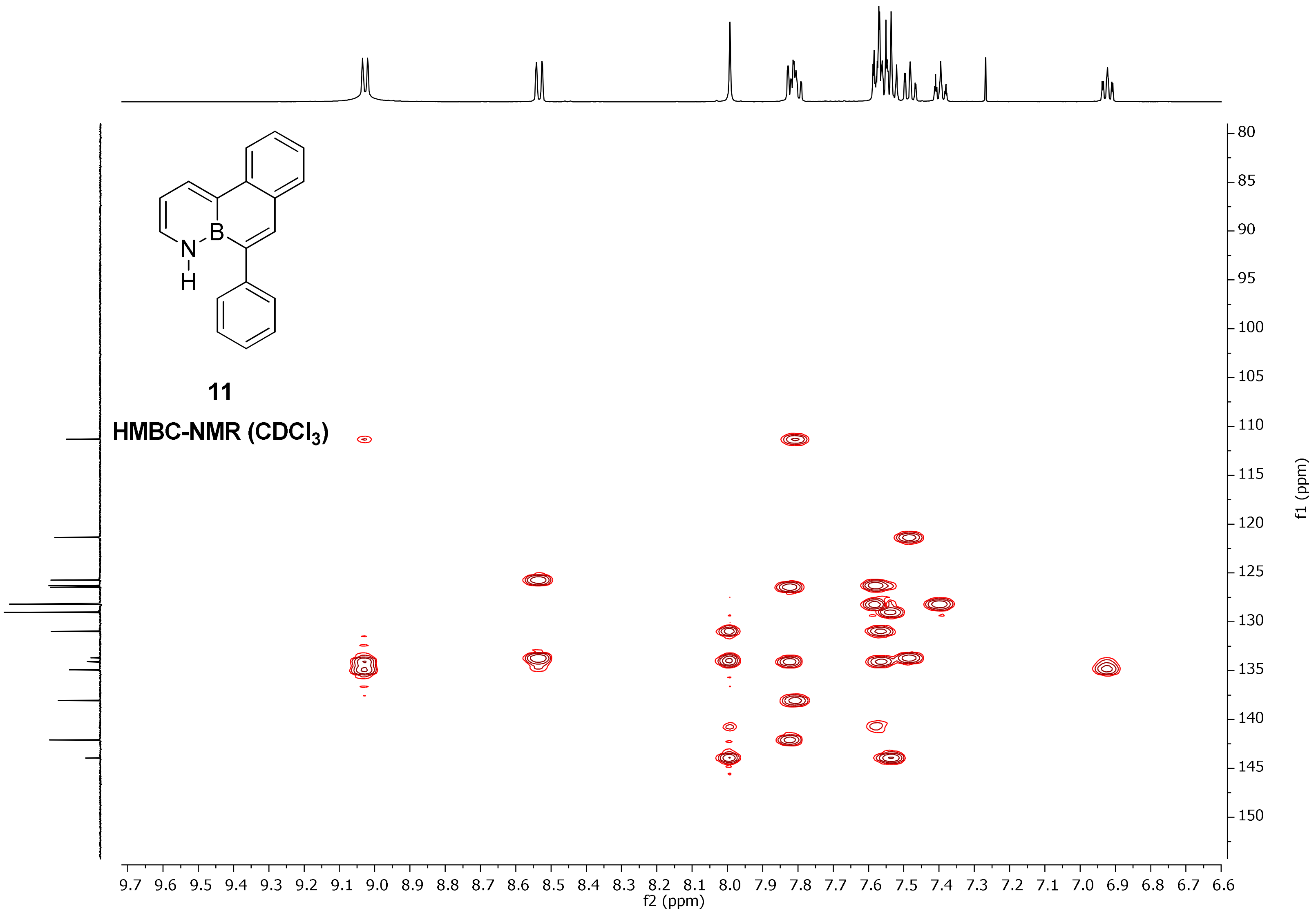
11
HMBC-NMR ($CDCl_3$)
f2 (ppm)
f1 (ppm)

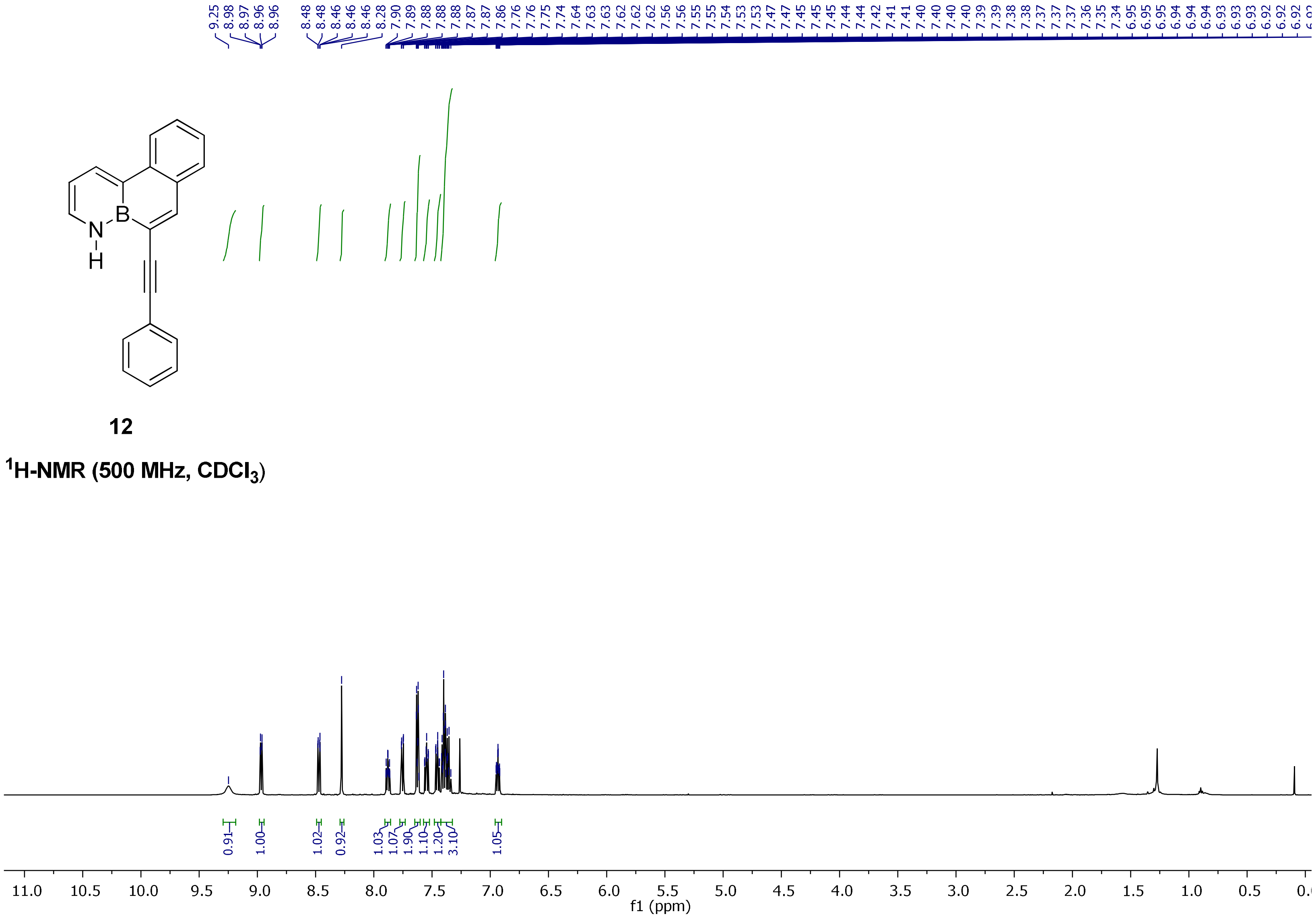

12

^{1}H-NMR (500 MHz, $CDCl_3$)

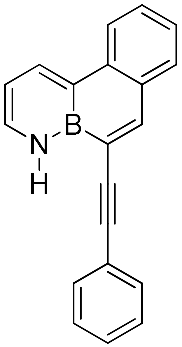

12

^{13}C-NMR (125MHz, CDCl$_3$)

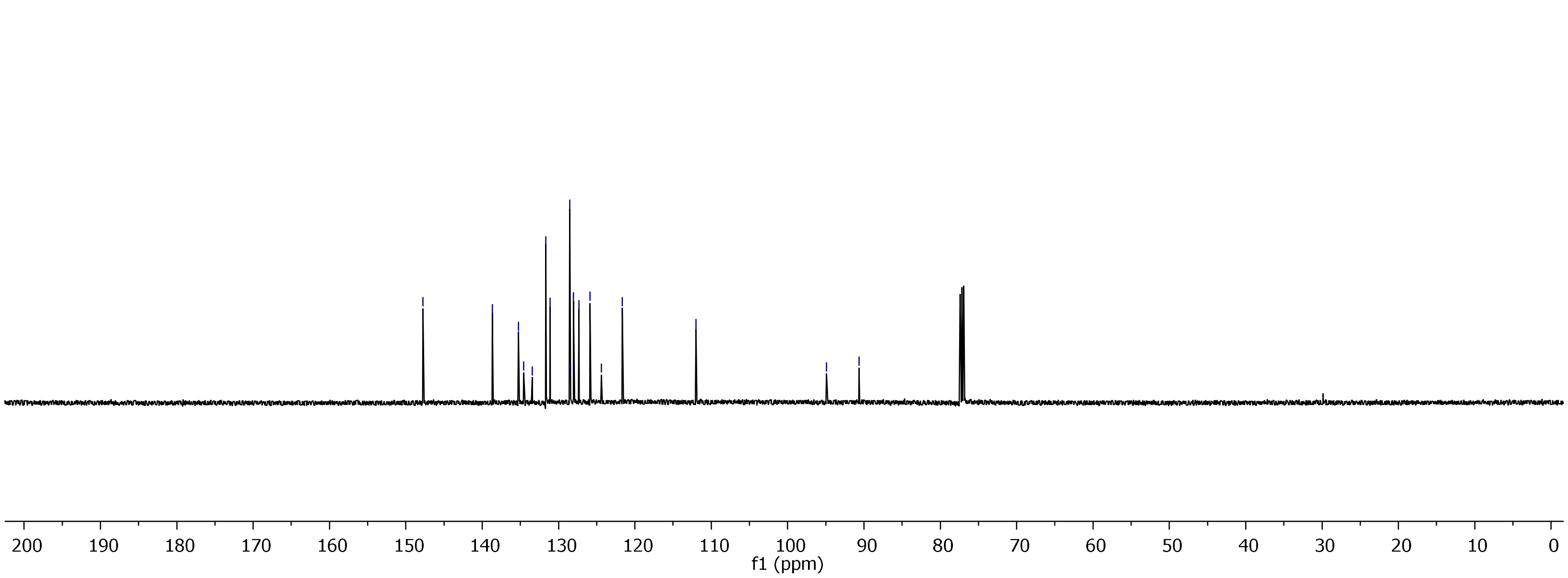

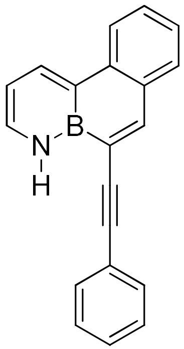

12

^{11}B-NMR (160 MHz, $CDCl_3$)

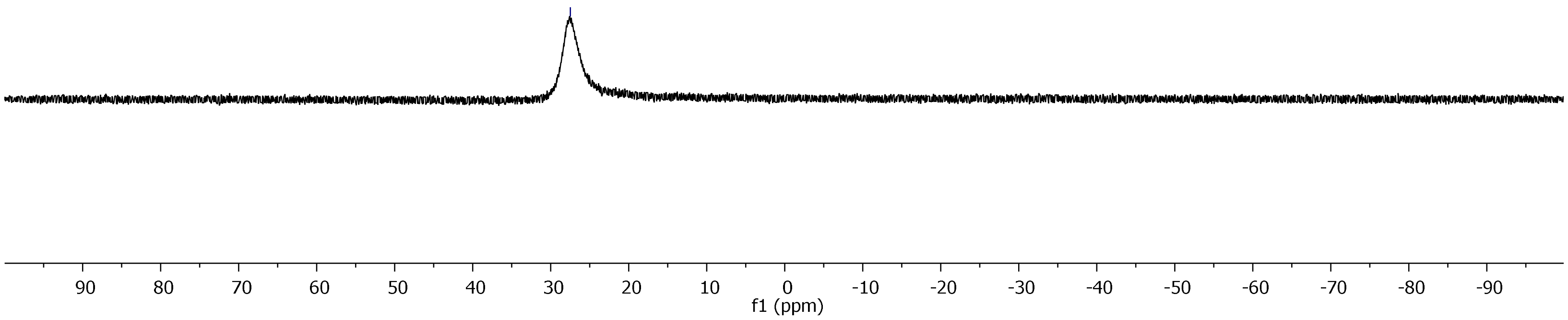

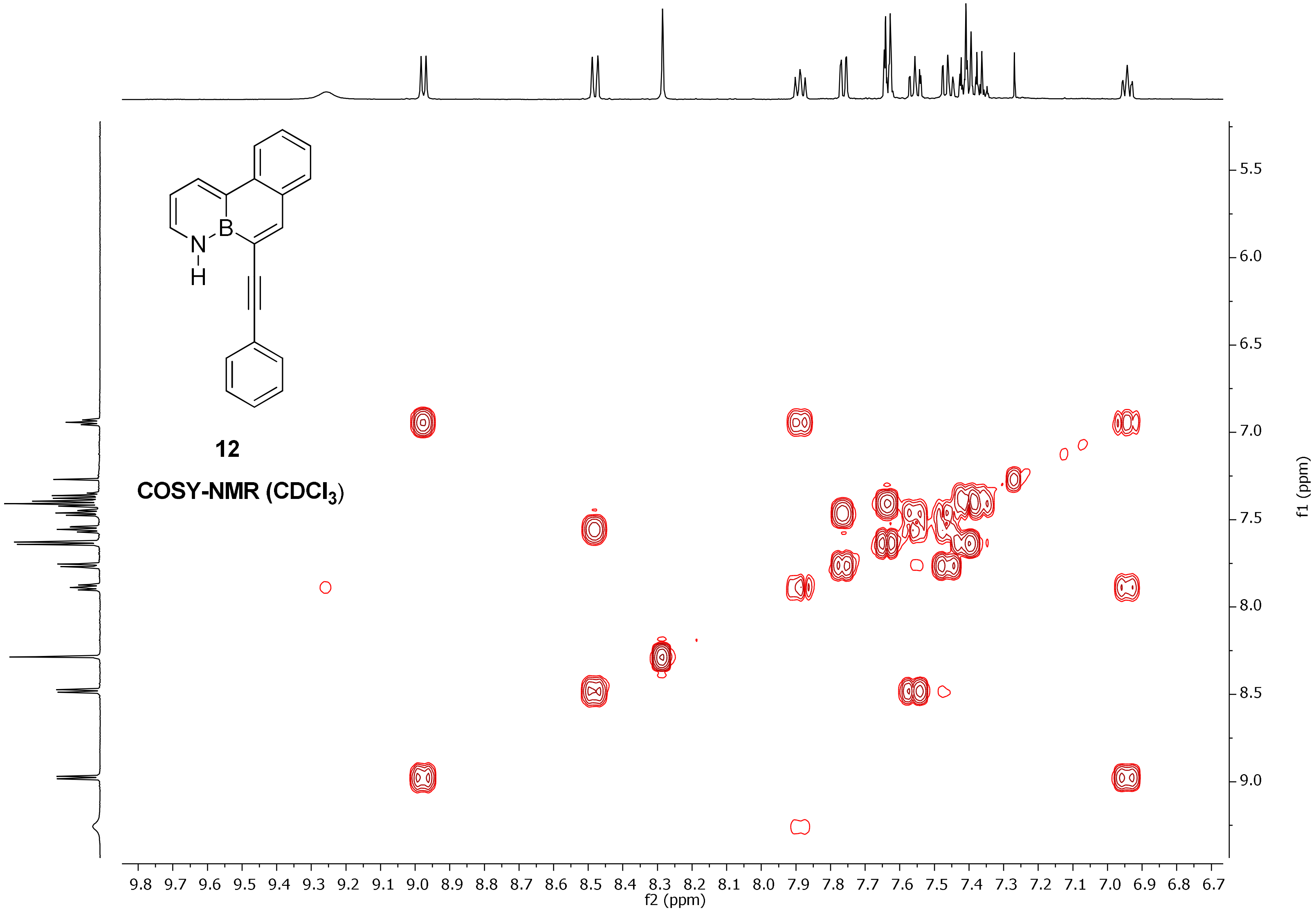
B
N
H
12
COSY-NMR (CDCl3)
f2 (ppm)
f1 (ppm)
9.8 9.7 9.6 9.5 9.4 9.3 9.2 9.1 9.0 8.9 8.8 8.7 8.6 8.5 8.4 8.3 8.2 8.1 8.0 7.9 7.8 7.7 7.6 7.5 7.4 7.3 7.2 7.1 7.0 6.9 6.8 6.7
5.5 6.0 6.5 7.0 7.5 8.0 8.5 9.0

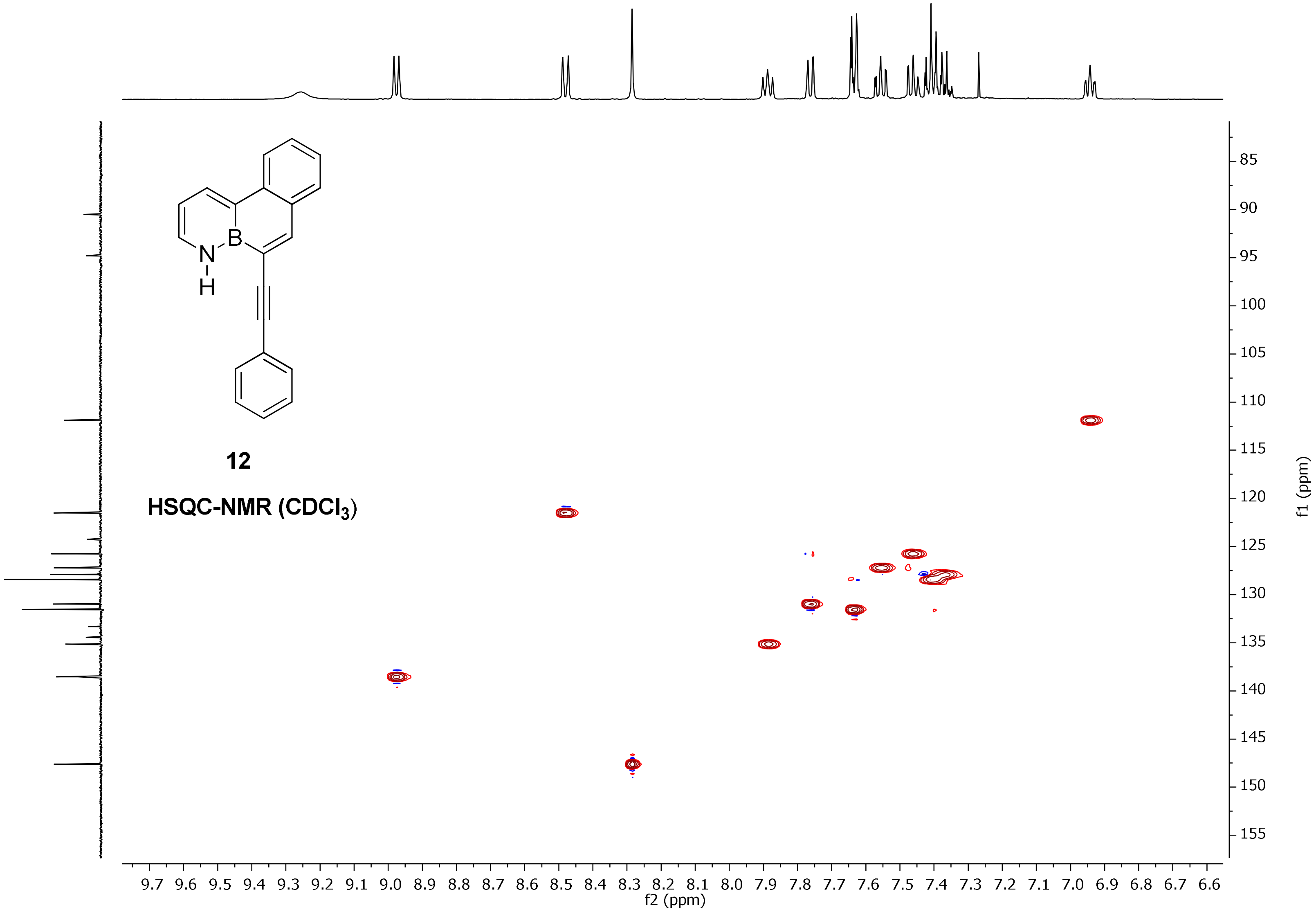
N
B
H
12
HSQC-NMR (CDCl$_3$)
f1 (ppm)
f2 (ppm)
85 90 95 100 105 110 115 120 125 130 135 140 145 150 155
9.7 9.6 9.5 9.4 9.3 9.2 9.1 9.0 8.9 8.8 8.7 8.6 8.5 8.4 8.3 8.2 8.1 8.0 7.9 7.8 7.7 7.6 7.5 7.4 7.3 7.2 7.1 7.0 6.9 6.8 6.7 6.6

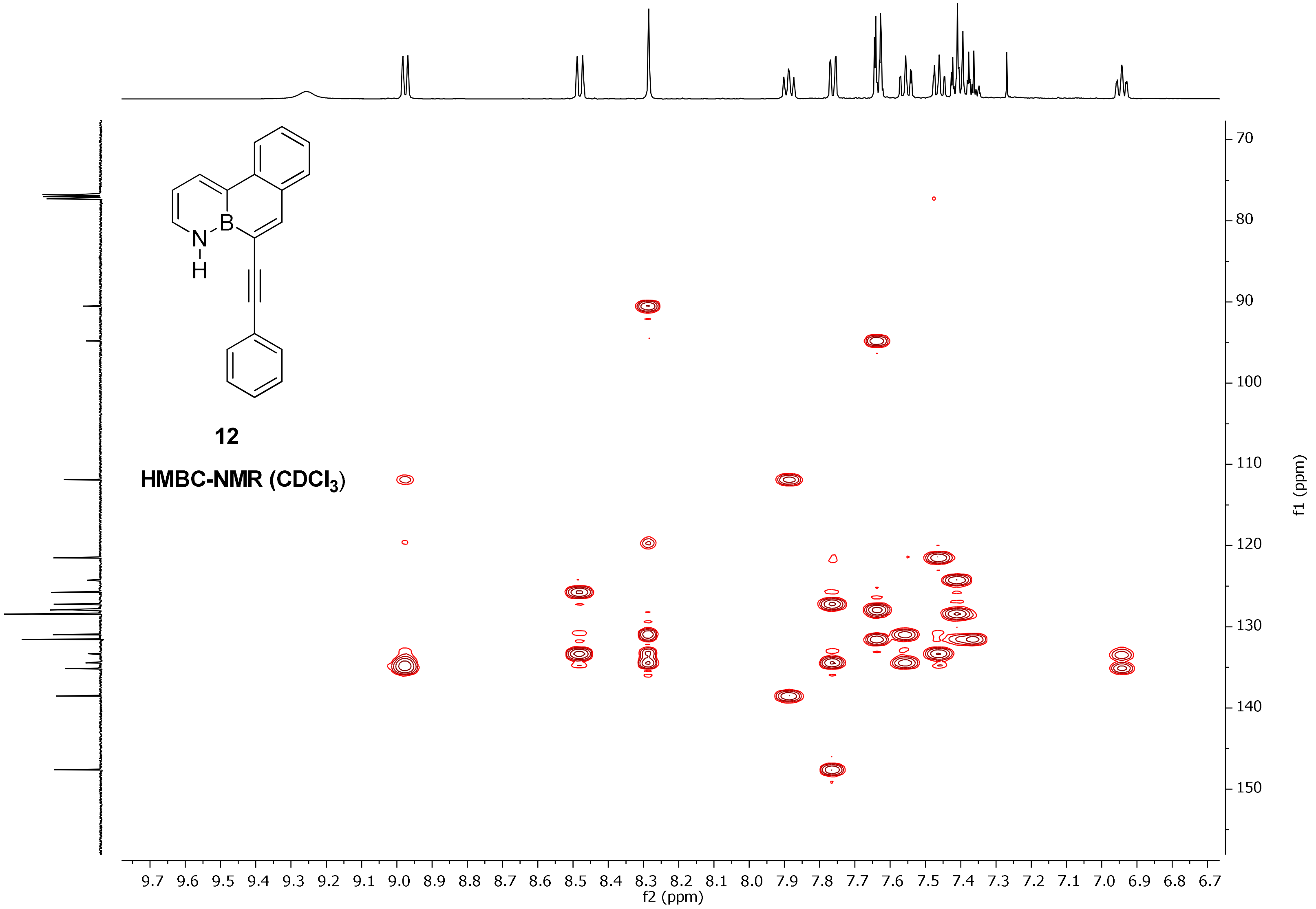
B
N
H
12
HMBC-NMR (CDCl3)
9.7 9.6 9.5 9.4 9.3 9.2 9.1 9.0 8.9 8.8 8.7 8.6 8.5 8.4 8.3 8.2 8.1 8.0 7.9 7.8 7.7 7.6 7.5 7.4 7.3 7.2 7.1 7.0 6.9 6.8 6.7
f2 (ppm)
70
80
90
100
110
120
130
140
150
f1 (ppm)

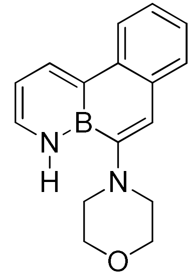

13

^{1}H-NMR (500 MHz, $CDCl_3$)

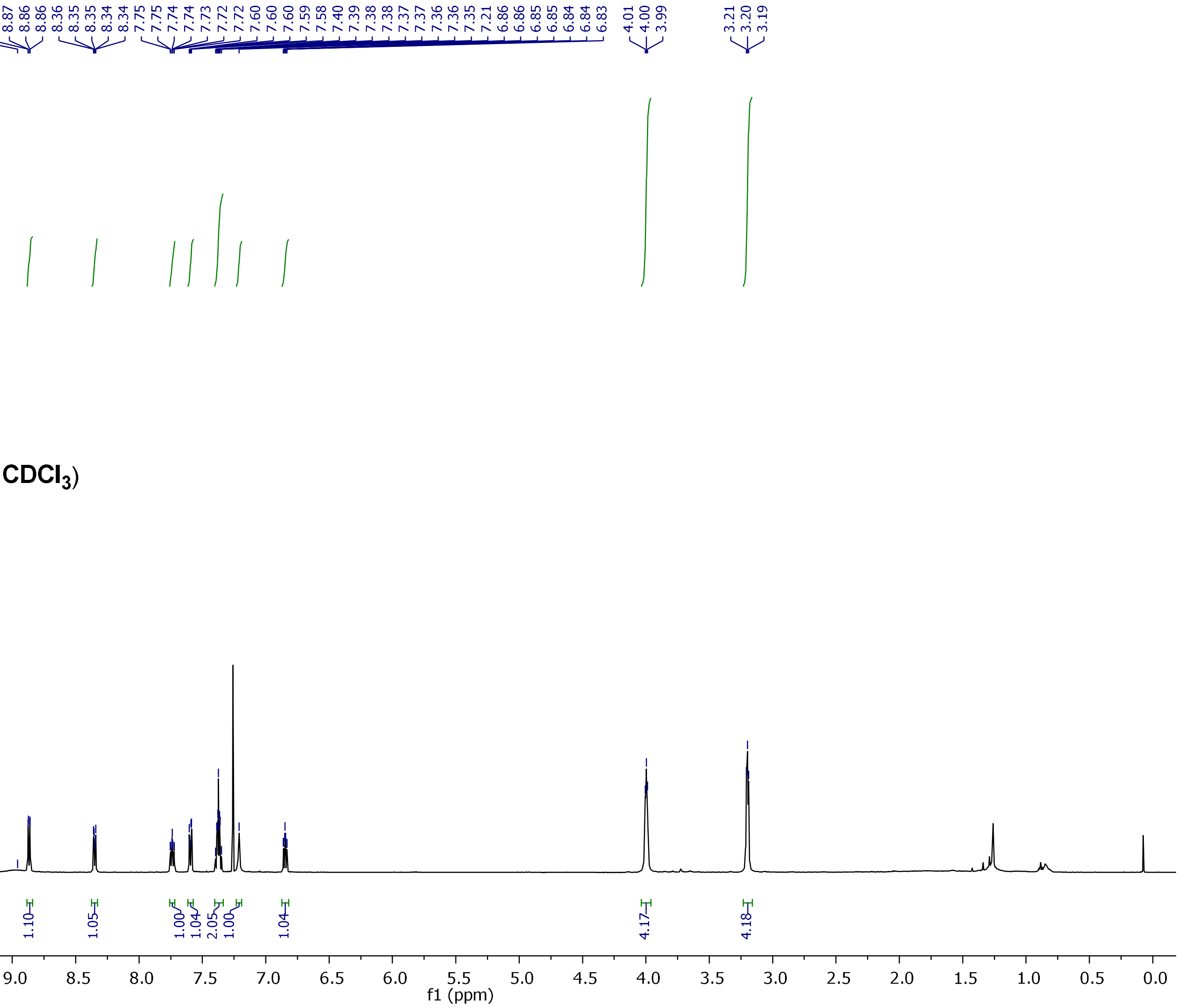

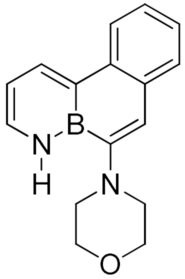

13

^{13}C-NMR (125 MHz, CDCl$_3$)

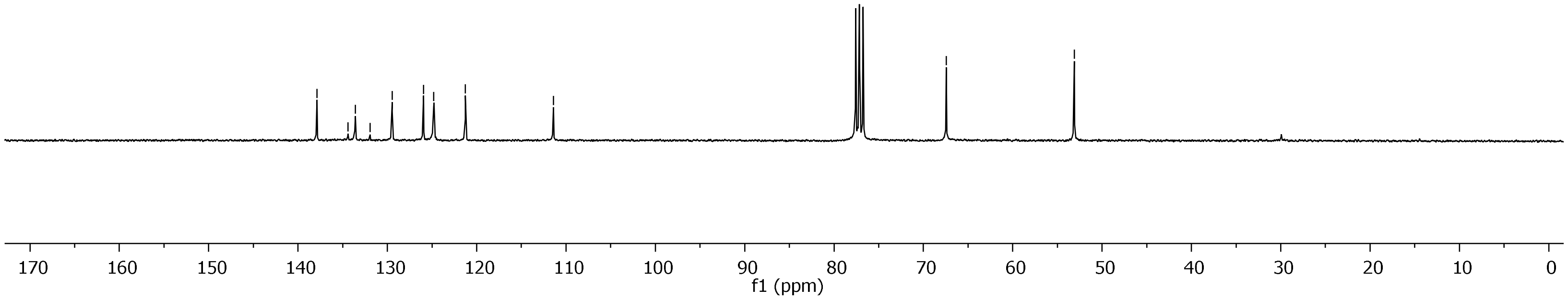

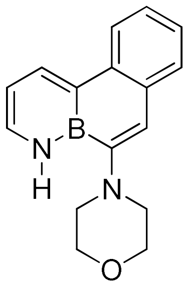

13

^{11}B-NMR (160 MHz, $CDCl_3$)

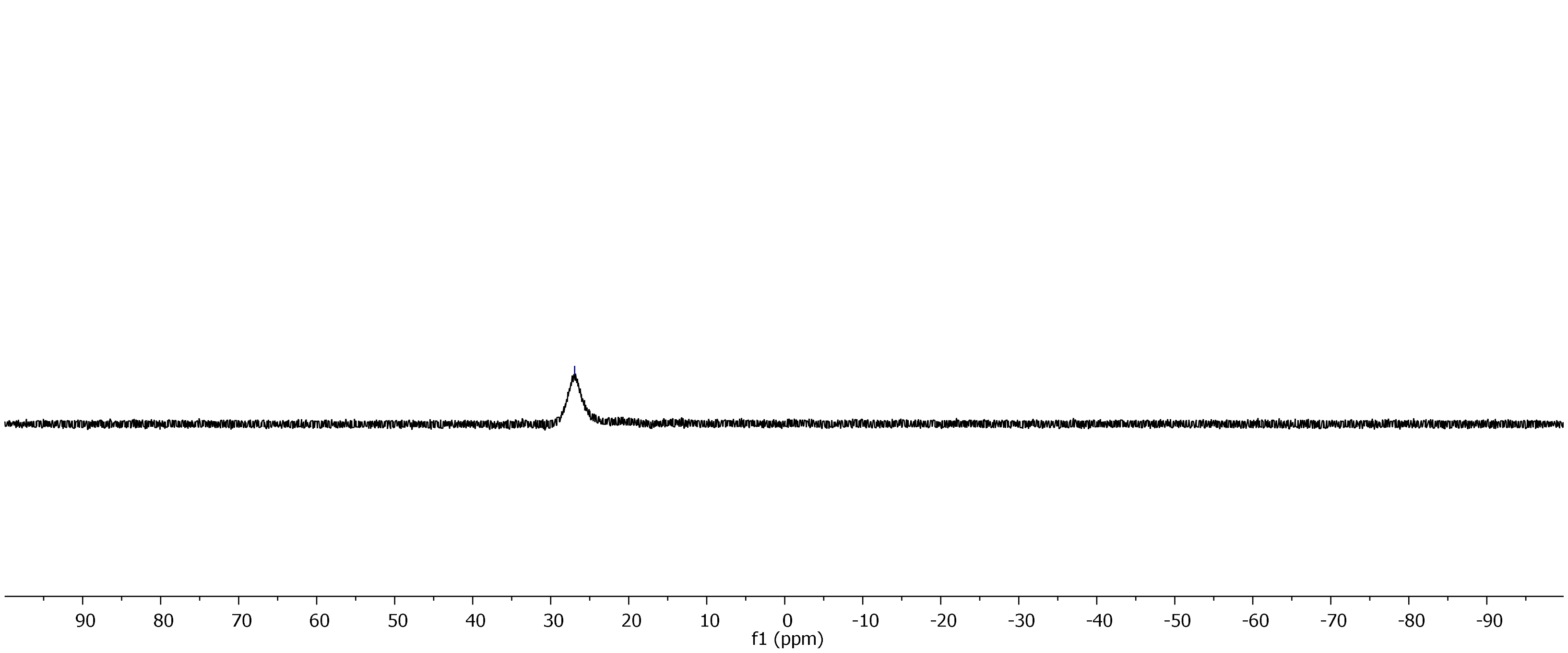

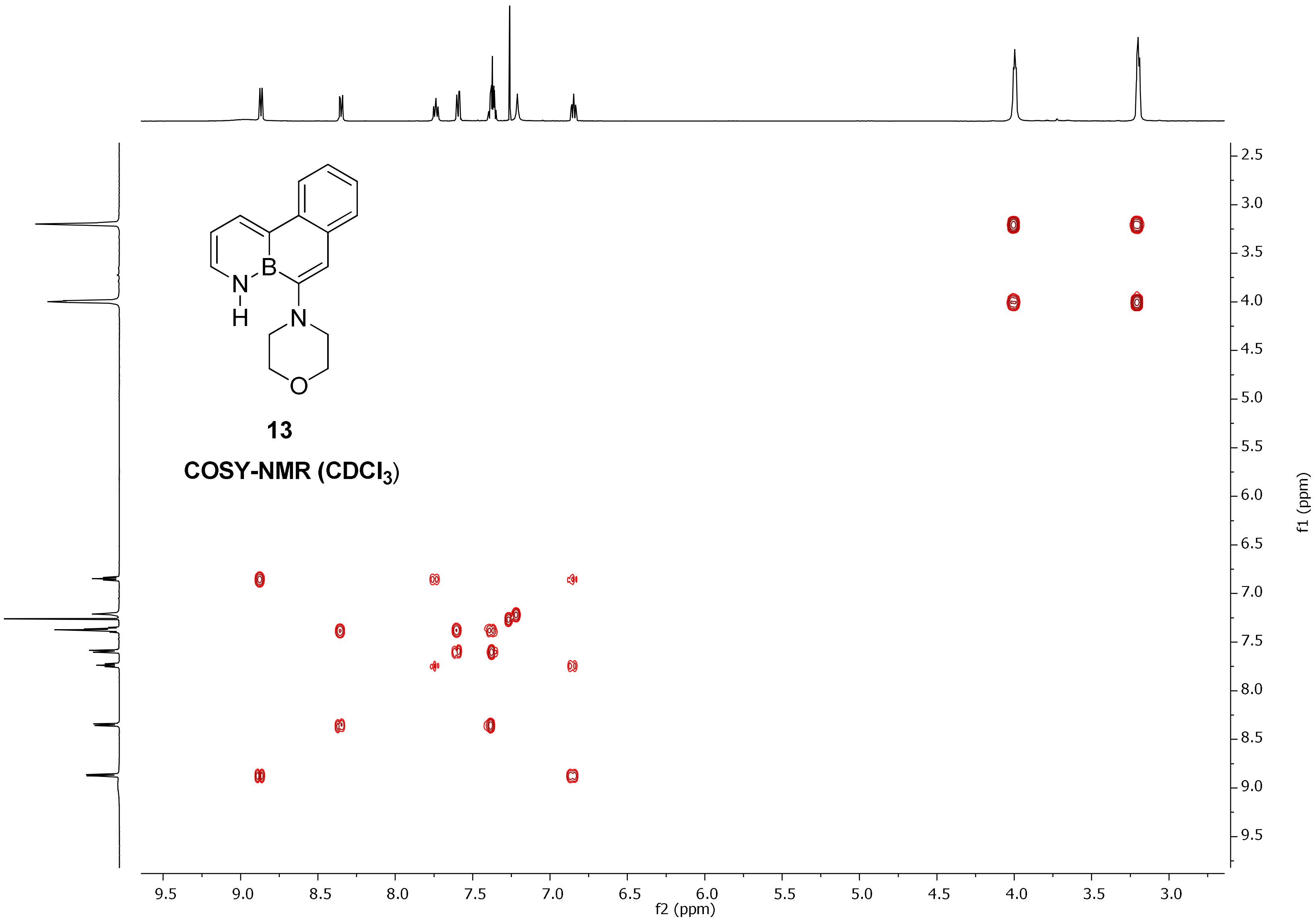

B
N
H
N
O
13
COSY-NMR ($CDCl_3$)
f1 (ppm)
f2 (ppm)
2.5
3.0
3.5
4.0
4.5
5.0
5.5
6.0
6.5
7.0
7.5
8.0
8.5
9.0
9.5

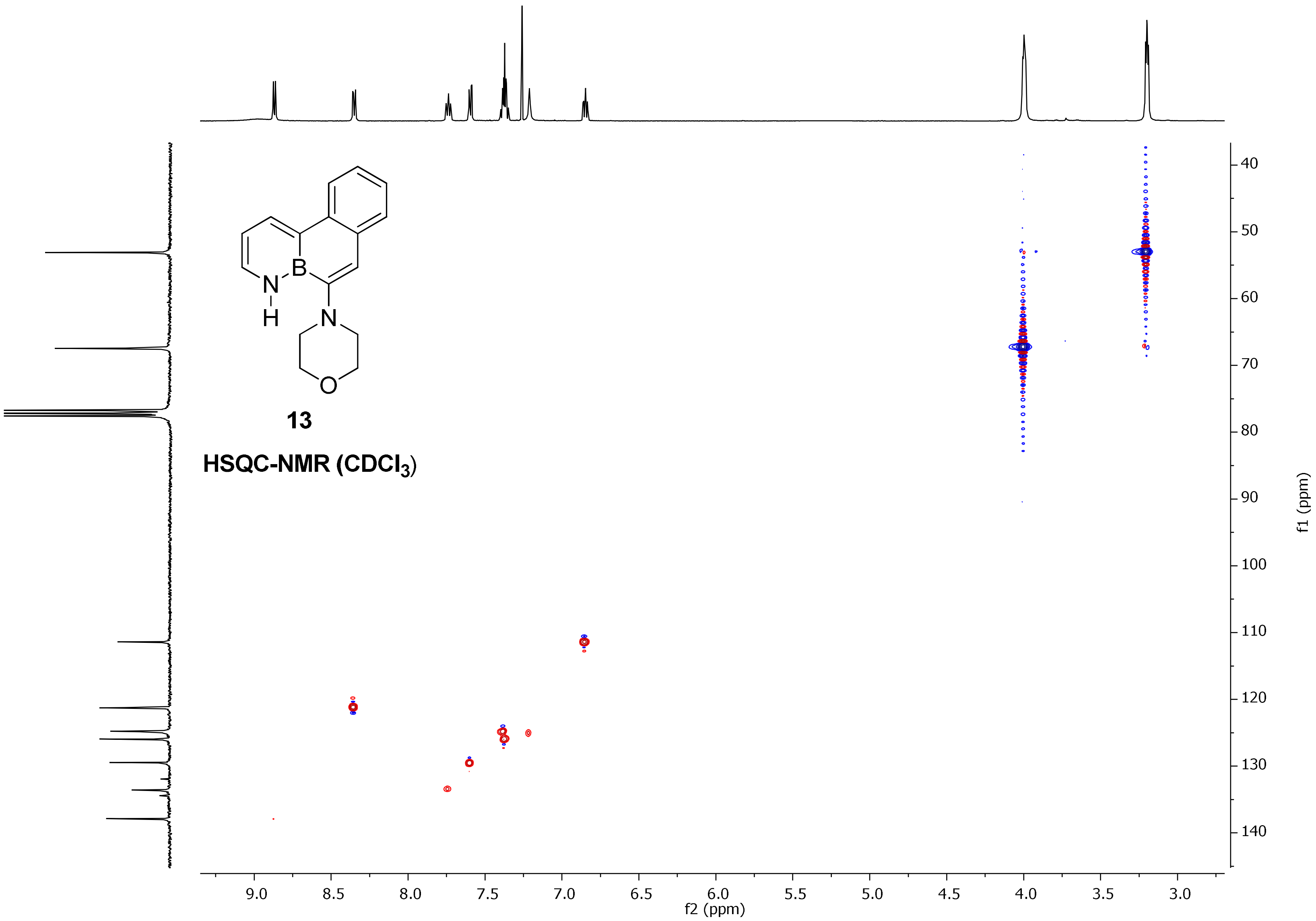
13
HSQC-NMR ($CDCl_3$)
f2 (ppm)
f1 (ppm)
9.0 8.5 8.0 7.5 7.0 6.5 6.0 5.5 5.0 4.5 4.0 3.5 3.0
40 50 60 70 80 90 100 110 120 130 140

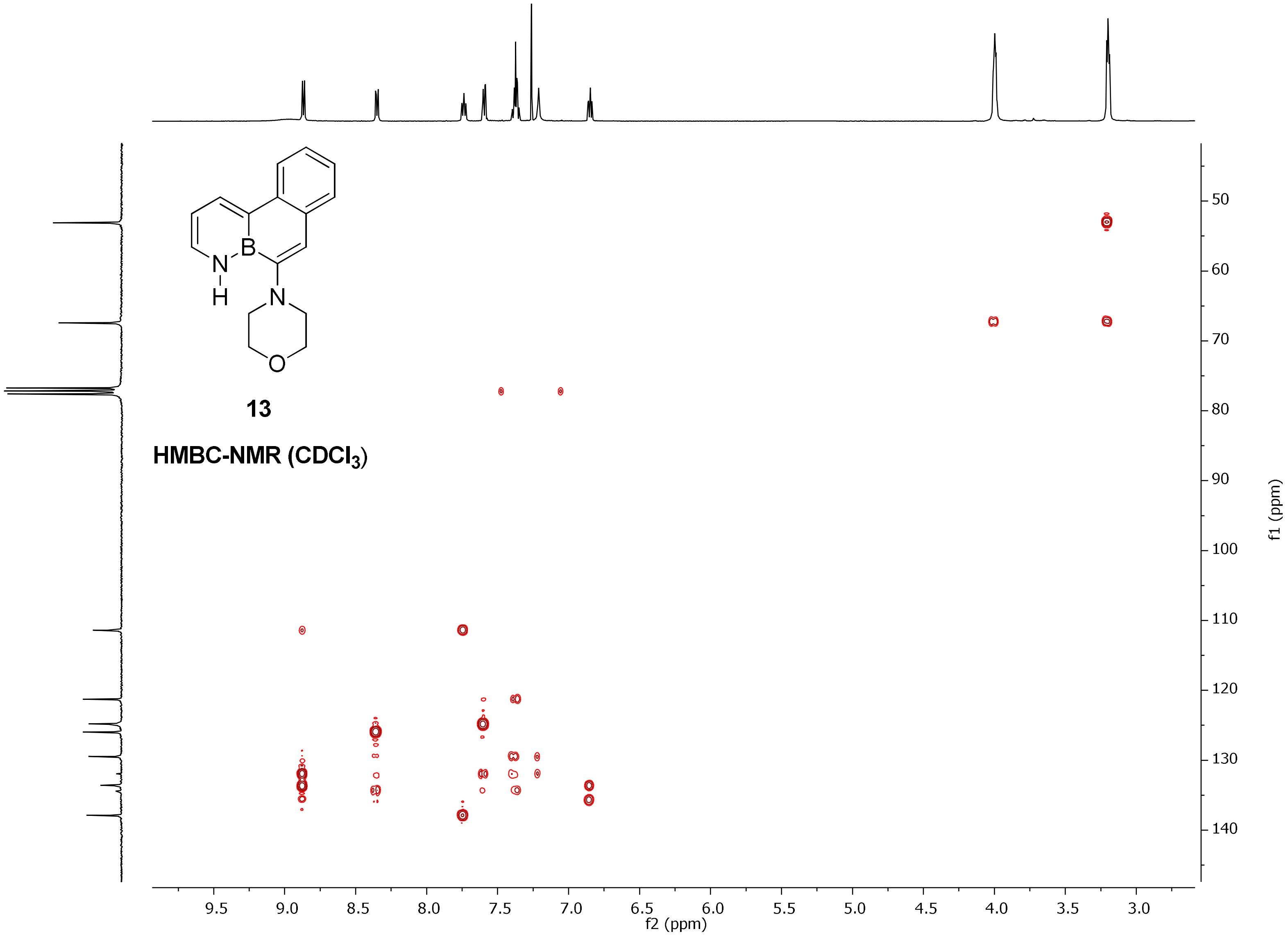

B
N
H
N
O
13
HMBC-NMR (CDCl3)
50
60
70
80
90
100
110
120
130
140
f1 (ppm)
9.5
9.0
8.5
8.0
7.5
7.0
6.5
6.0
5.5
5.0
4.5
4.0
3.5
3.0
f2 (ppm)

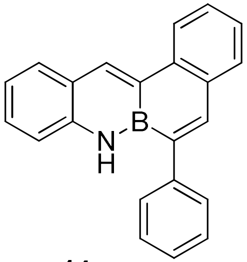

14

^{1}H-NMR (500 MHz, $CDCl_3$)

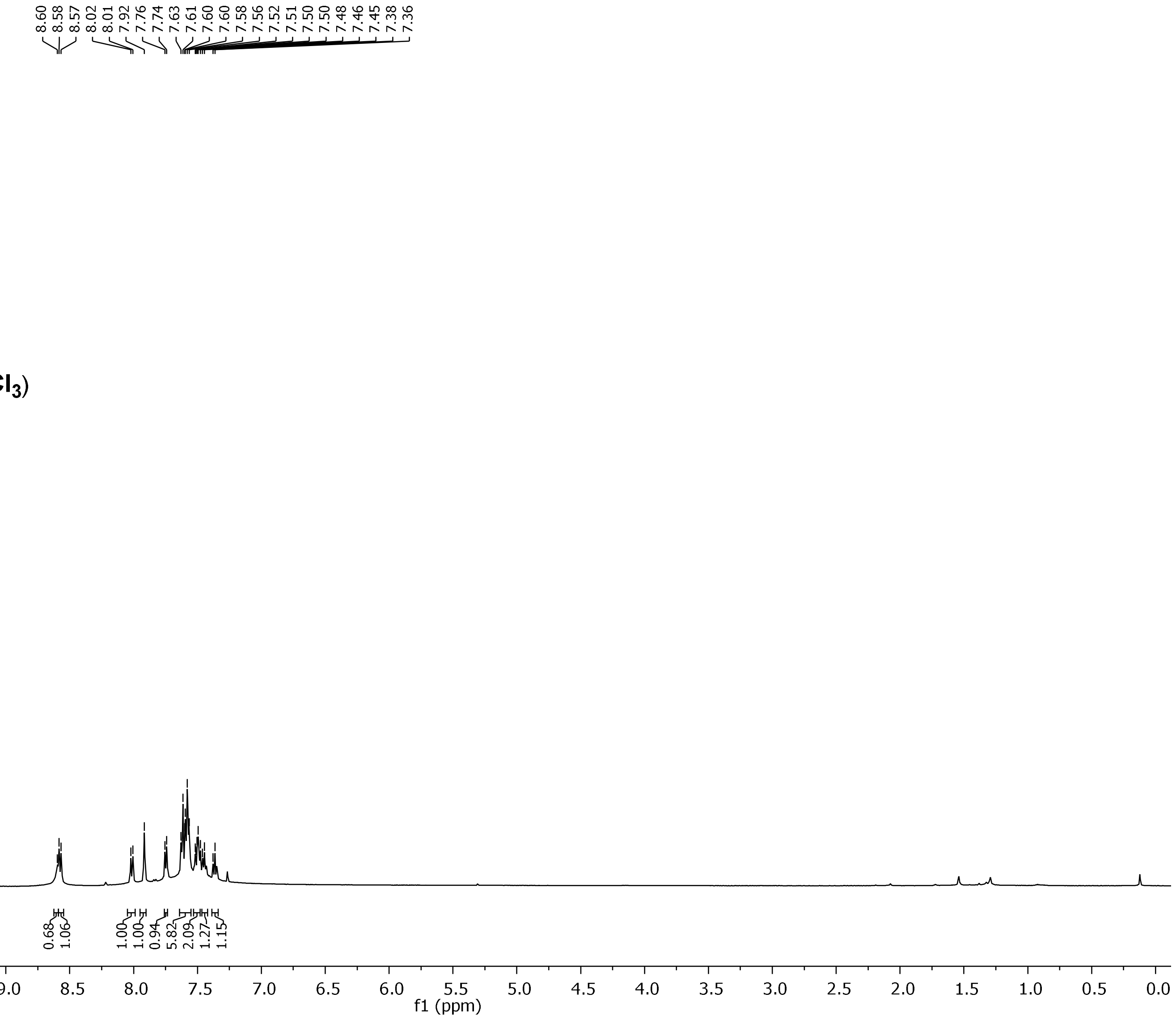

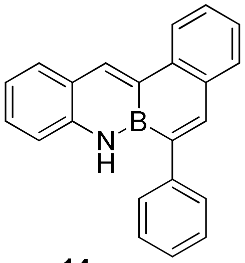

14

^{13}C-NMR (125 MHz, CDCl$_3$)

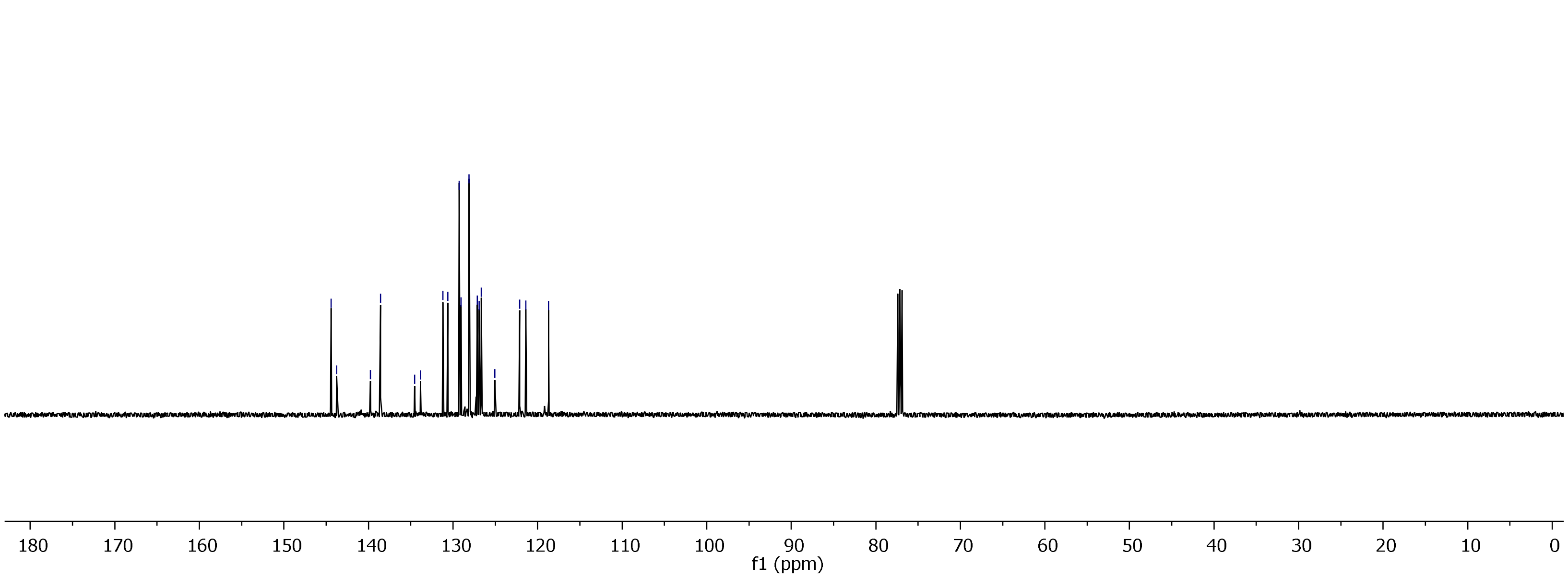

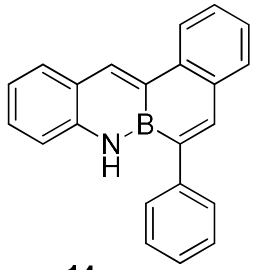

14

^{11}B-NMR (160 MHz, $CDCl_3$)

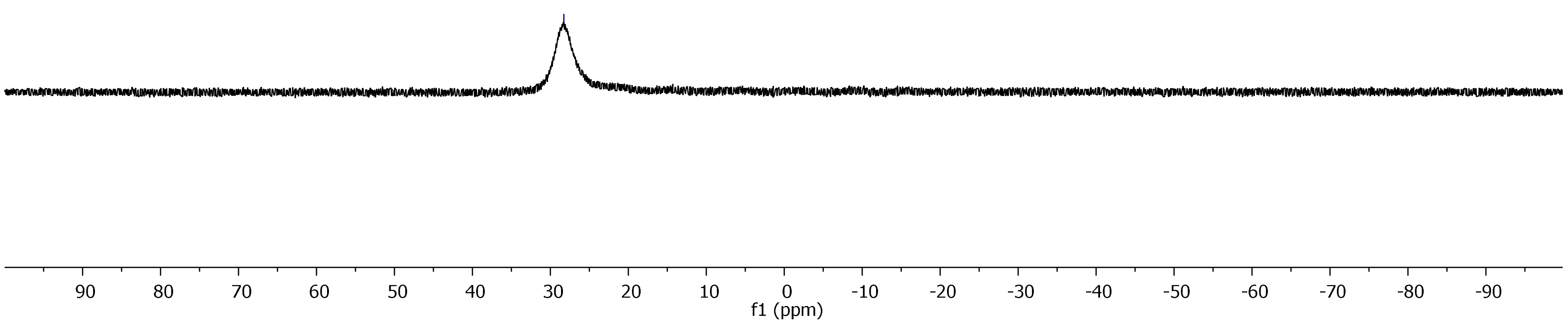

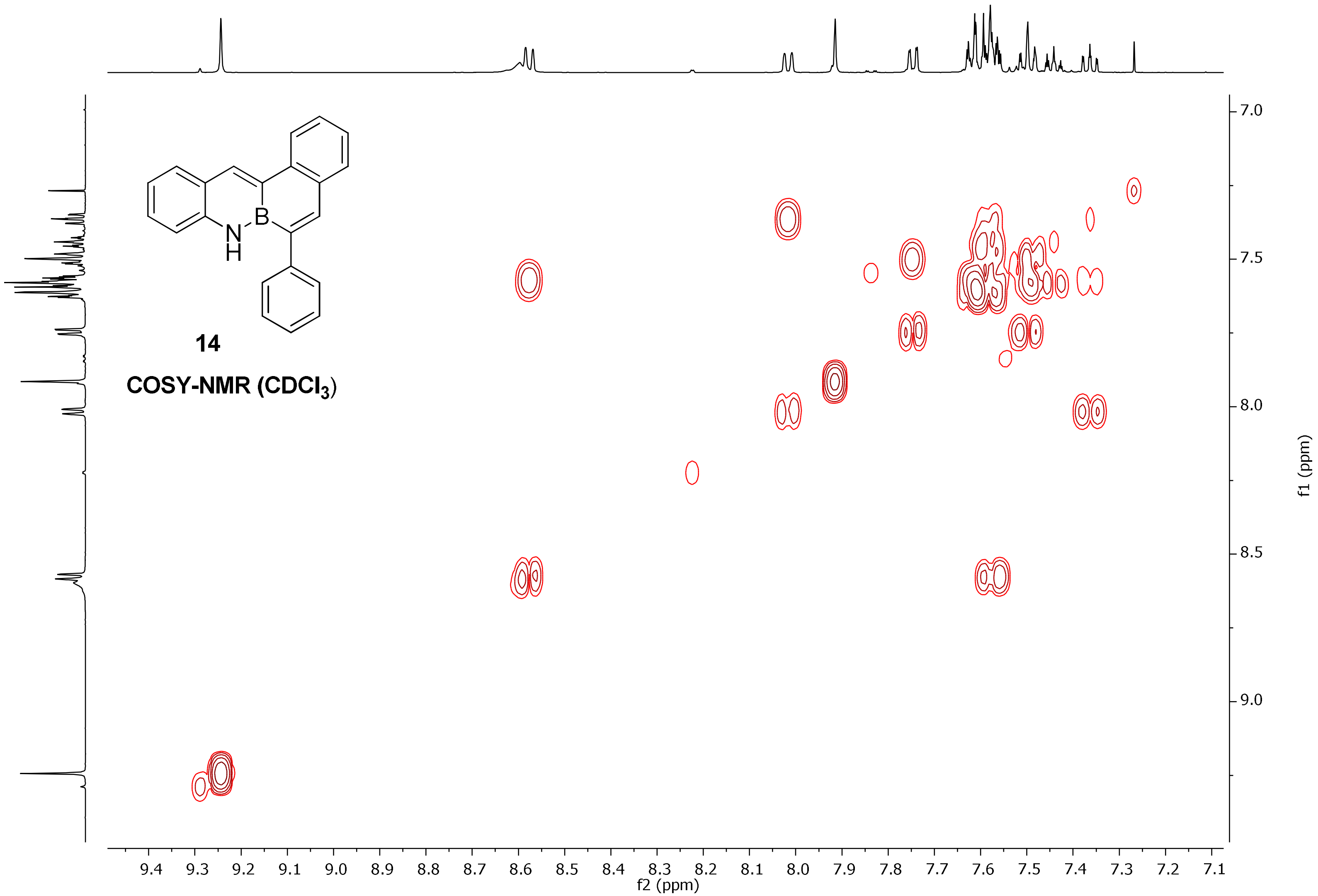

B
N
H
14
COSY-NMR ($CDCl_3$)
7.0
7.5
8.0
8.5
9.0
f1 (ppm)
9.4 9.3 9.2 9.1 9.0 8.9 8.8 8.7 8.6 8.5 8.4 8.3 8.2 8.1 8.0 7.9 7.8 7.7 7.6 7.5 7.4 7.3 7.2 7.1
f2 (ppm)

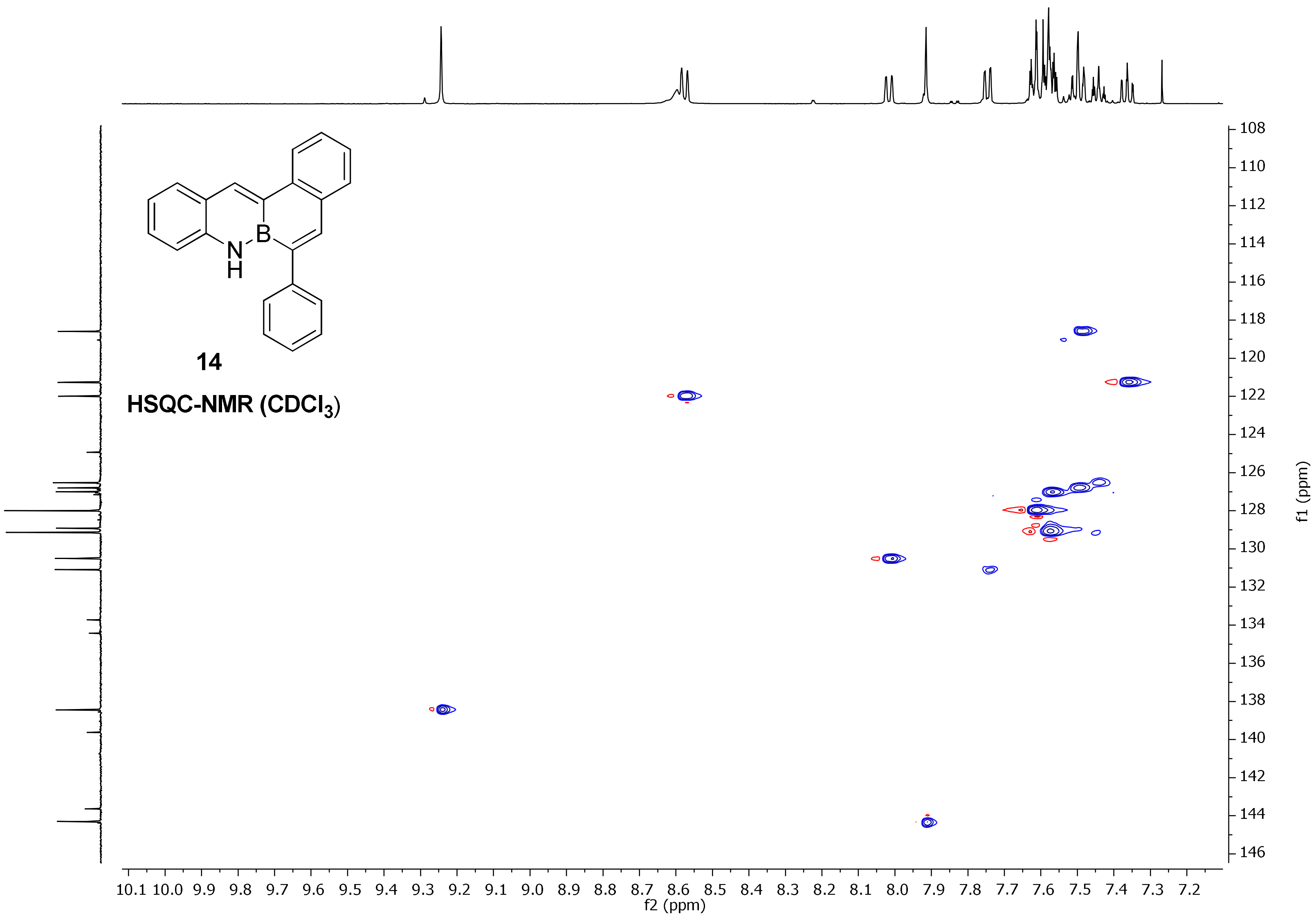
N
H
B
14
HSQC-NMR ($CDCl_3$)
f2 (ppm)
f1 (ppm)
10.1 10.0 9.9 9.8 9.7 9.6 9.5 9.4 9.3 9.2 9.1 9.0 8.9 8.8 8.7 8.6 8.5 8.4 8.3 8.2 8.1 8.0 7.9 7.8 7.7 7.6 7.5 7.4 7.3 7.2
108 110 112 114 116 118 120 122 124 126 128 130 132 134 136 138 140 142 144 146

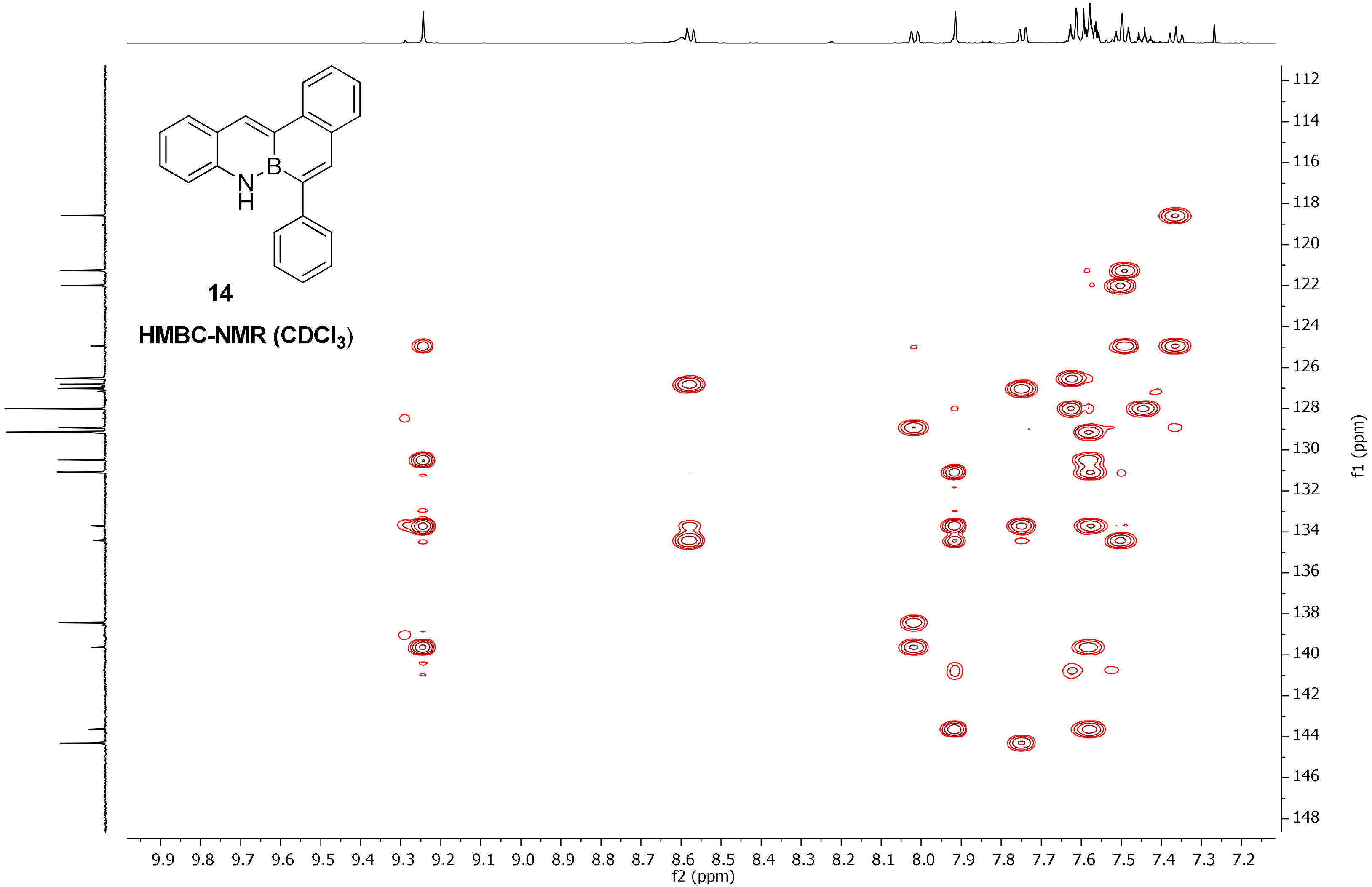
B
N
H
14
HMBC-NMR (CDCl3)
9.9 9.8 9.7 9.6 9.5 9.4 9.3 9.2 9.1 9.0 8.9 8.8 8.7 8.6 8.5 8.4 8.3 8.2 8.1 8.0 7.9 7.8 7.7 7.6 7.5 7.4 7.3 7.2
f2 (ppm)
112 114 116 118 120 122 124 126 128 130 132 134 136 138 140 142 144 146 148
f1 (ppm)

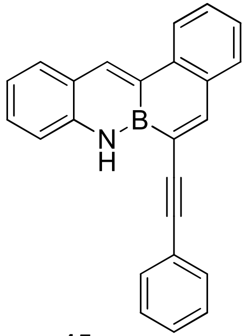

15

^{1}H-NMR (300 MHz, CDCl$_3$)

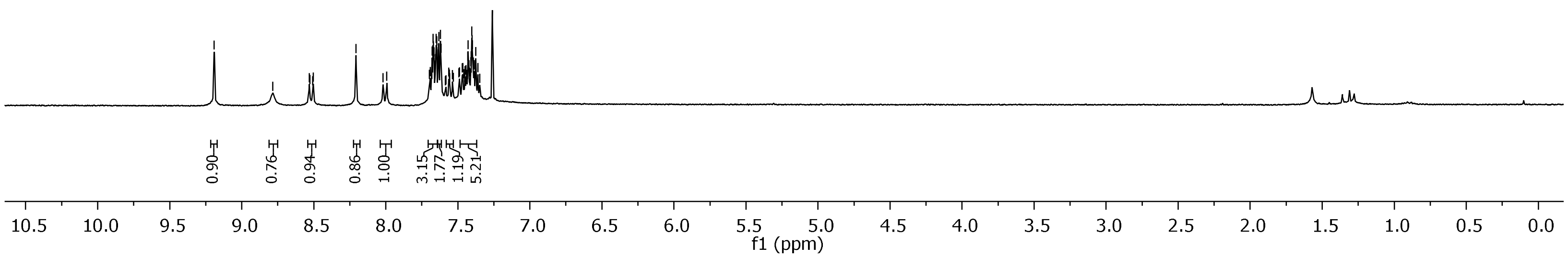

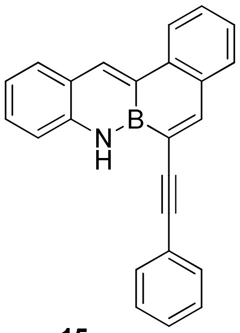

15

^{13}C-NMR (125 MHz, CDCl$_3$)

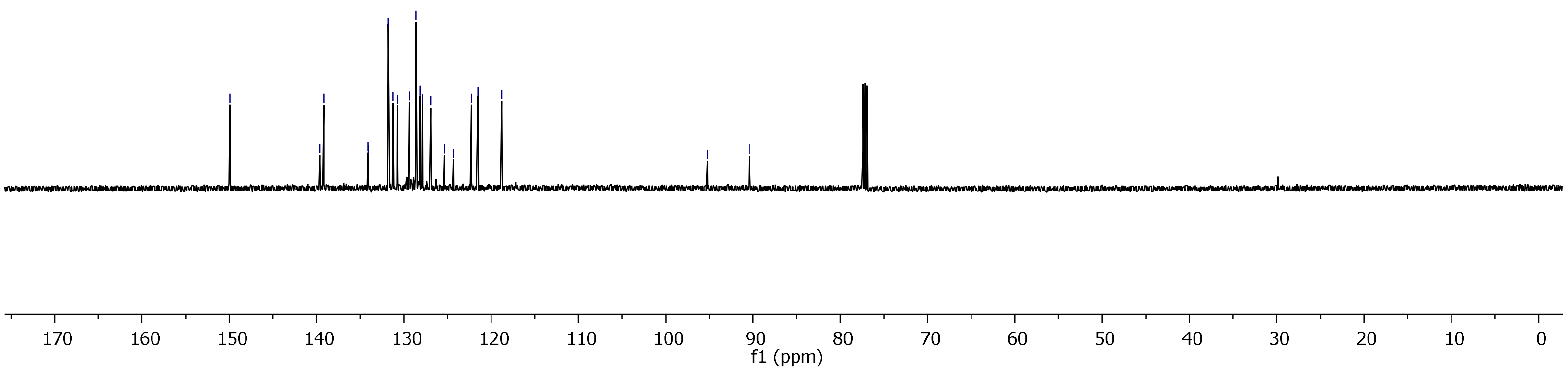

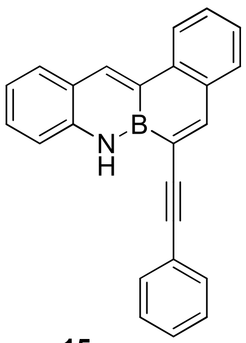

15

^{11}B-NMR (160 MHz, $CDCl_3$)

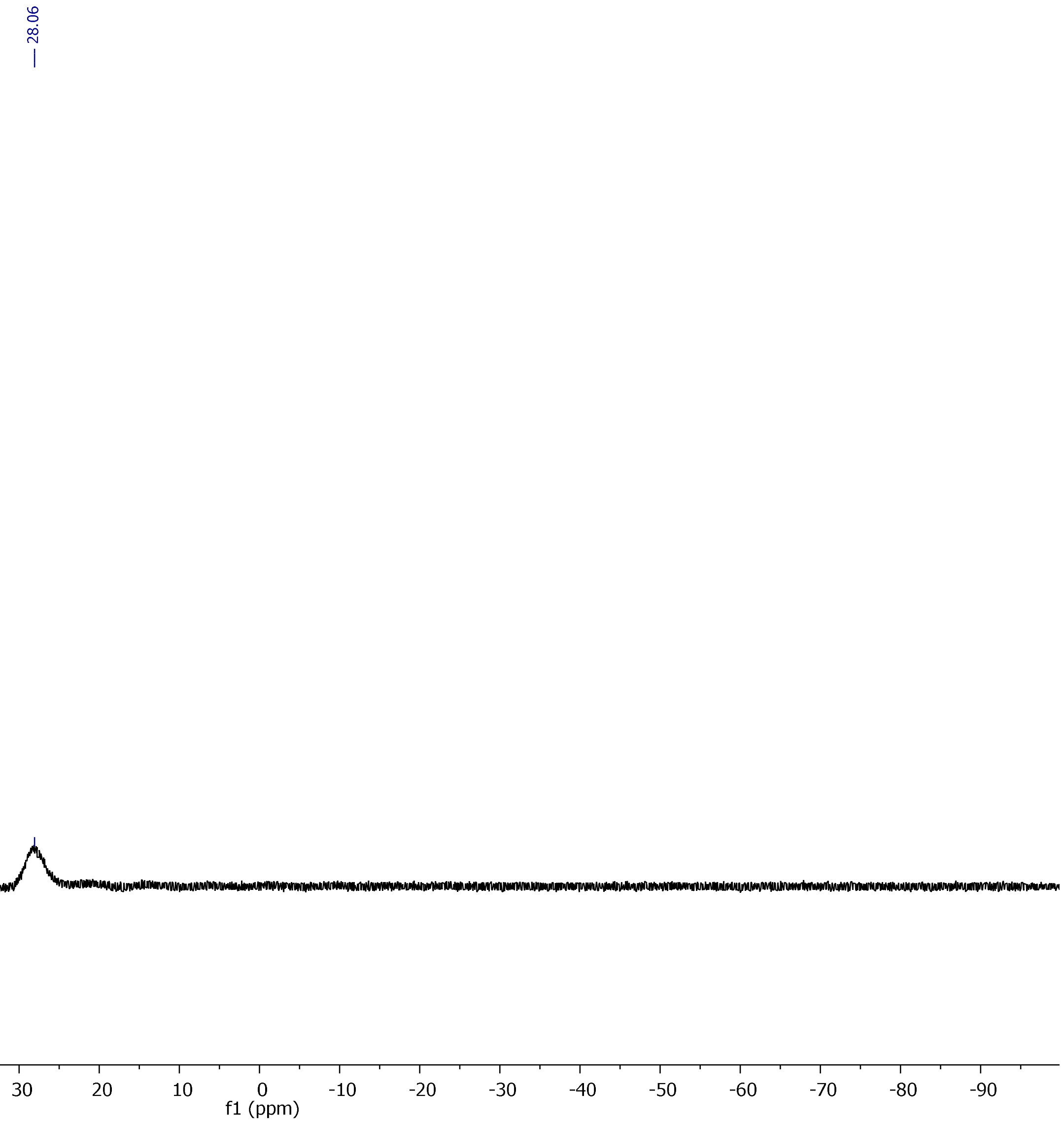

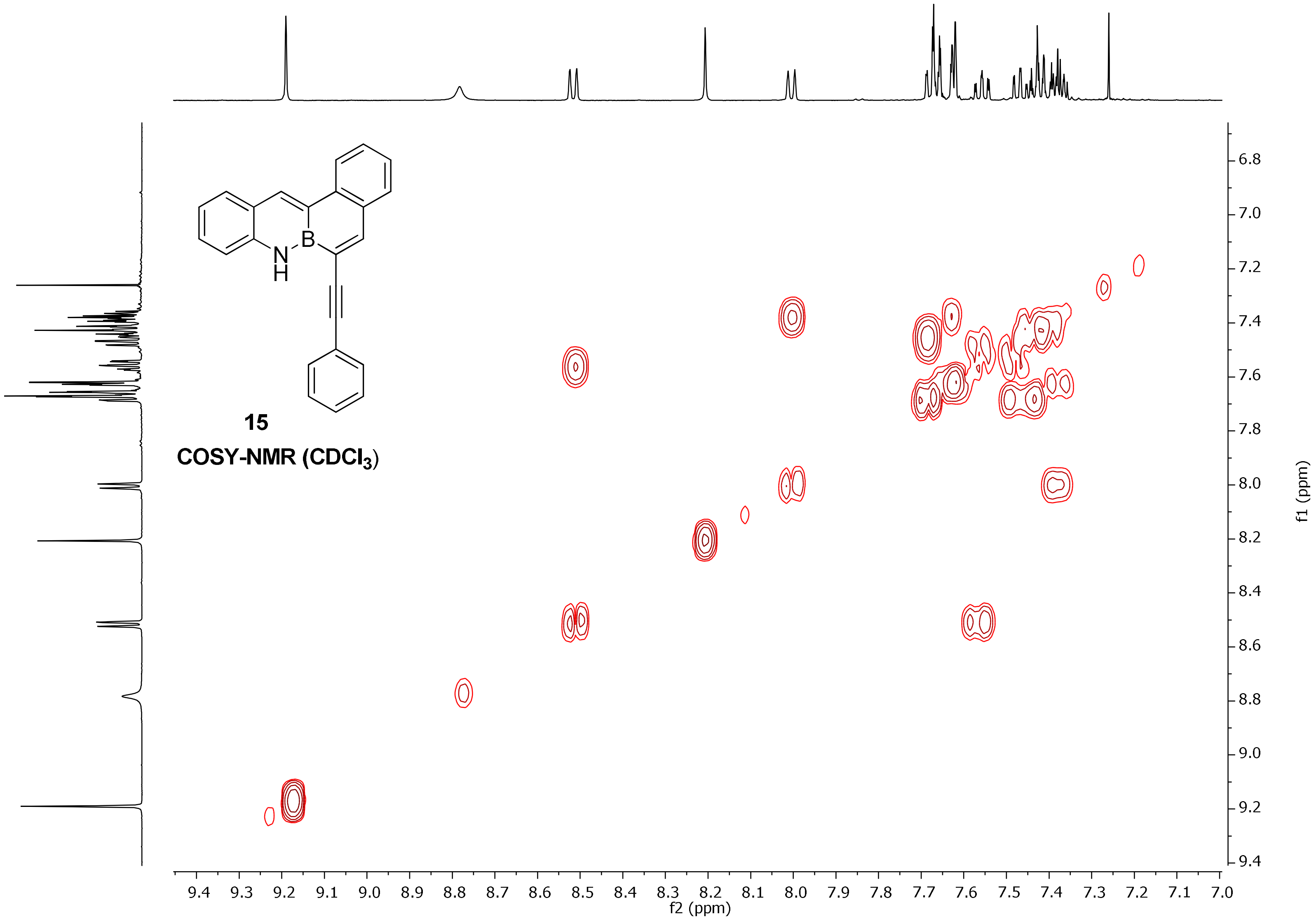

B
N
H
15
COSY-NMR ($CDCl_3$)
f2 (ppm)
f1 (ppm)

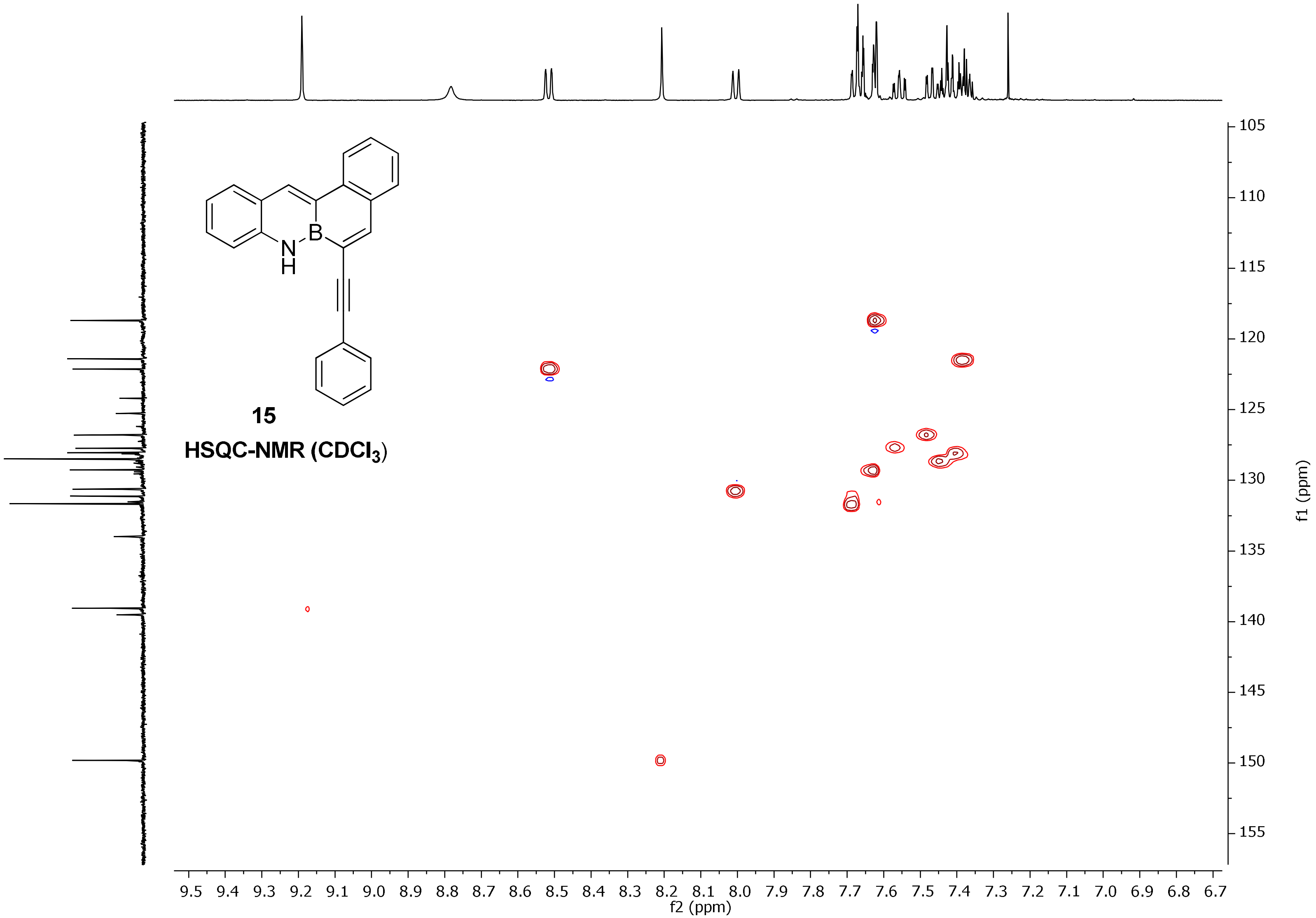

B
N
H
15
HSQC-NMR ($CDCl_3$)
f2 (ppm)
f1 (ppm)

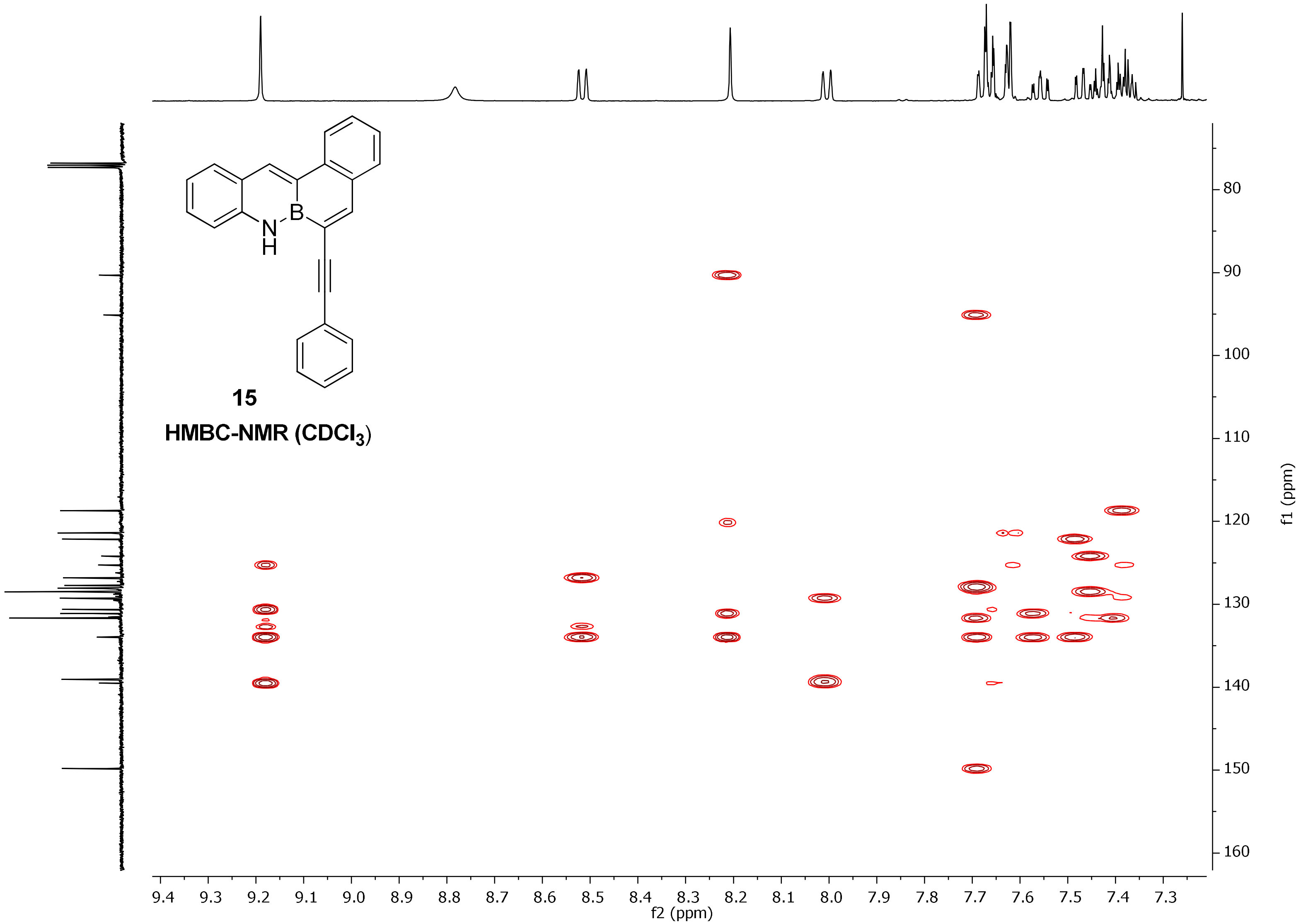

15
HMBC-NMR (CDCl3)
f2 (ppm)
f1 (ppm)

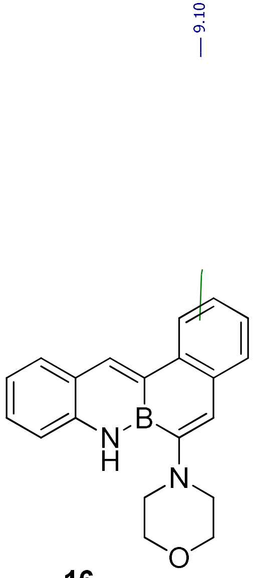

16

^{1}H-NMR (500 MHz, $CDCl_3$)

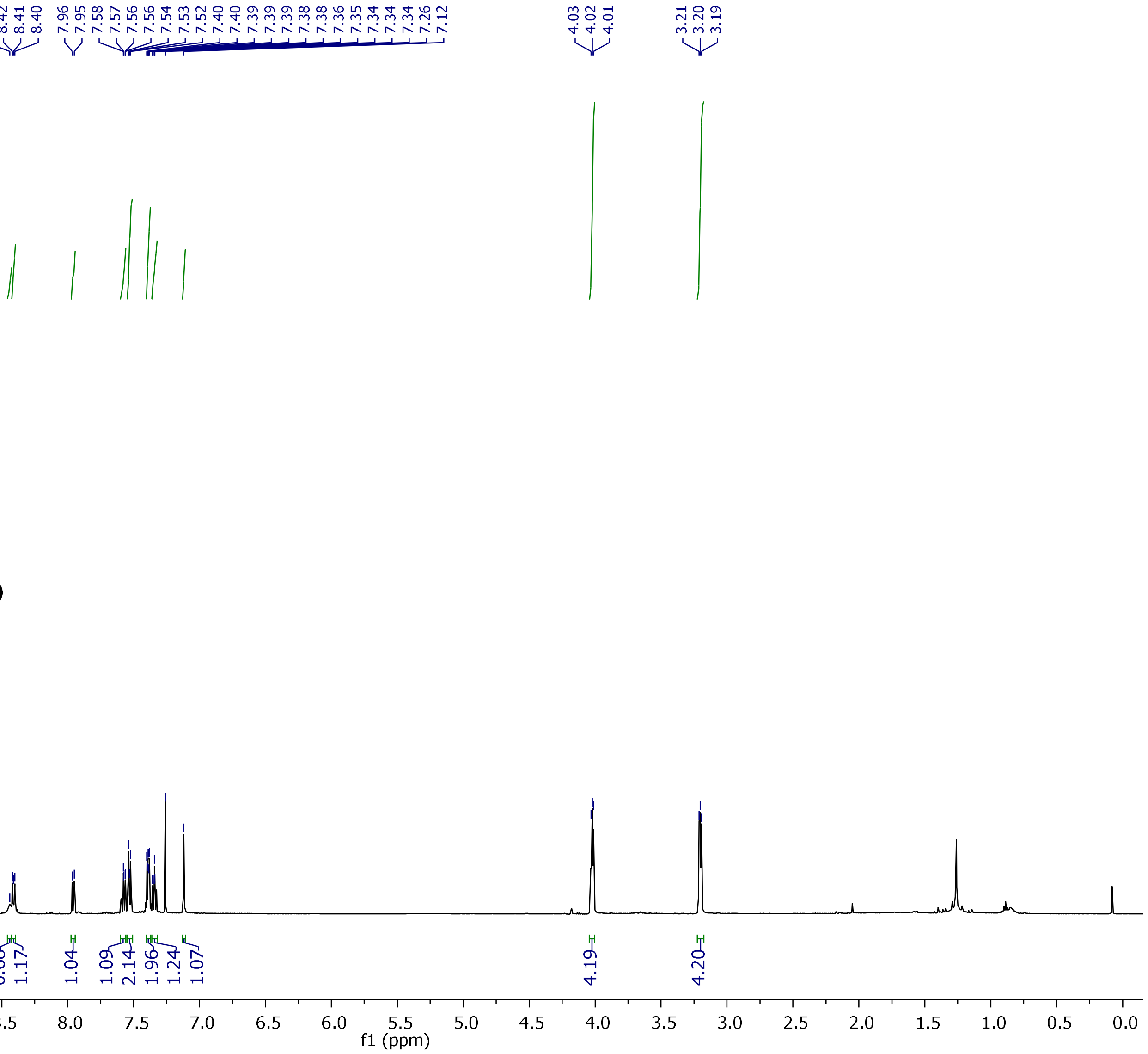

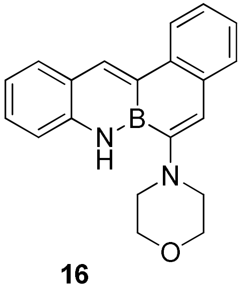

16

^{13}C-NMR (125 MHz, $CDCl_3$)

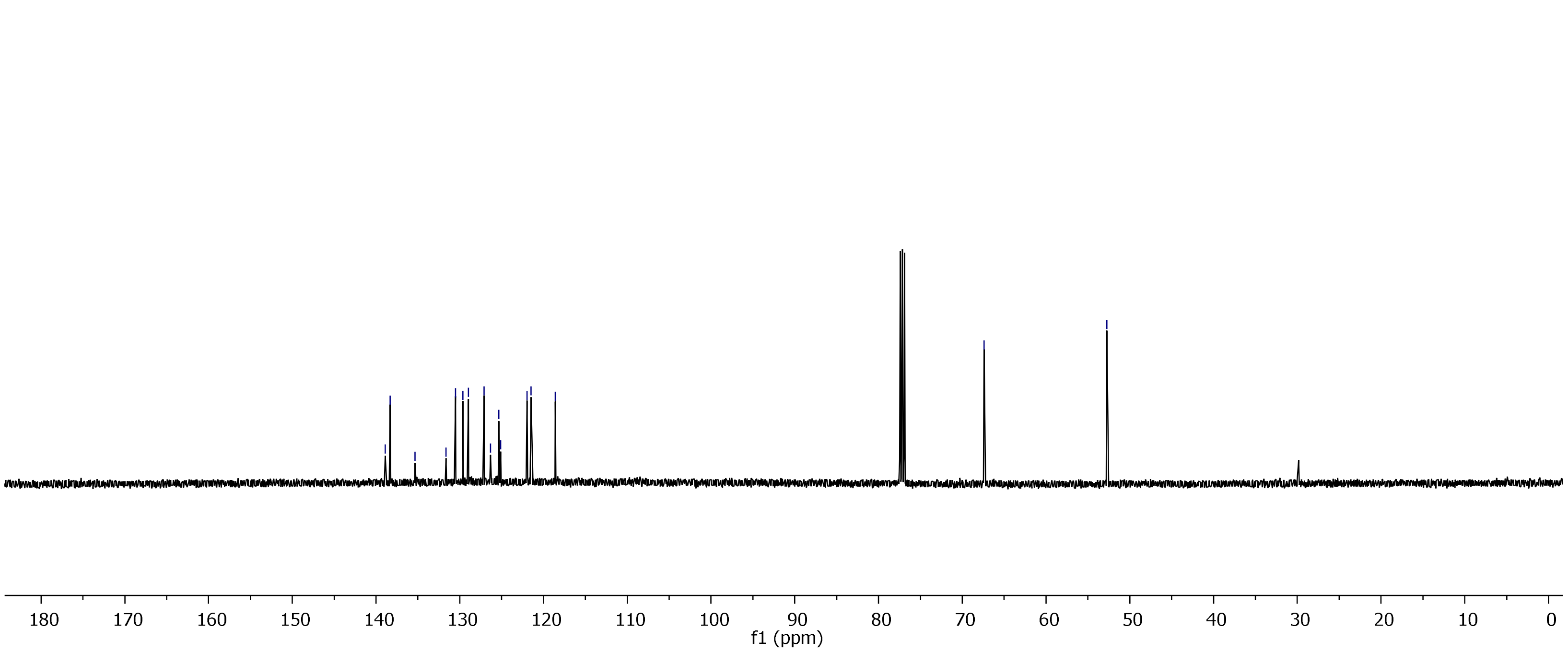

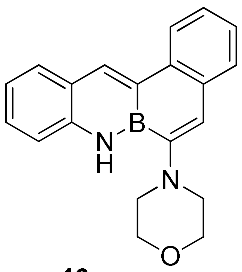

16

^{11}B-NMR (160 MHz, $CDCl_3$)

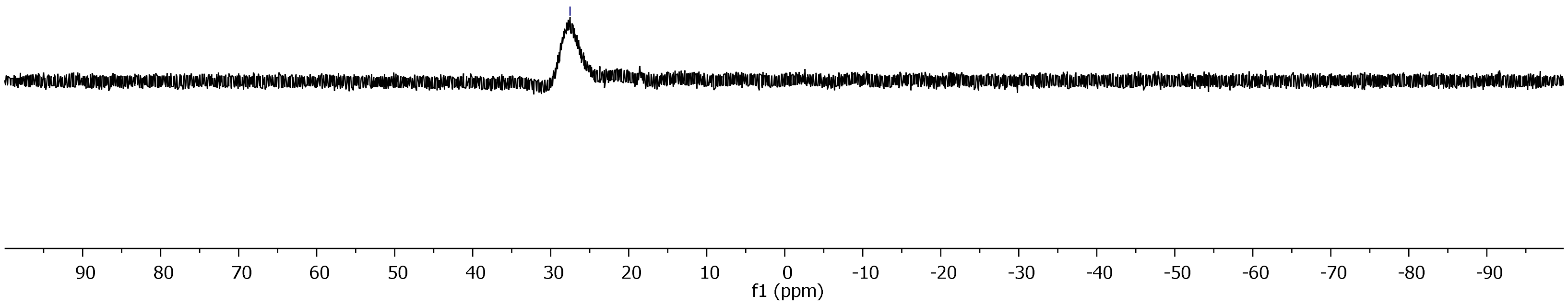

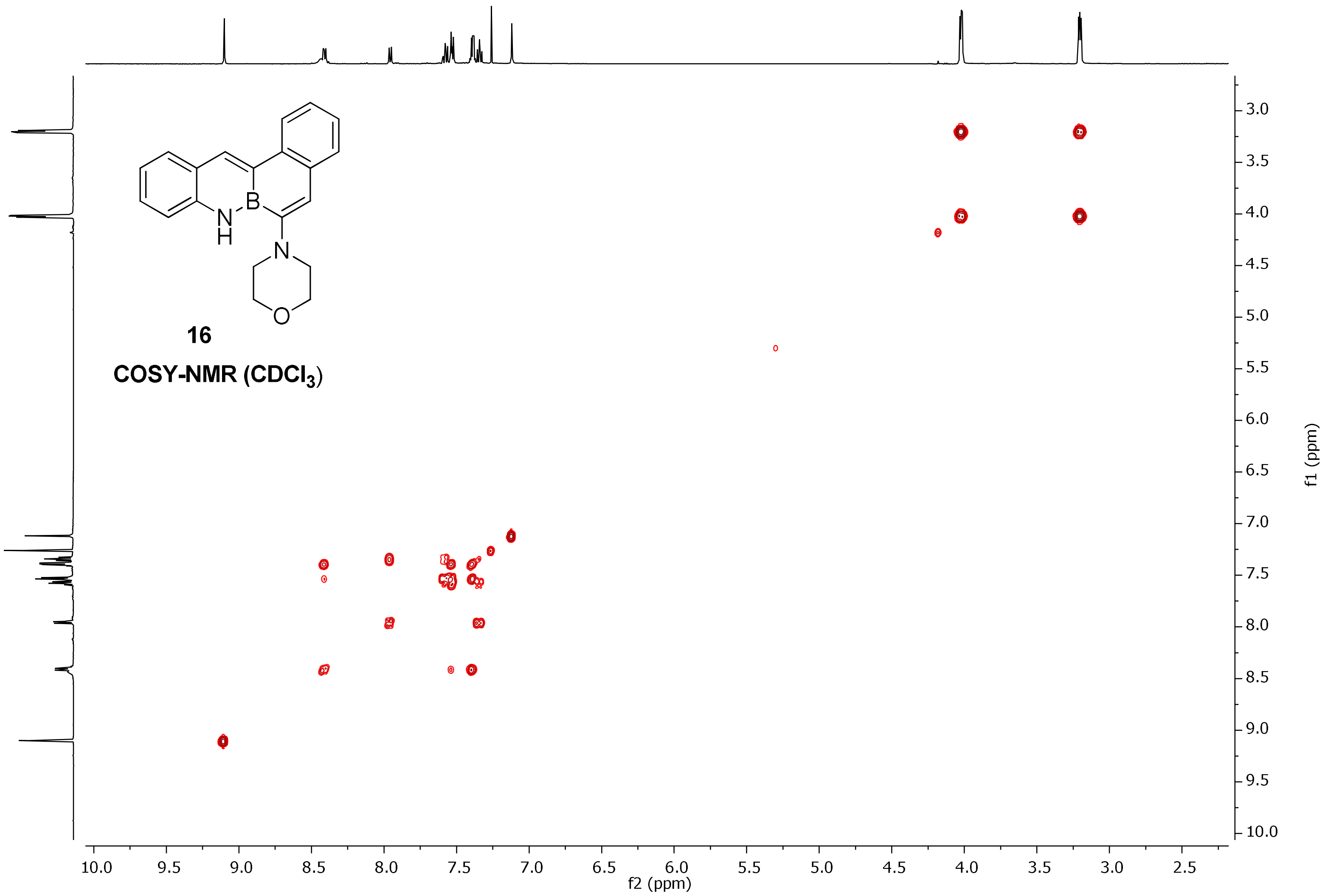
16
COSY-NMR ($CDCl_3$)
f2 (ppm)
f1 (ppm)

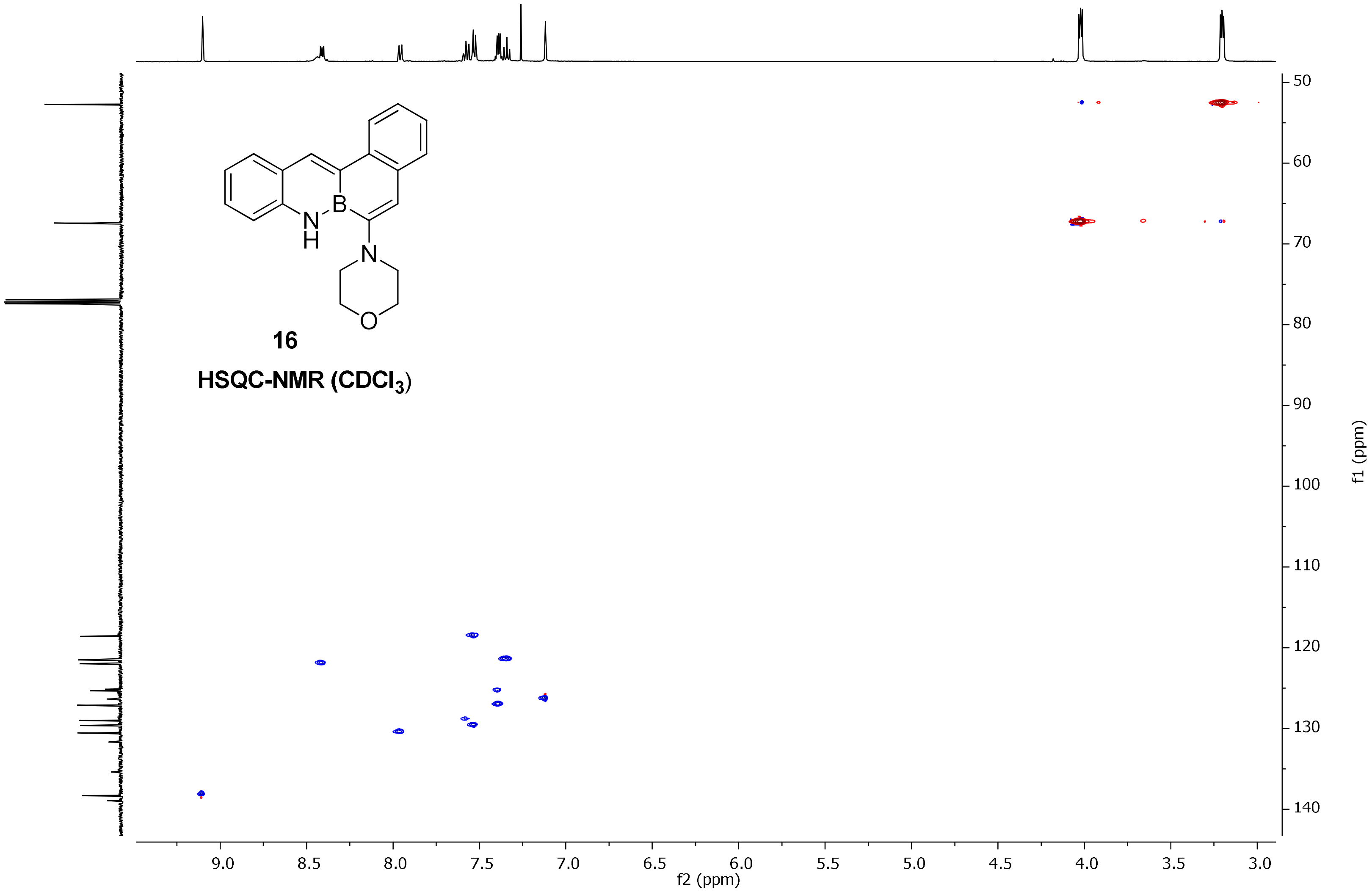

N
B
H
N
O
16
HSQC-NMR (CDCl3)
f2 (ppm)
f1 (ppm)
9.0
8.5
8.0
7.5
7.0
6.5
6.0
5.5
5.0
4.5
4.0
3.5
3.0
50
60
70
80
90
100
110
120
130
140

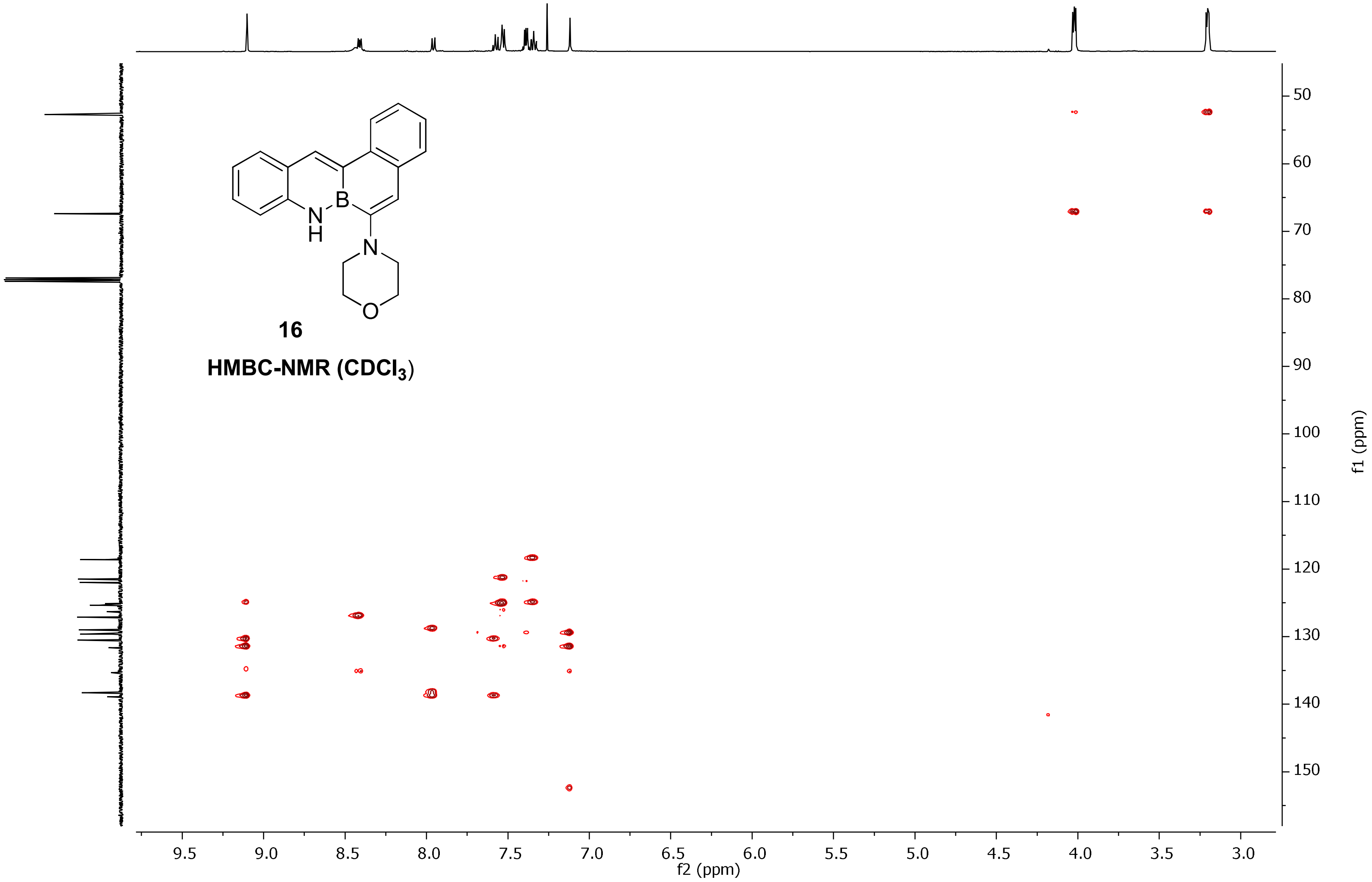

16

HMBC-NMR ($CDCl_3$)

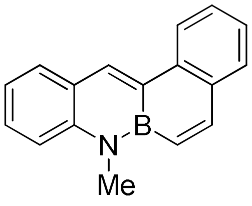

17

^{1}H-NMR (500 MHz, $CDCl_3$)

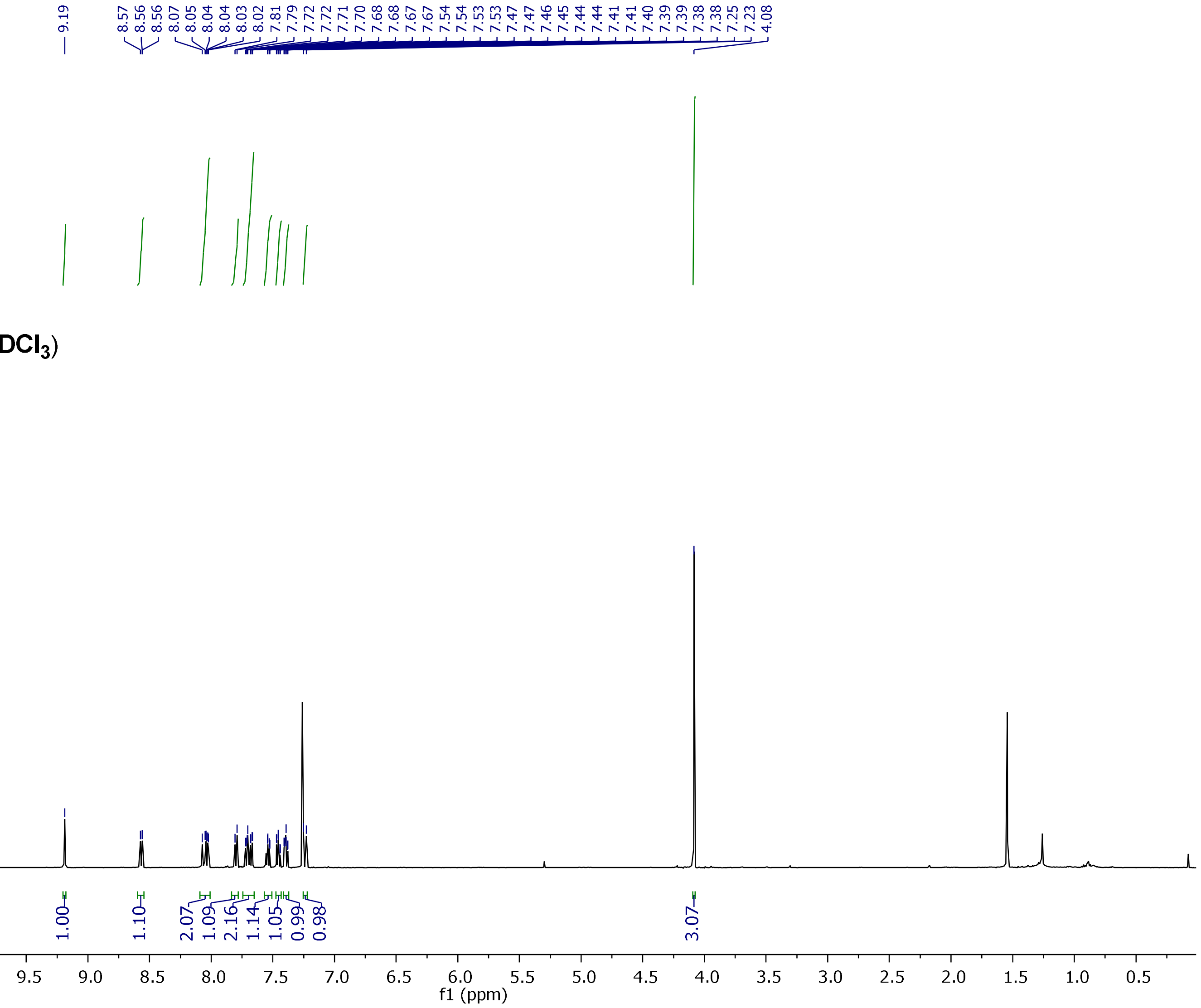

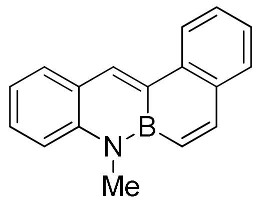

17

^{13}C-NMR (125 MHz, $CDCl_3$)

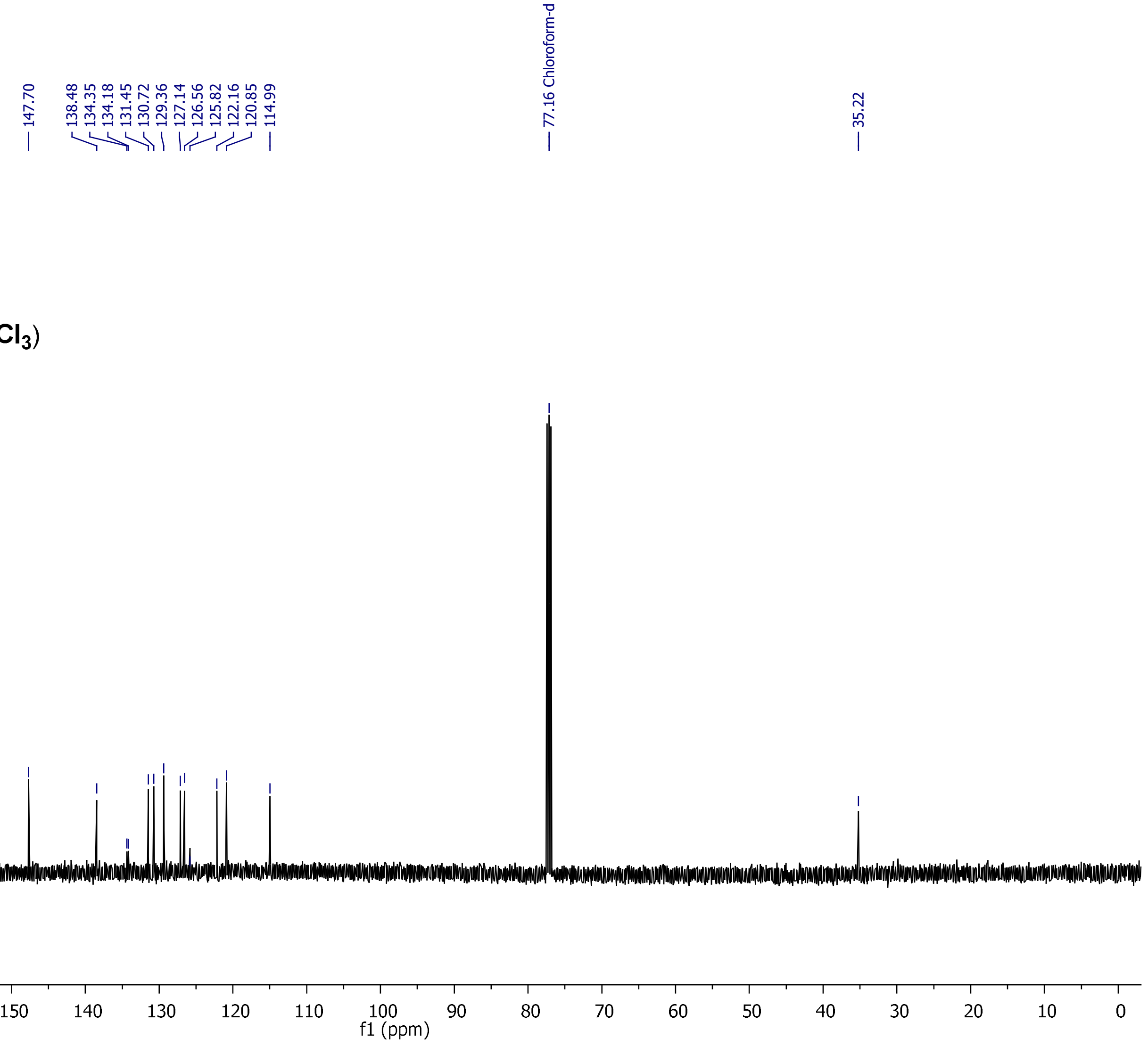

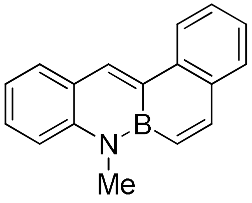

17

^{11}B-NMR (160 MHz, $CDCl_3$)

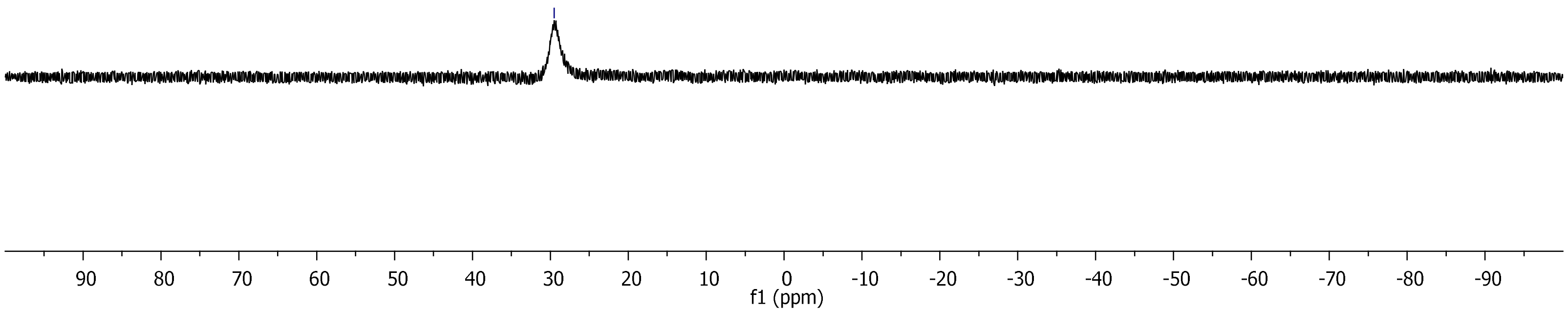

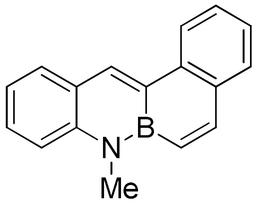

17

COSY-NMR ($CDCl_3$)

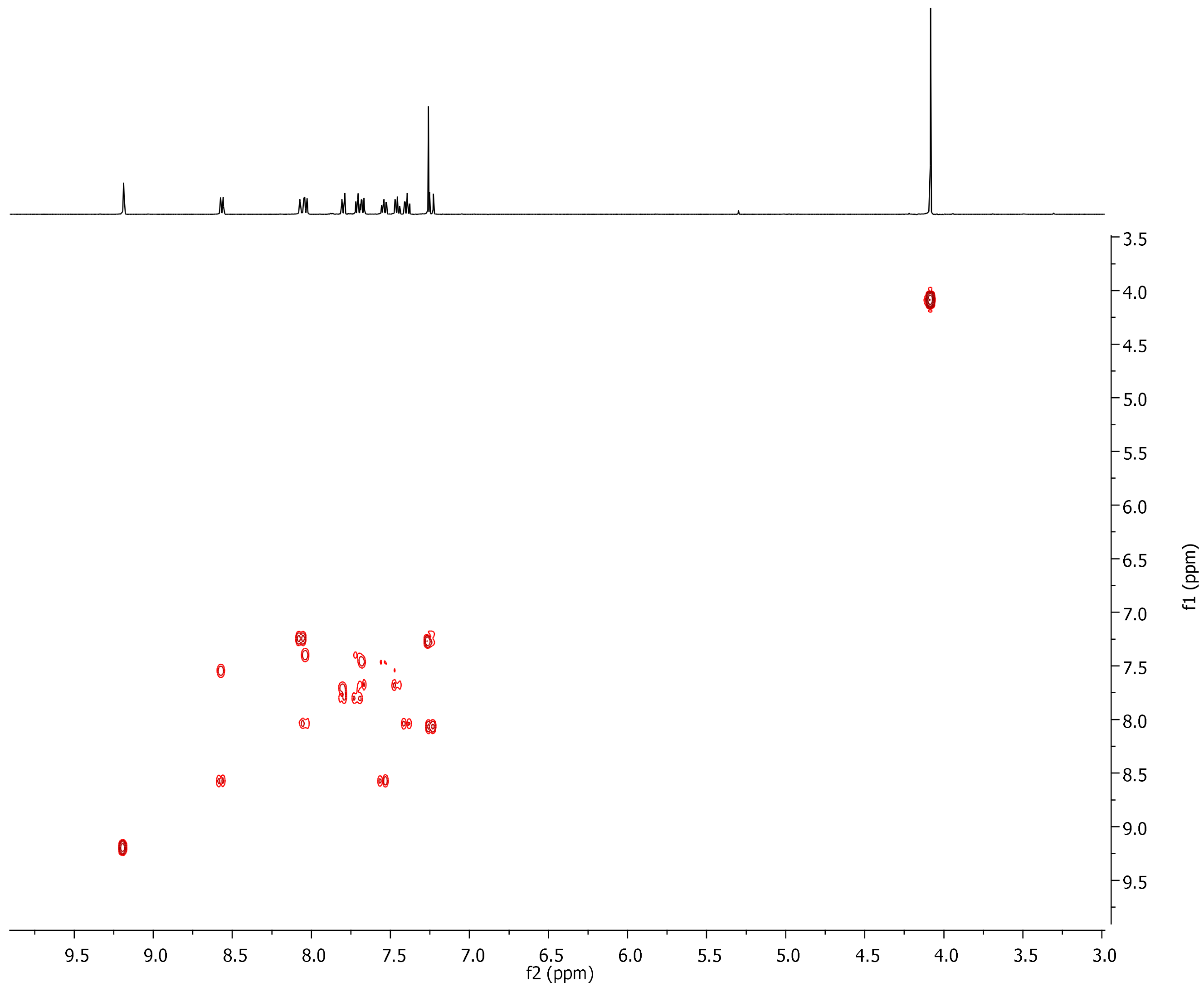

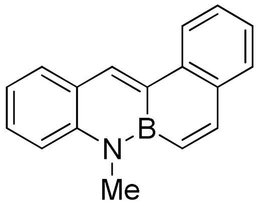

17

HSQC-NMR ($CDCl_3$)

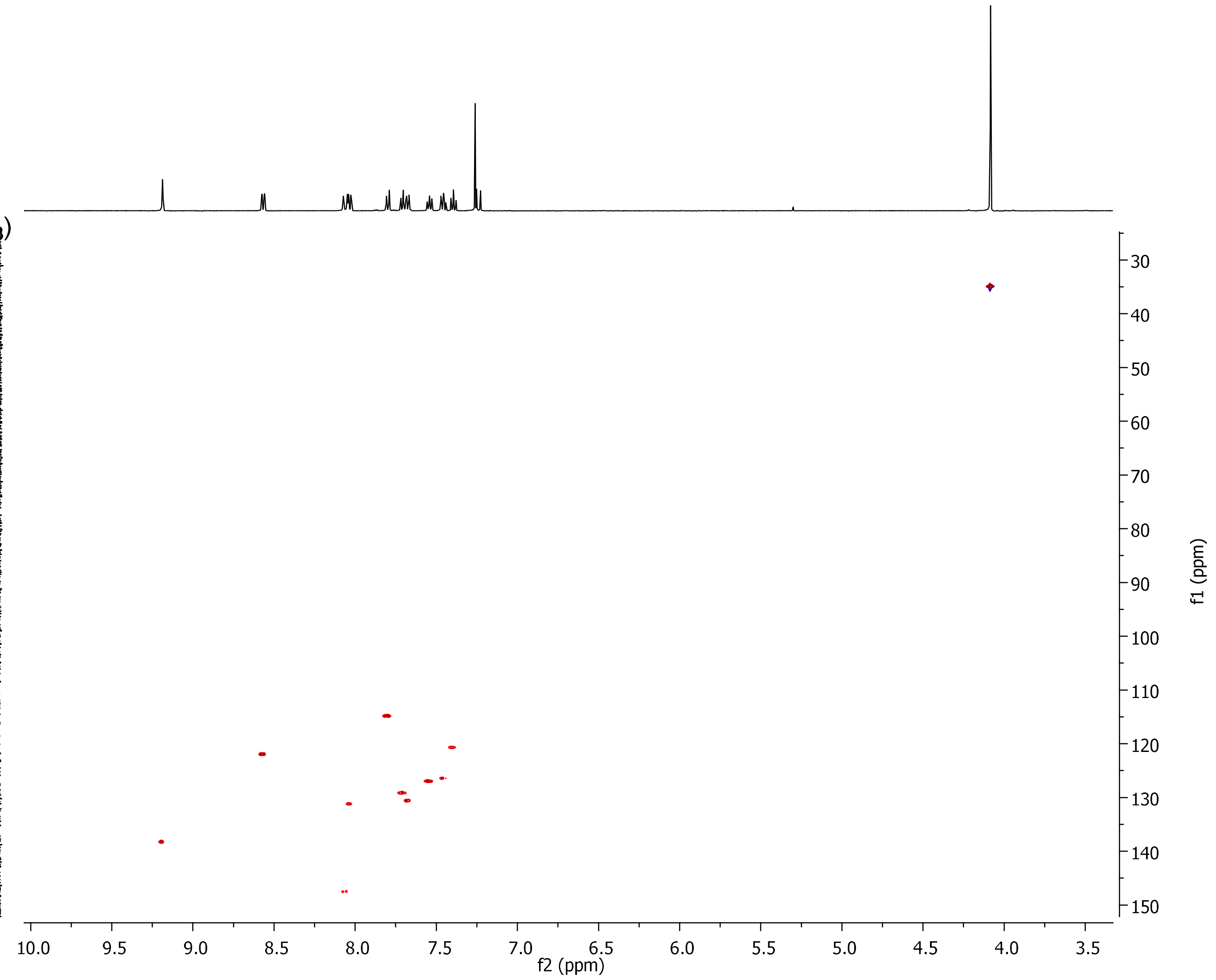

Supplement: Supplementary file 1 — jo1c01095_si_001.pdf [file jo1c01095_si_001.pdf]
